# Supplementary material for: Synthesis, Solution, and Solid State Properties of Homological Dialkylated Naphthalene Diimides—A Systematic Review of Molecules for Next-Generation Organic Electronics
Source: Molecules. 2023 Mar 25;28(7):2940. doi: 10.3390/molecules28072940 (PMC10096413; doi:10.3390/molecules28072940)
Supplement: Supplementary file 1 [file molecules-28-02940-s001.zip › NDI_derivatives_Chlebosz_supporting information_NMR_final_R1.pdf]

Supporting Information to the article (part 2)

Synthesis, Solution and Solid State Properties of Homological Dialkylated Naphthalene Diimides. A Systematic Review of Molecules for Next-Generation Organic Electronics

Dorota Chlebosz[1,2,], Waldemar Goldeman[3], Krzysztof Janus[1,2], Michał Szuster[1], Adam Kiersnowski[1,2,*]

[1] Department of Physical and Quantum Chemistry, Wroclaw University of Science and Technology, Wybrzeze Wyspianskiego 27, 50-370 Wroclaw, Poland

[2] The Leibniz Institute of Polymer Research, Hohe Strasse 6, D-01069 Dresden, Germany

[3] Department of Medicinal and Organic Chemistry, Wroclaw University of Science and Technology, Wybrzeze Wyspianskiego 27, 50-370 Wroclaw, Poland

*correspondence: Adam Kiersnowski, adam.kiersnowski@pwr.edu.pl

^{1}H and ^{13}C NMR spectra of all NDIs

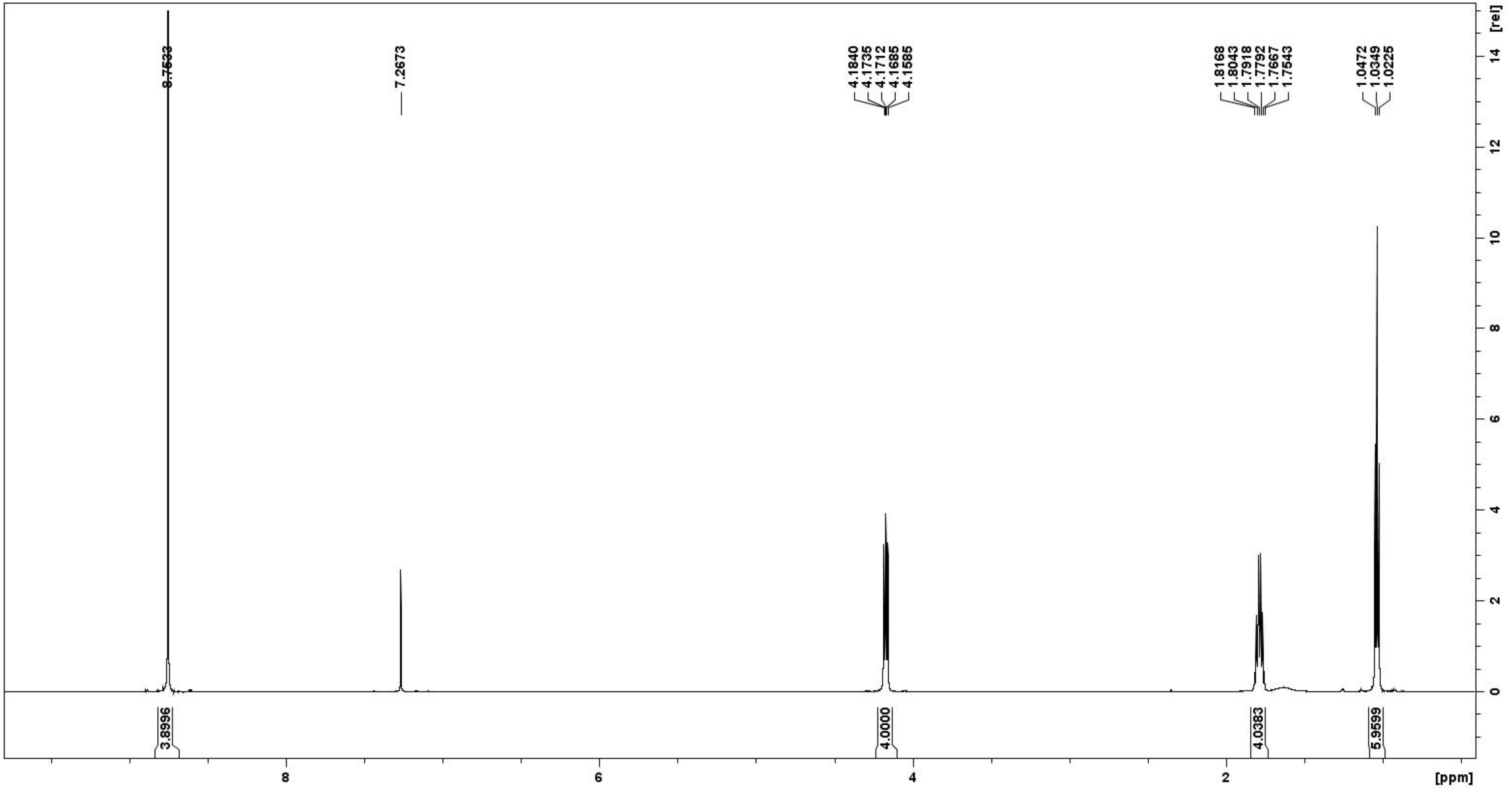

^{1}H NMR spectrum of NDIC3 ($CDCl_3$, 600MHz)

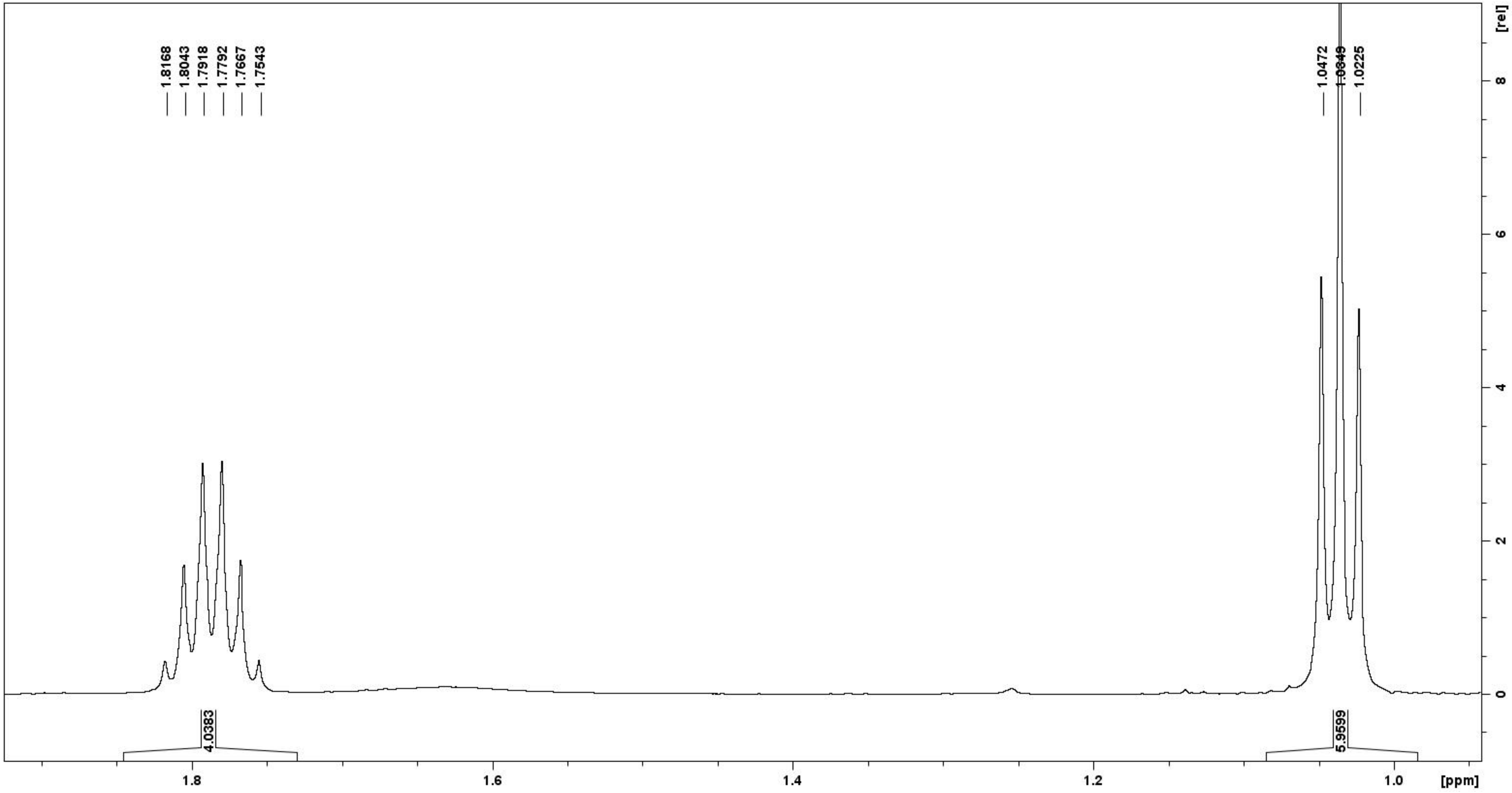

Expanded aliphatic region of ^{1}H NMR spectrum of NDIC3.

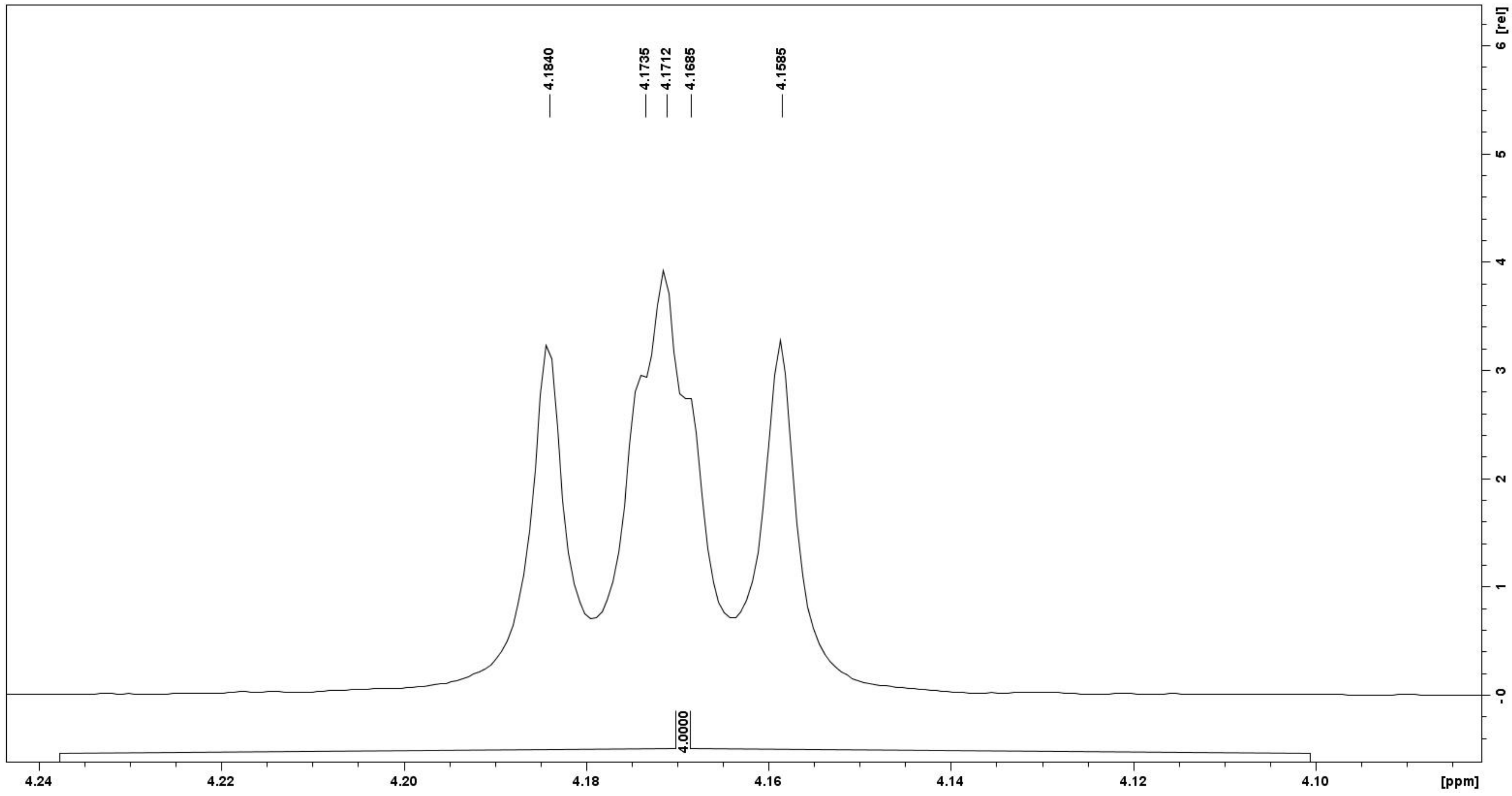

Expanded aliphatic region of ^{1}H NMR spectrum of NDIC3

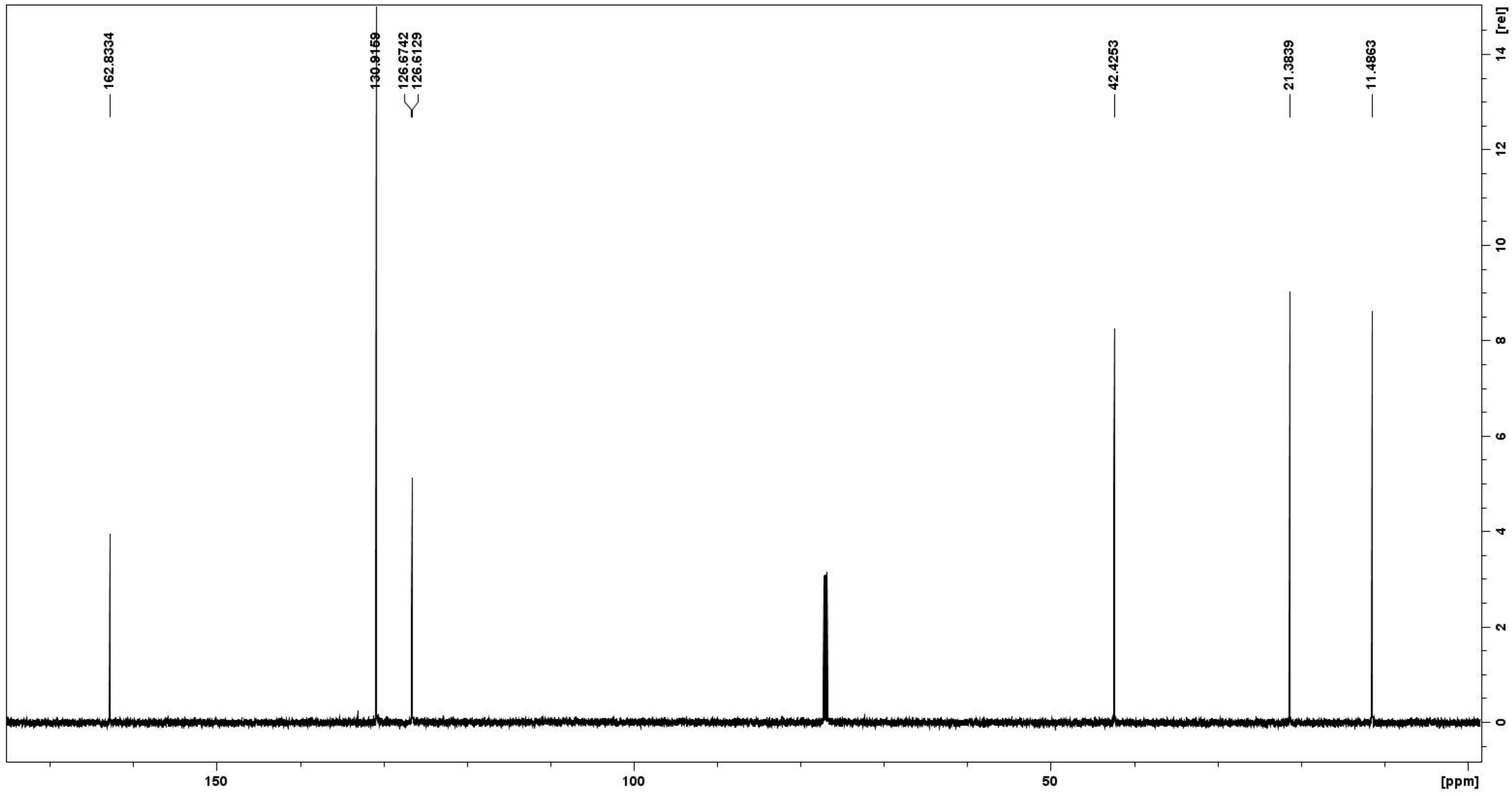

$^{13}C\{^{1}H\}$NMR spectrum of NDIC3 ($CDCl_3$, 151MHz)

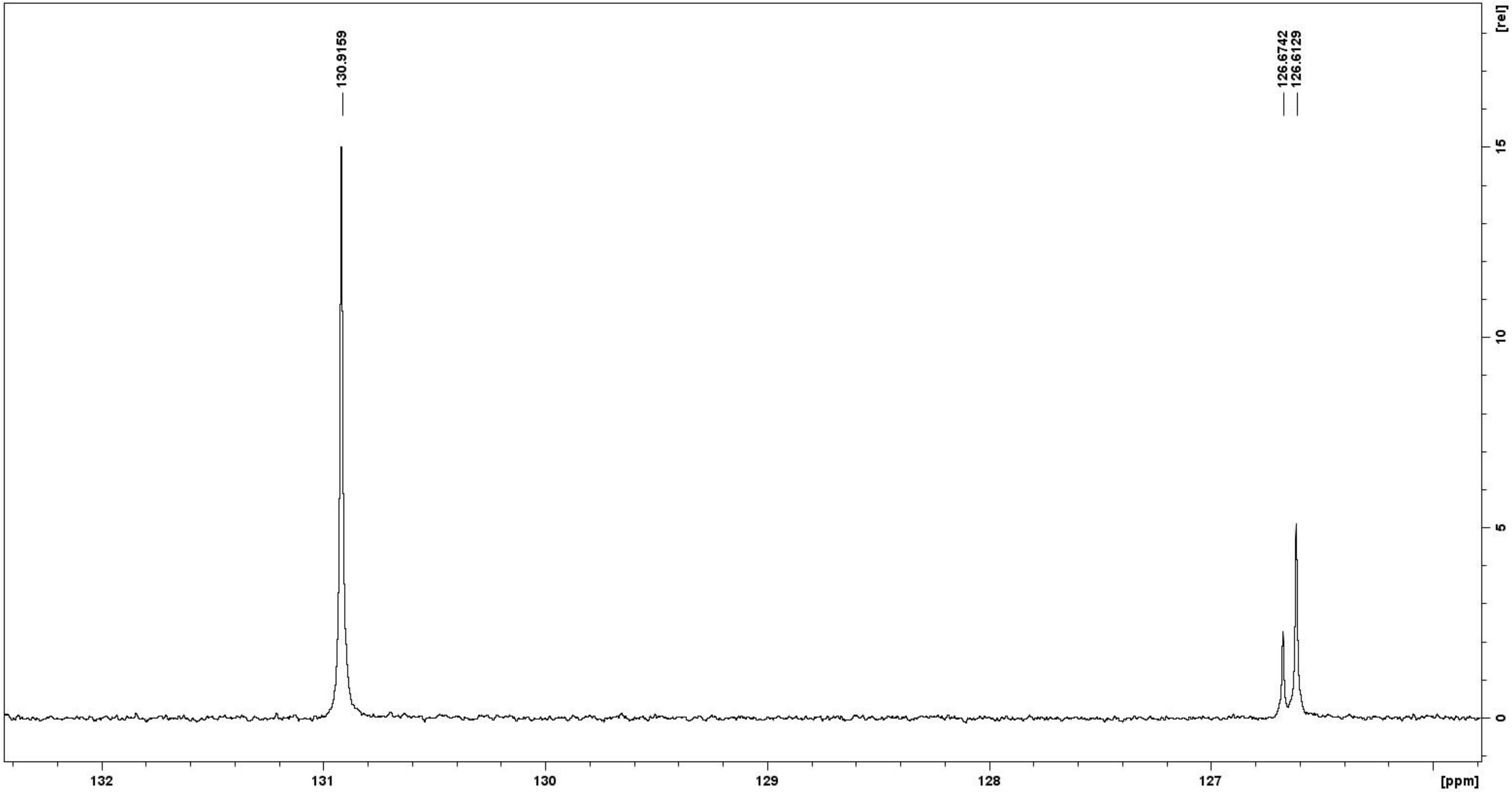

Expanded aromatic region of $^{13}C\{^{1}H\}$NMR spectrum of NDIC3

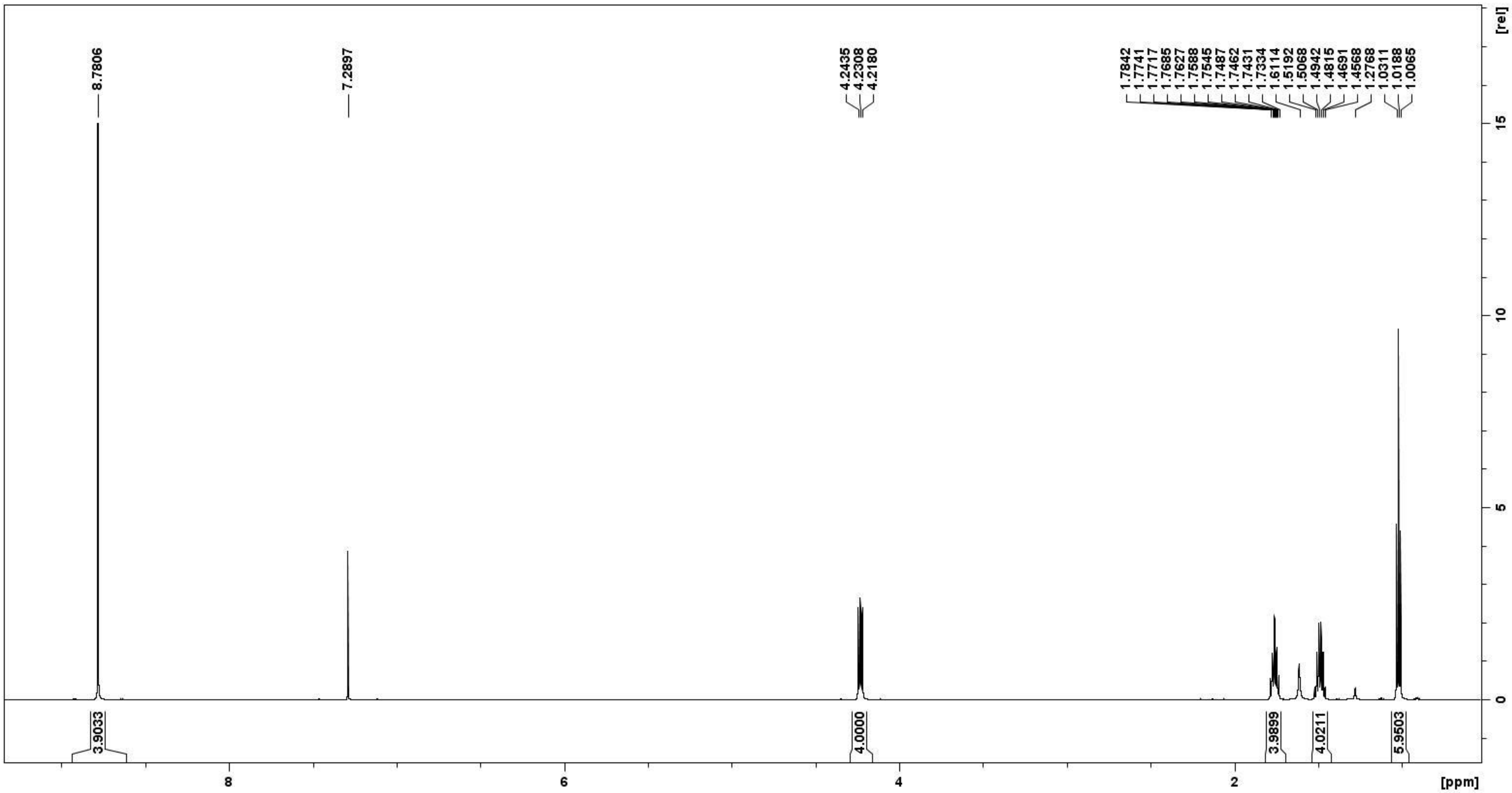

^{1}H NMR spectrum of NDIC4 ($CDCl_3$, 600MHz)

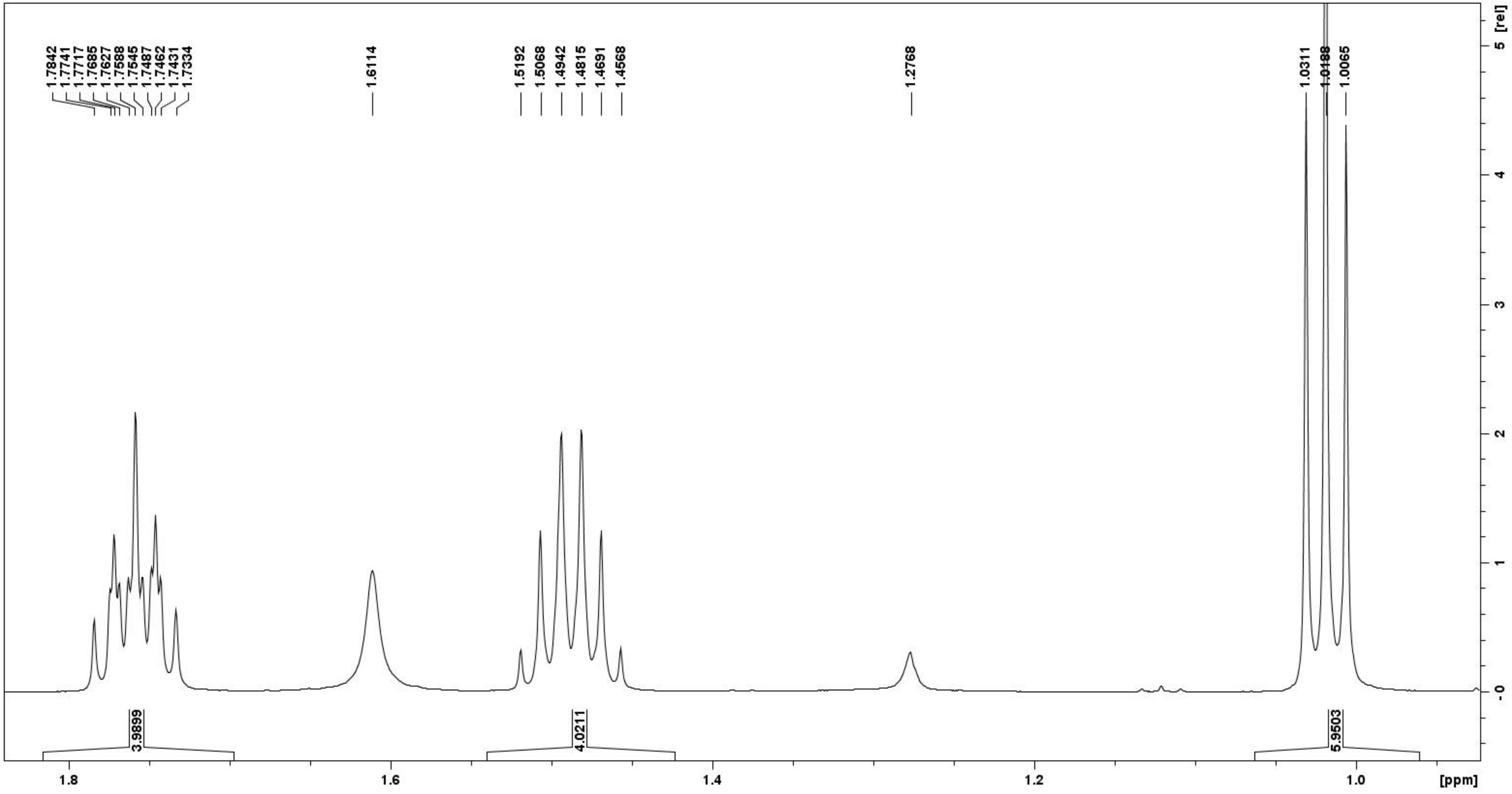

Expanded aliphatic region of ^{1}H NMR spectrum of NDIC4

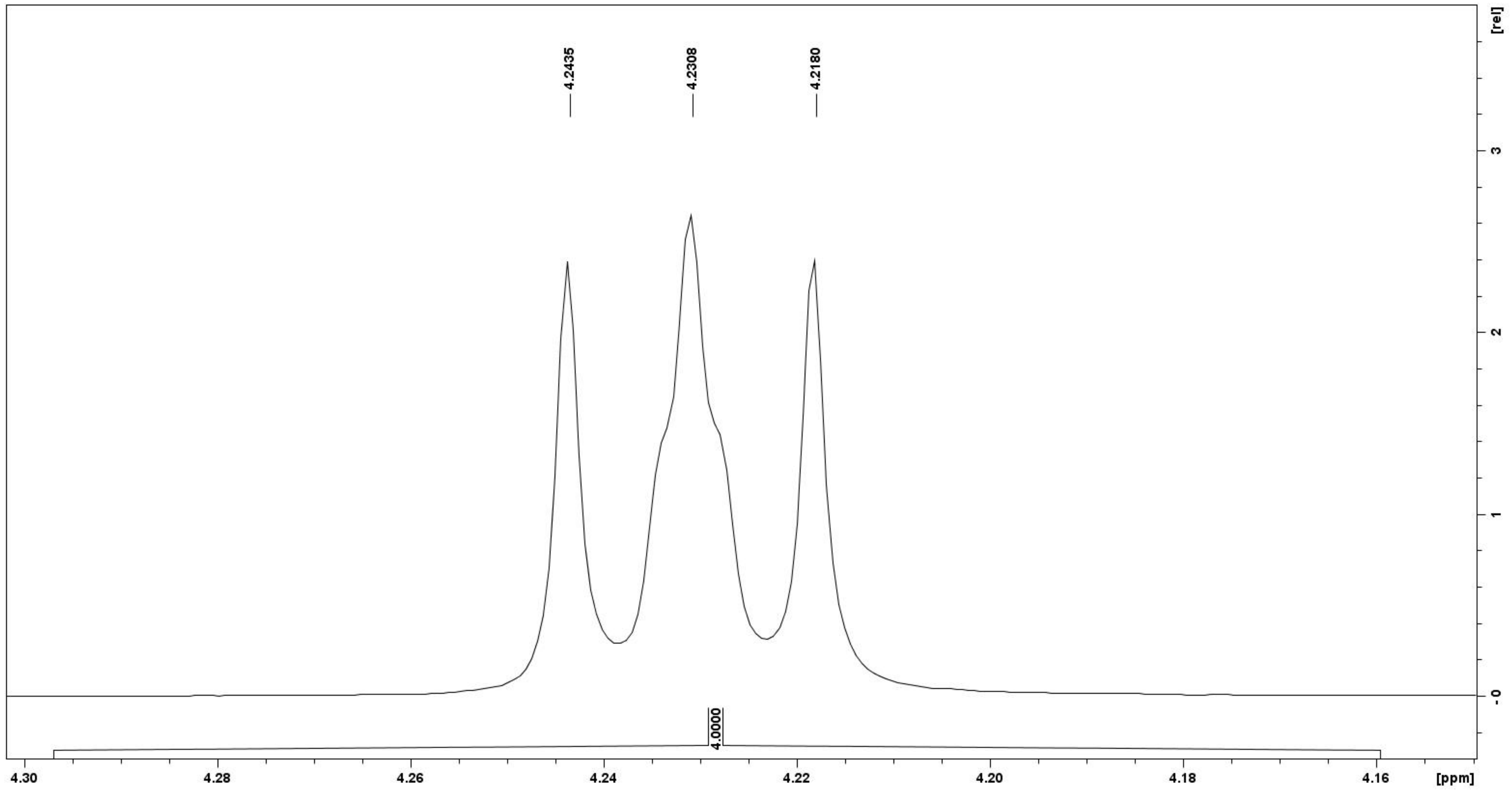

Expanded aliphatic region of ^{1}H NMR spectrum of NDIC4

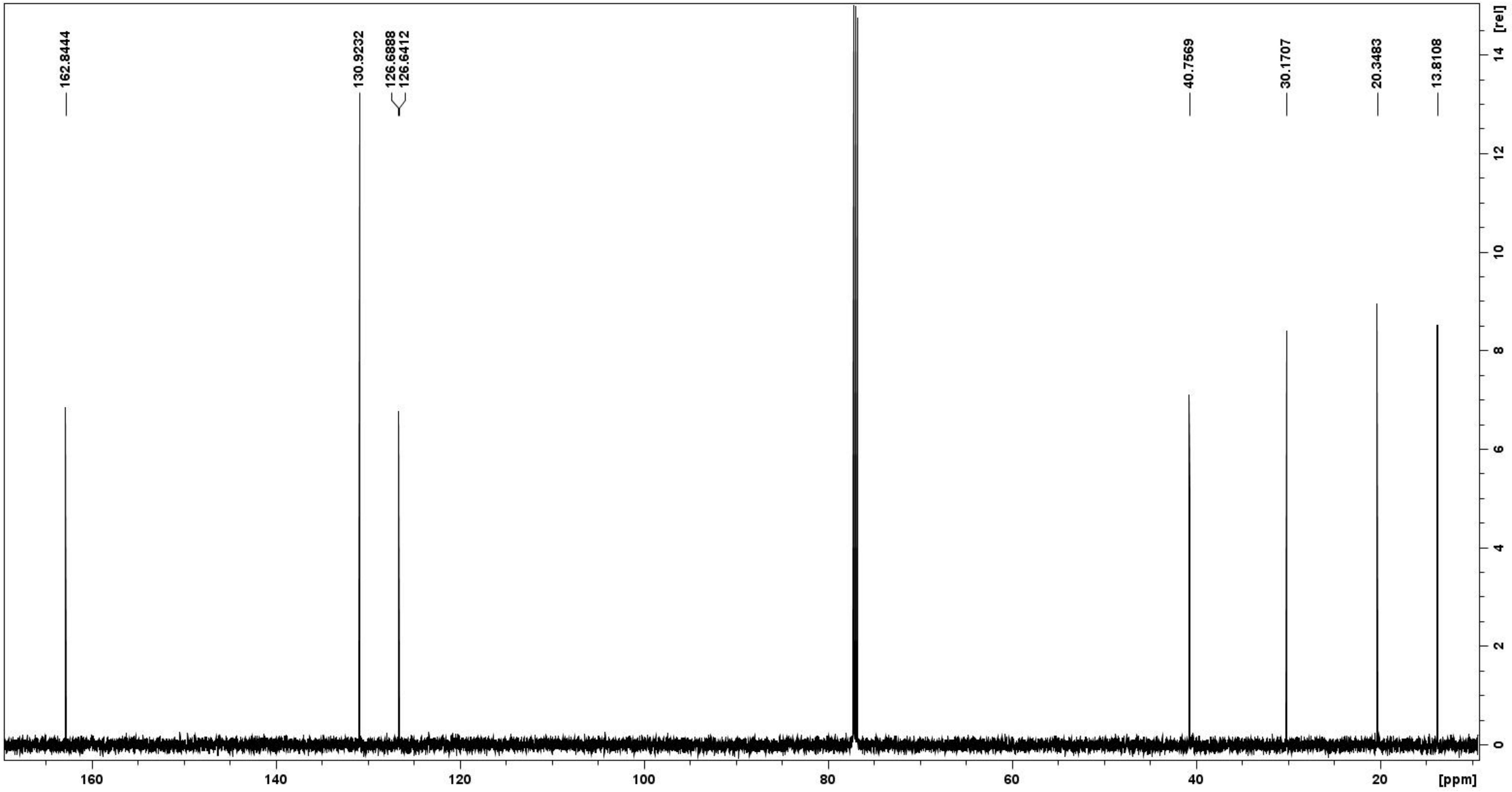

$^{13}C\{^1H\}$NMR spectrum of NDIC4 ($CDCl_3$, 151MHz)

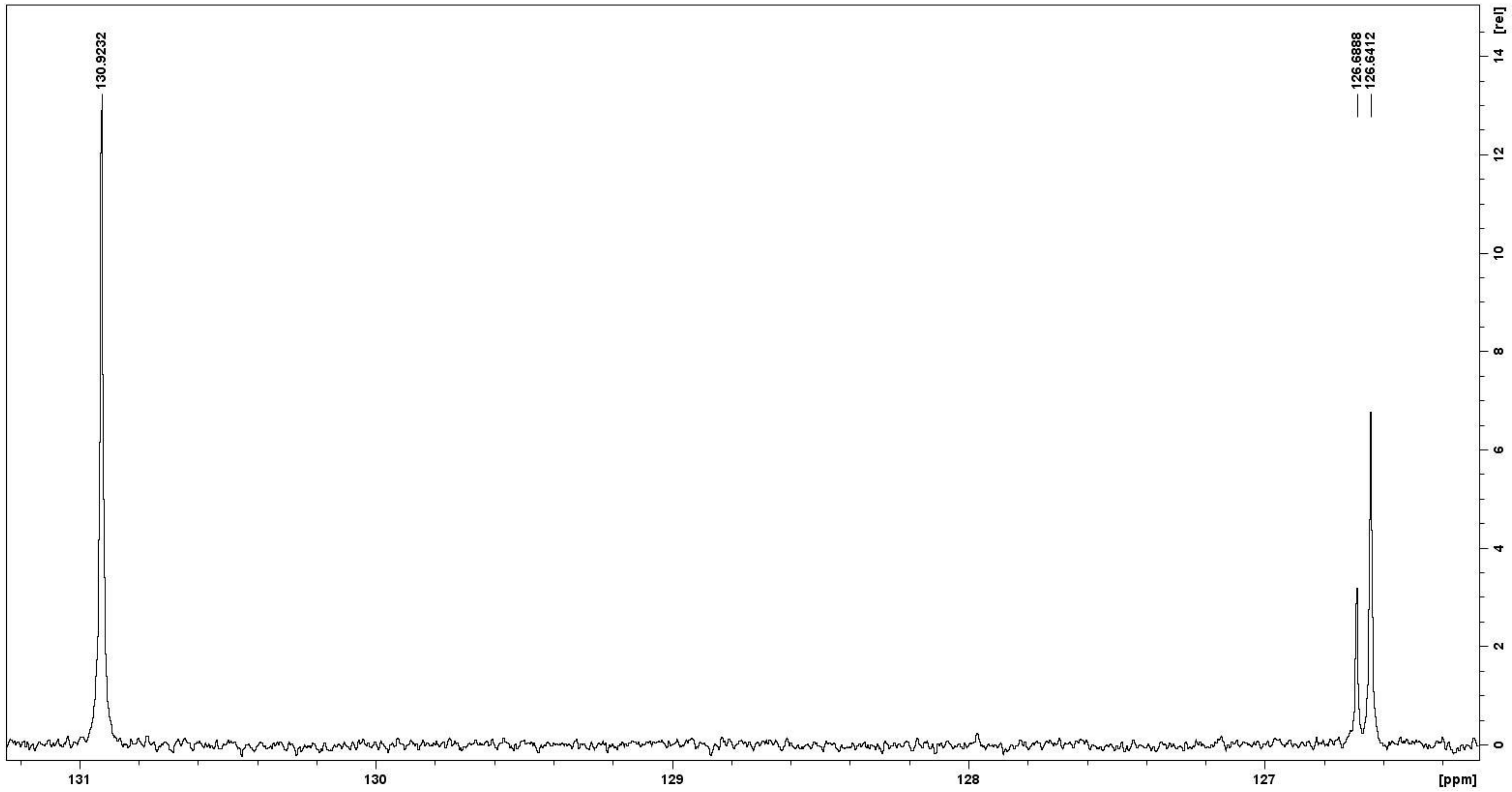

Expanded aromatic region of $^{13}C\{^{1}H\}$NMR spectrum of NDIC4

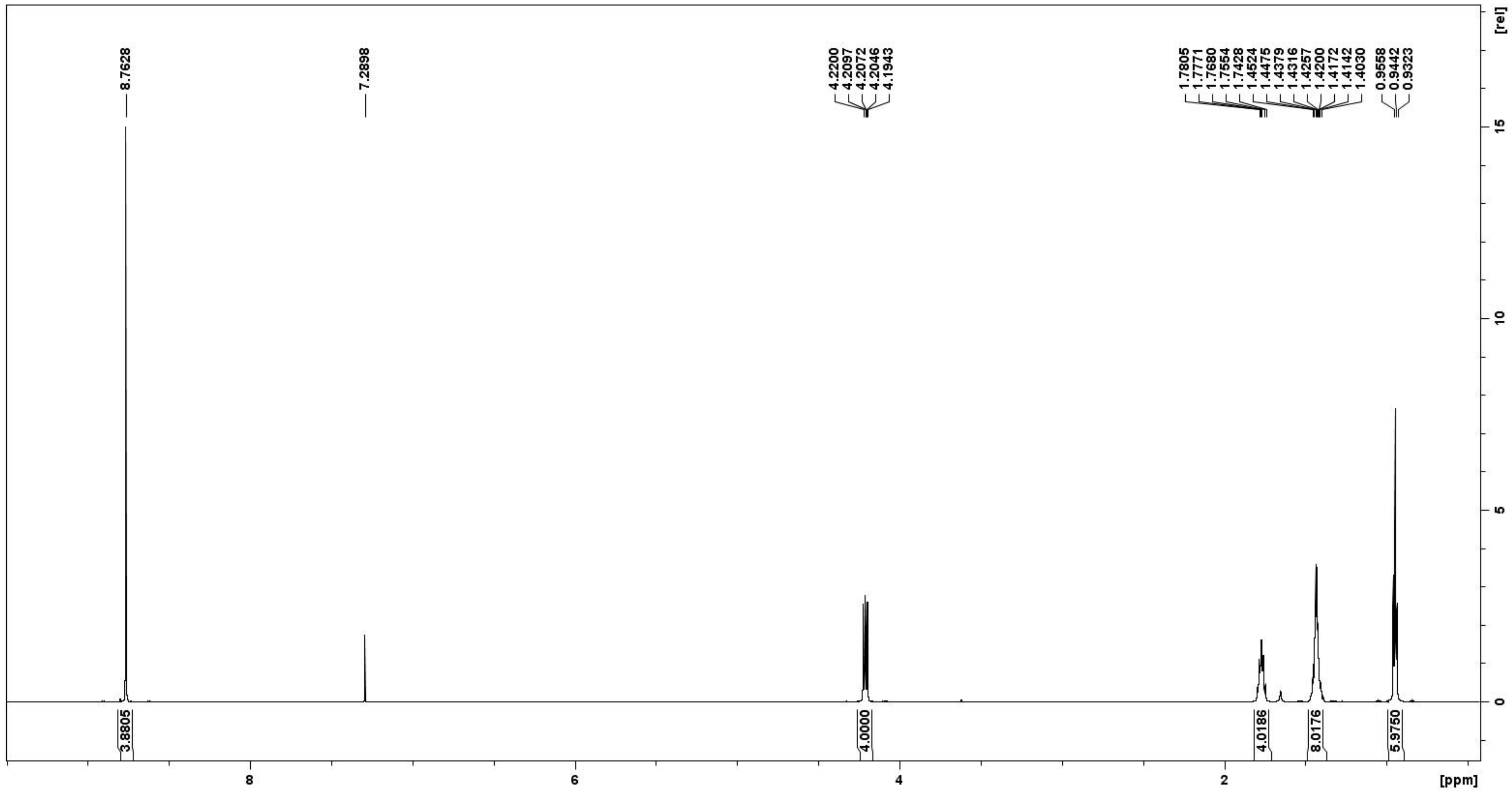

^1H NMR spectrum of NDIC5 ($CDCl_3$, 600MHz)

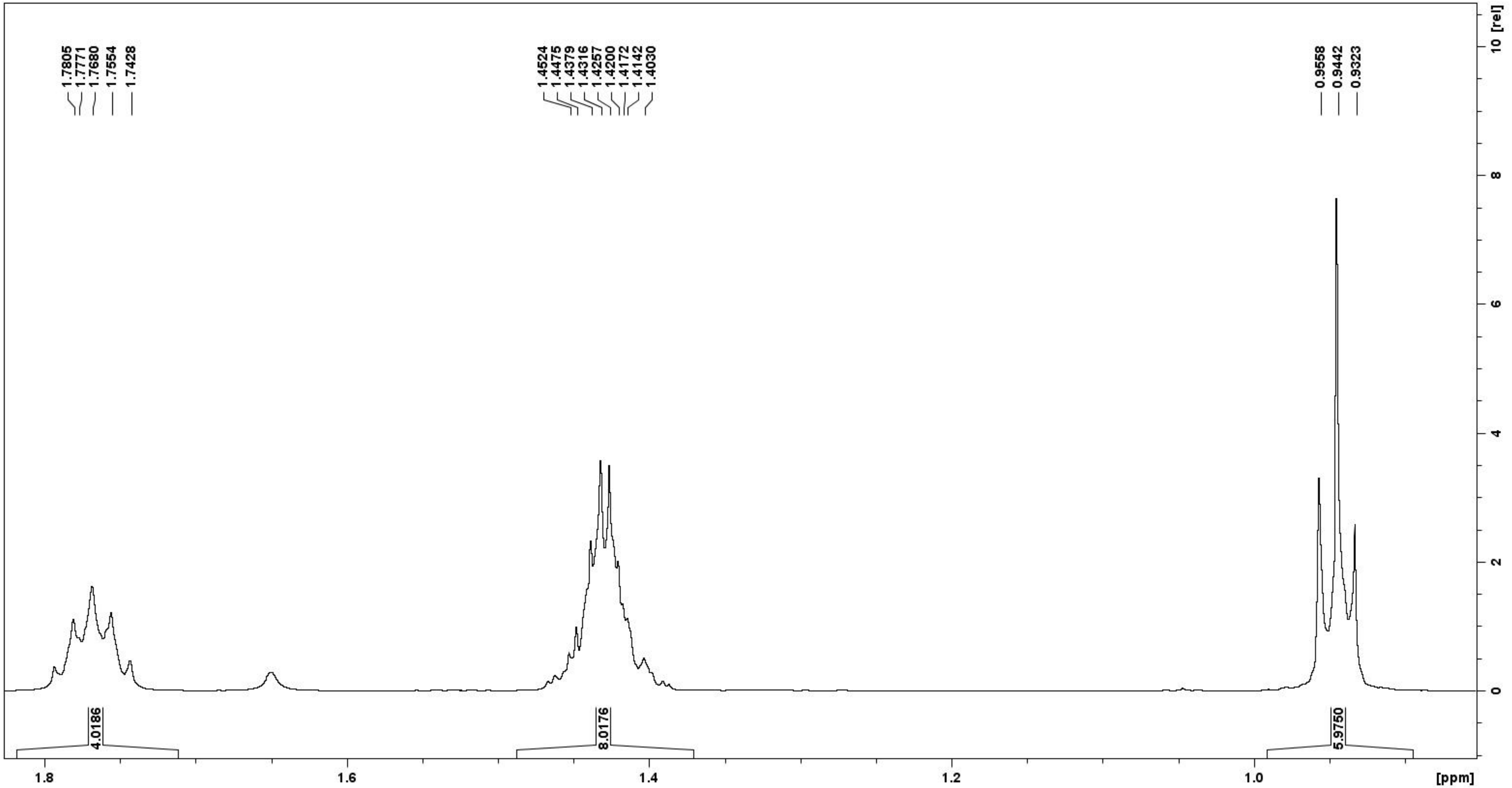

Expanded aliphatic region of ^{1}H NMR spectrum of NDIC5

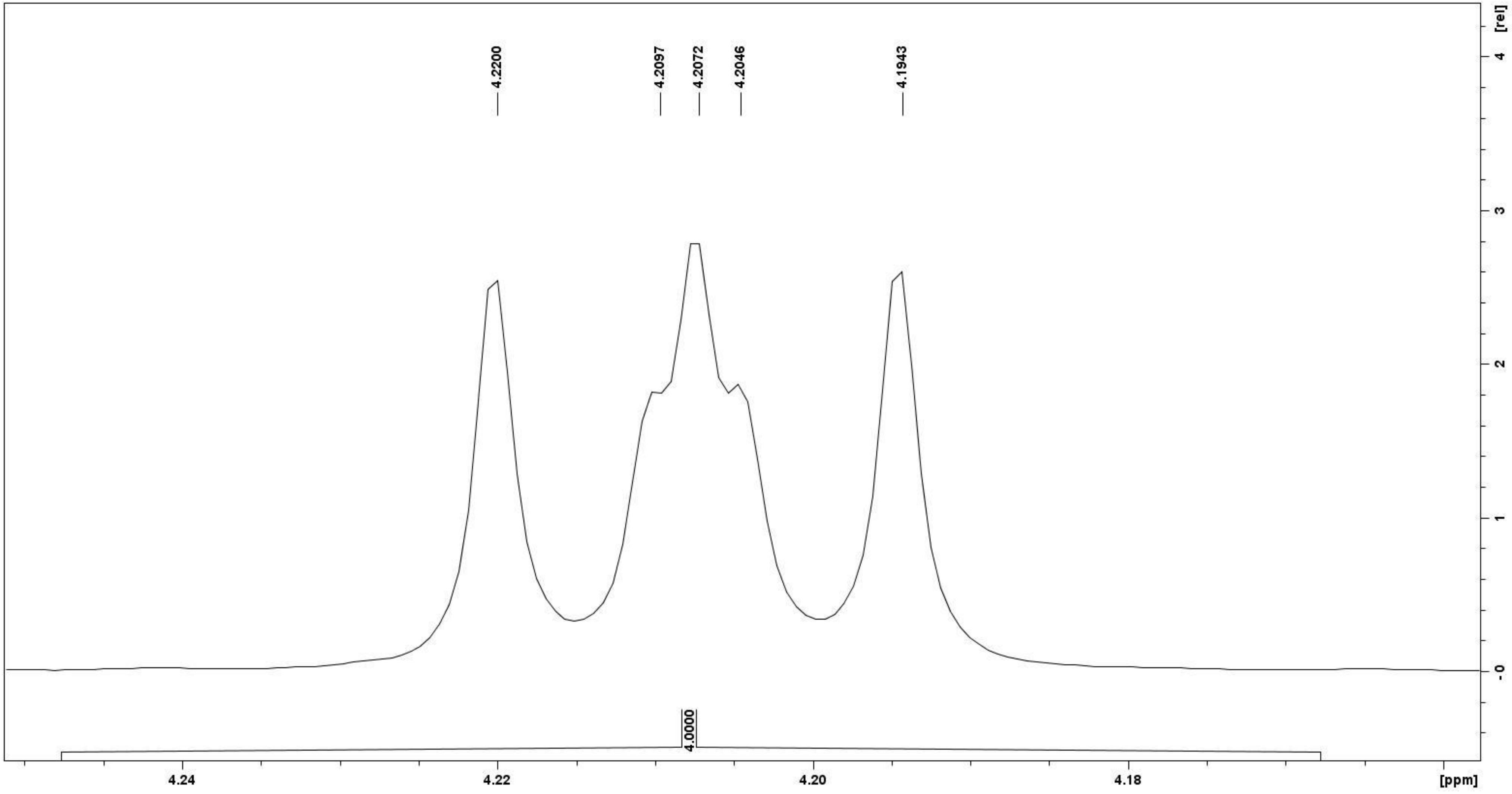

Expanded aliphatic region of ^{1}H NMR spectrum of NDIC5

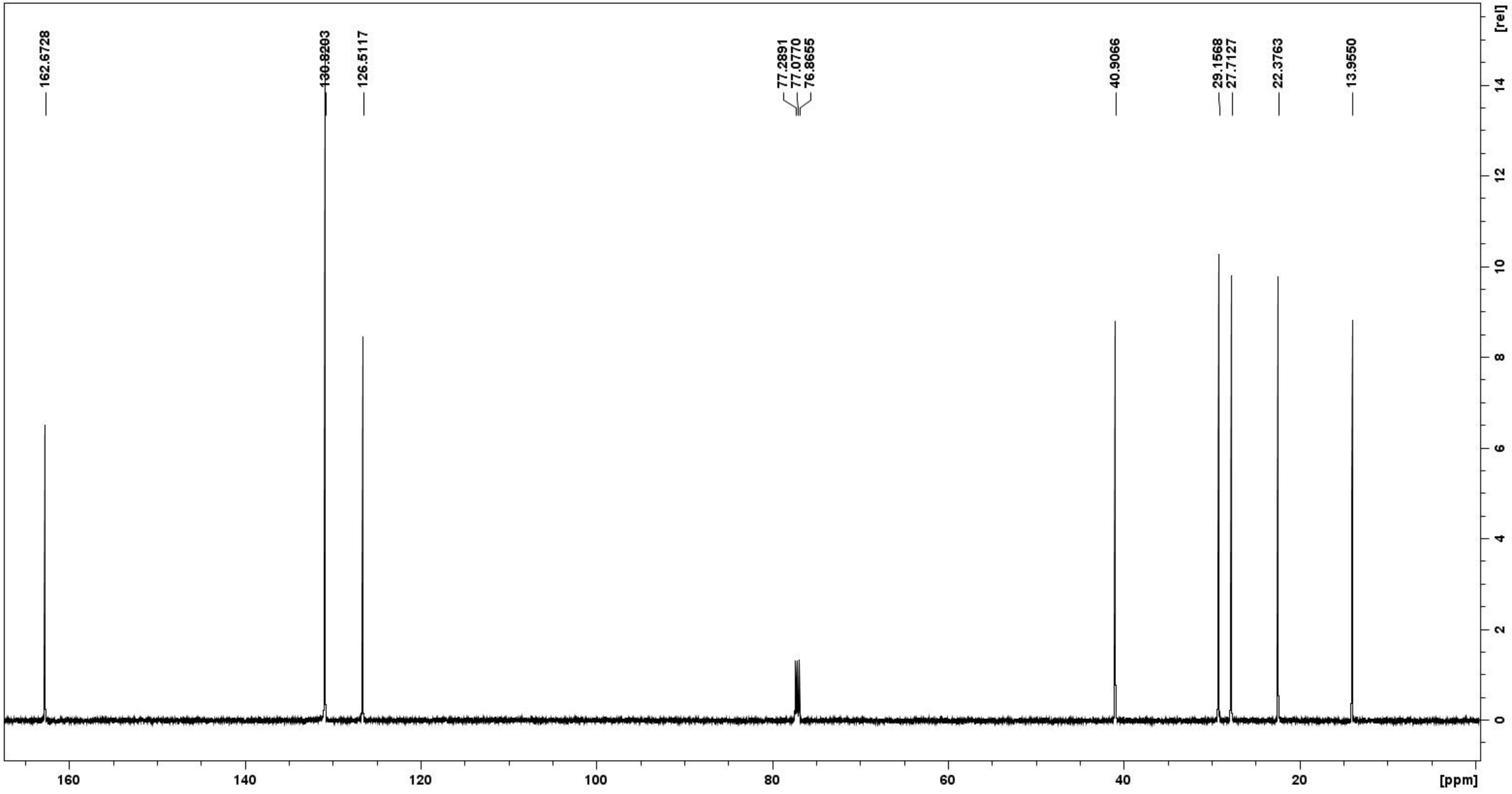

$^{13}C\{^{1}H\}$NMR spectrum of NDIC5 ($CDCl_3$, 151MHz)

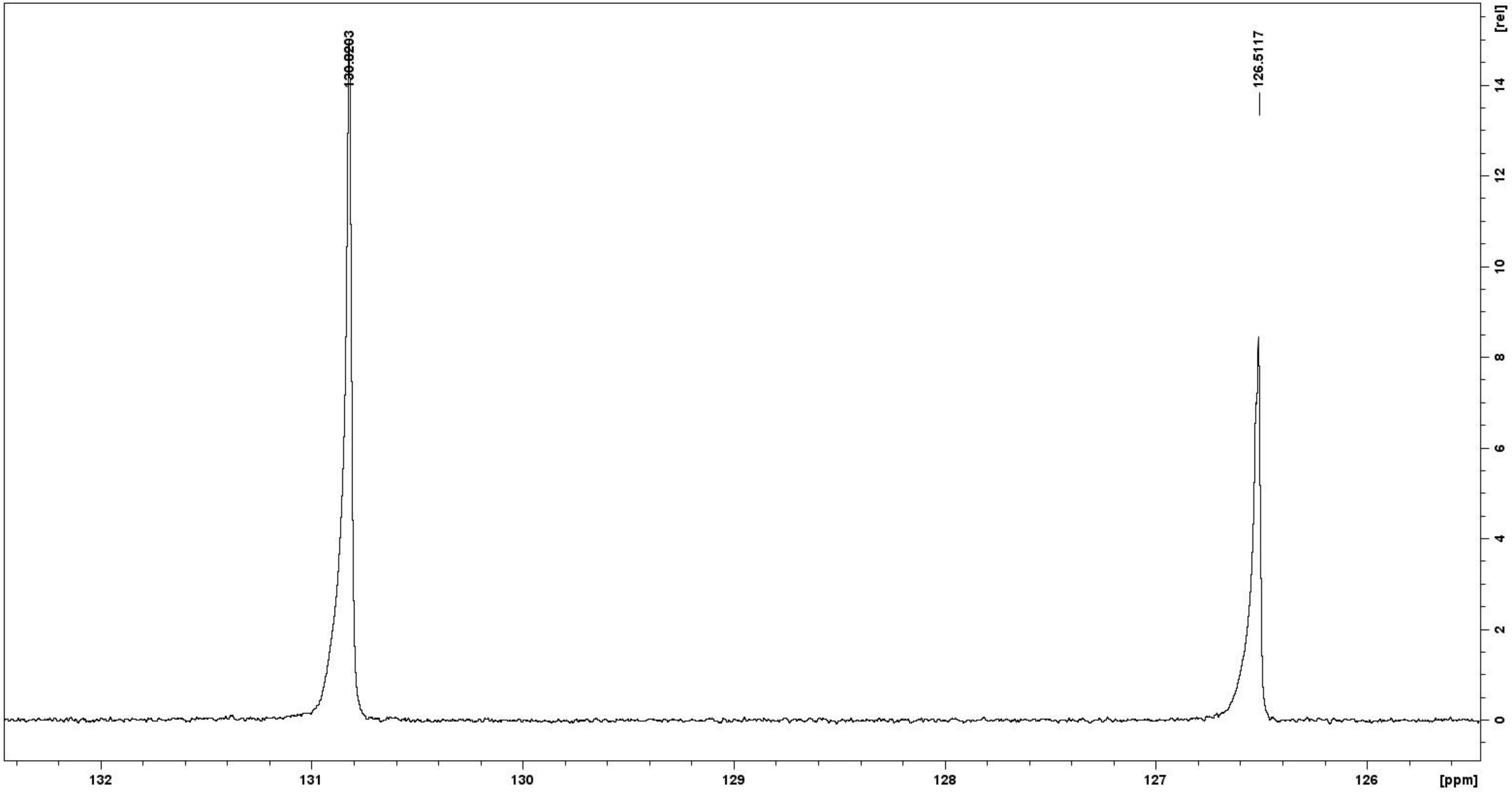

Expanded aromatic region of $^{13}C\{^{1}H\}$NMR spectrum of NDIC5

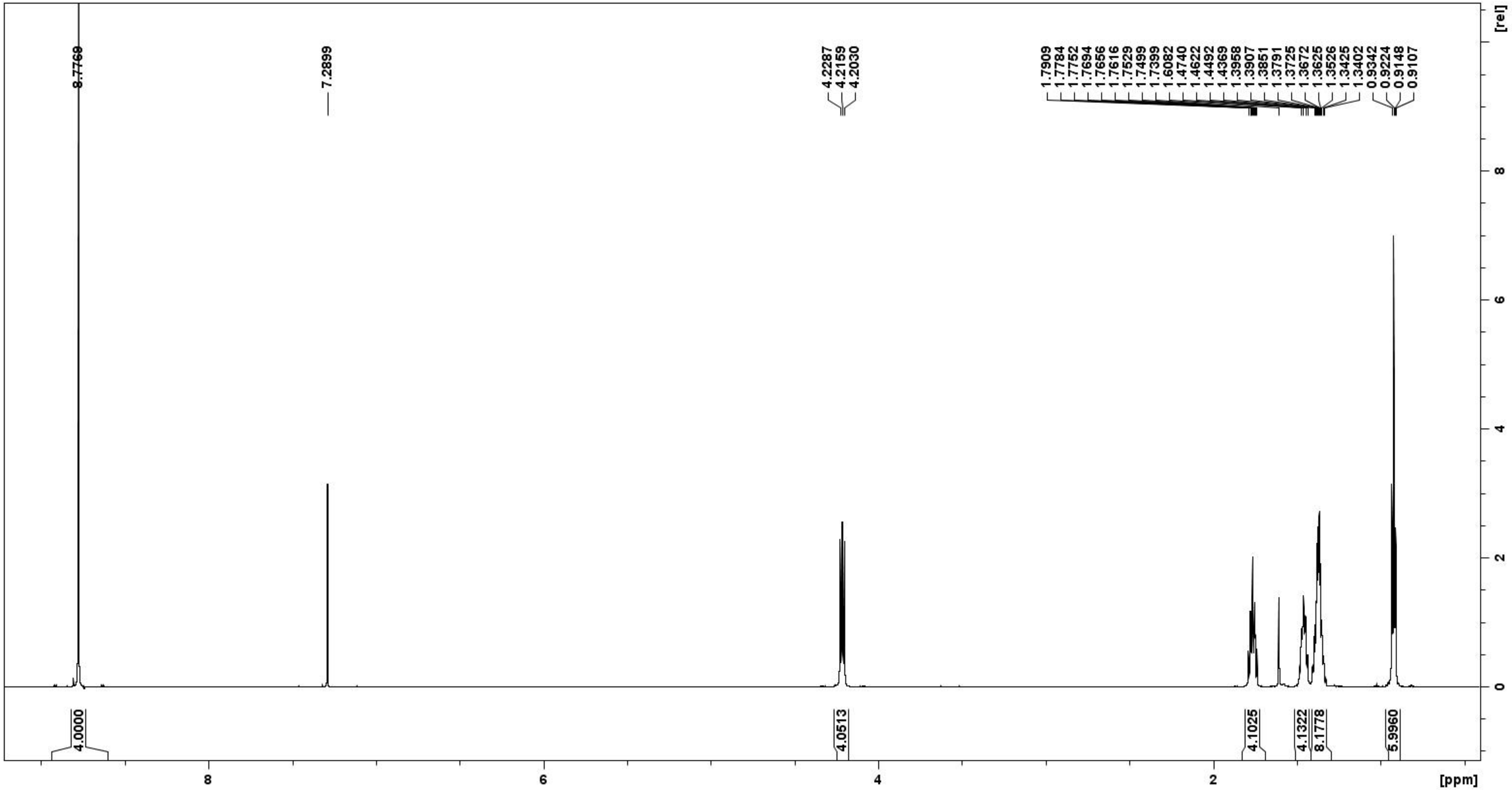

^{1}H NMR spectrum of NDIC6 ($CDCl_3$, 600MHz)

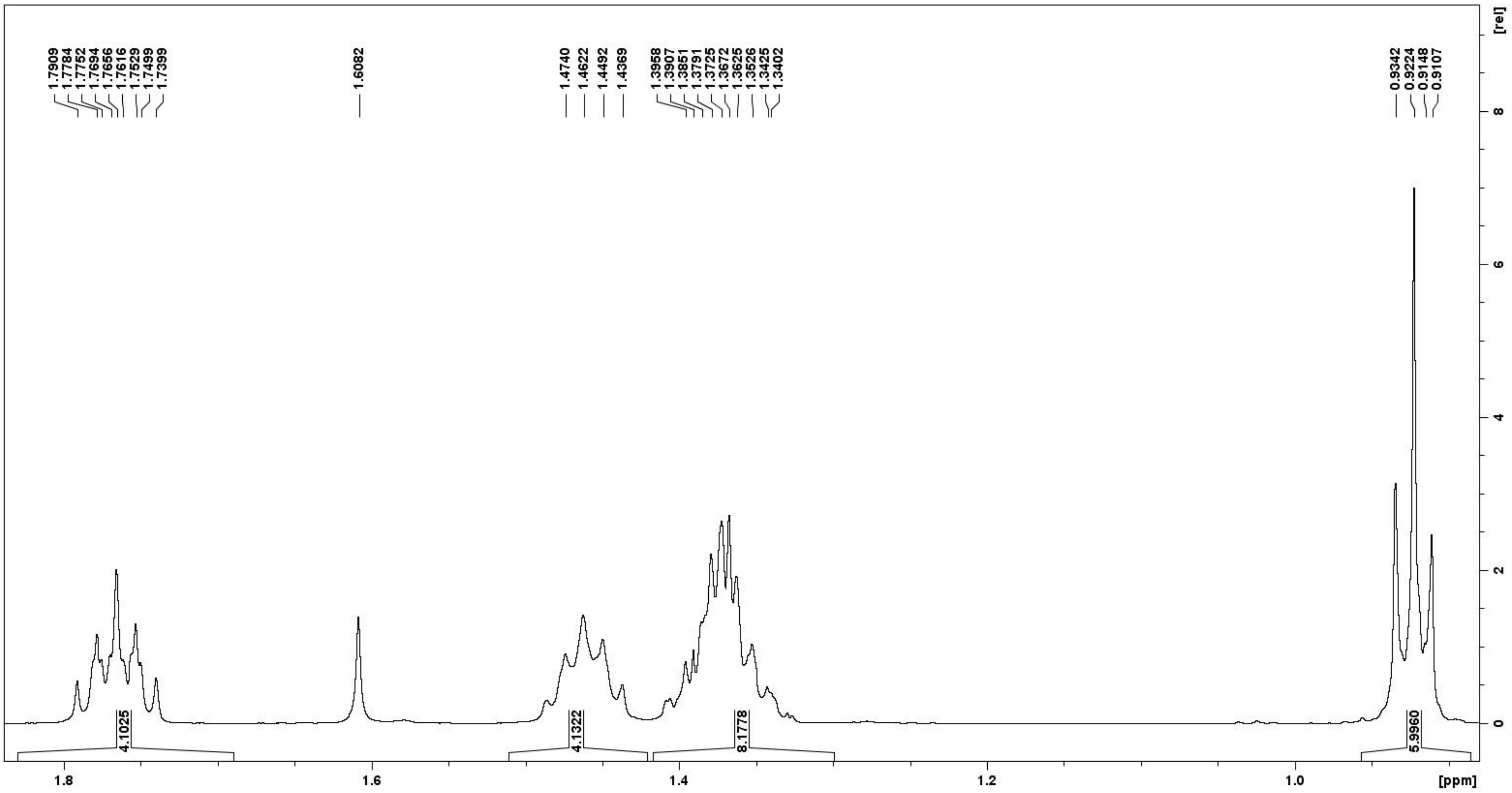

Expanded aliphatic region of ^{1}H NMR spectrum of NDIC6

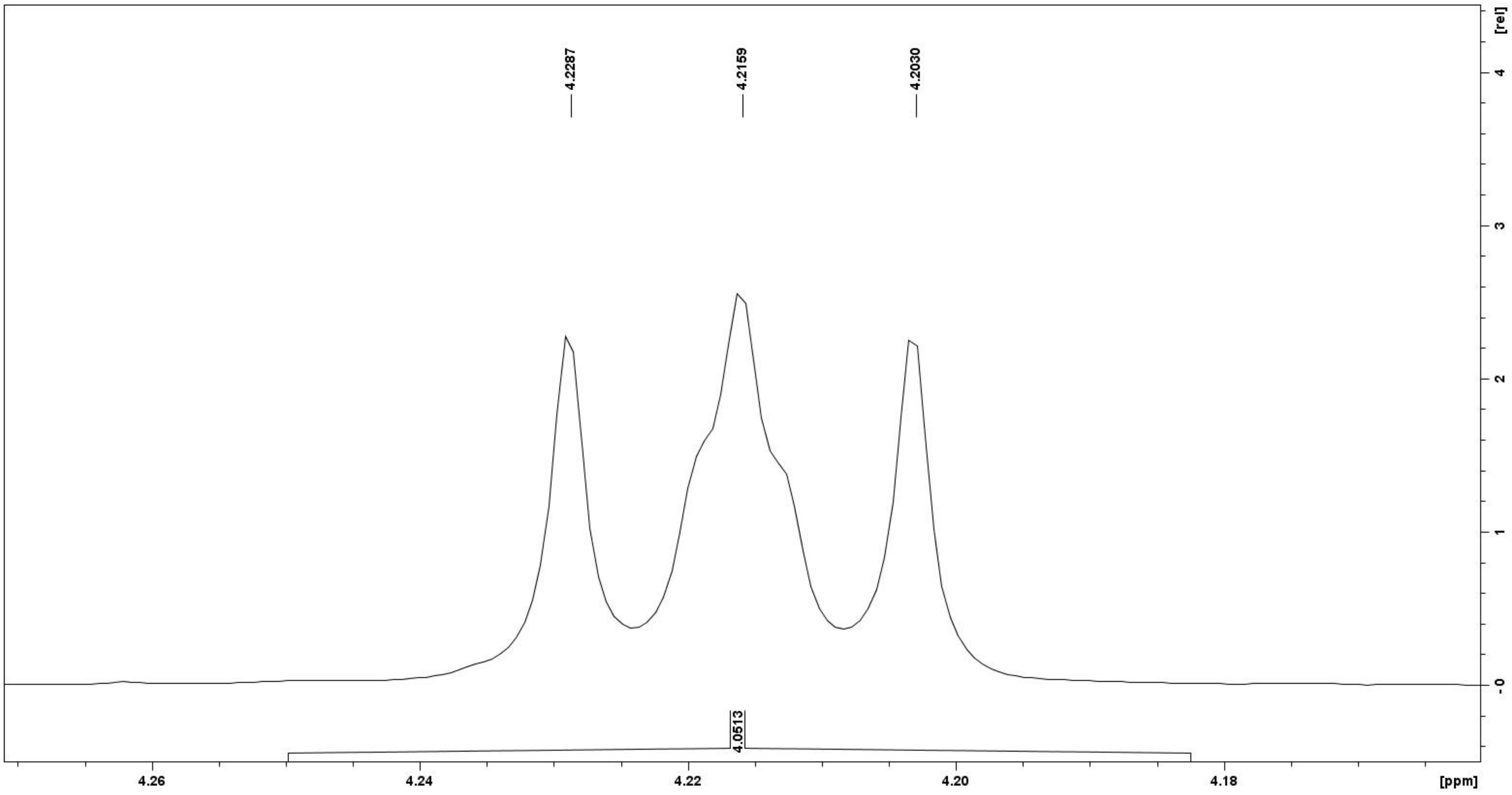

Expanded aliphatic region of ^{1}H NMR spectrum of NDIC6

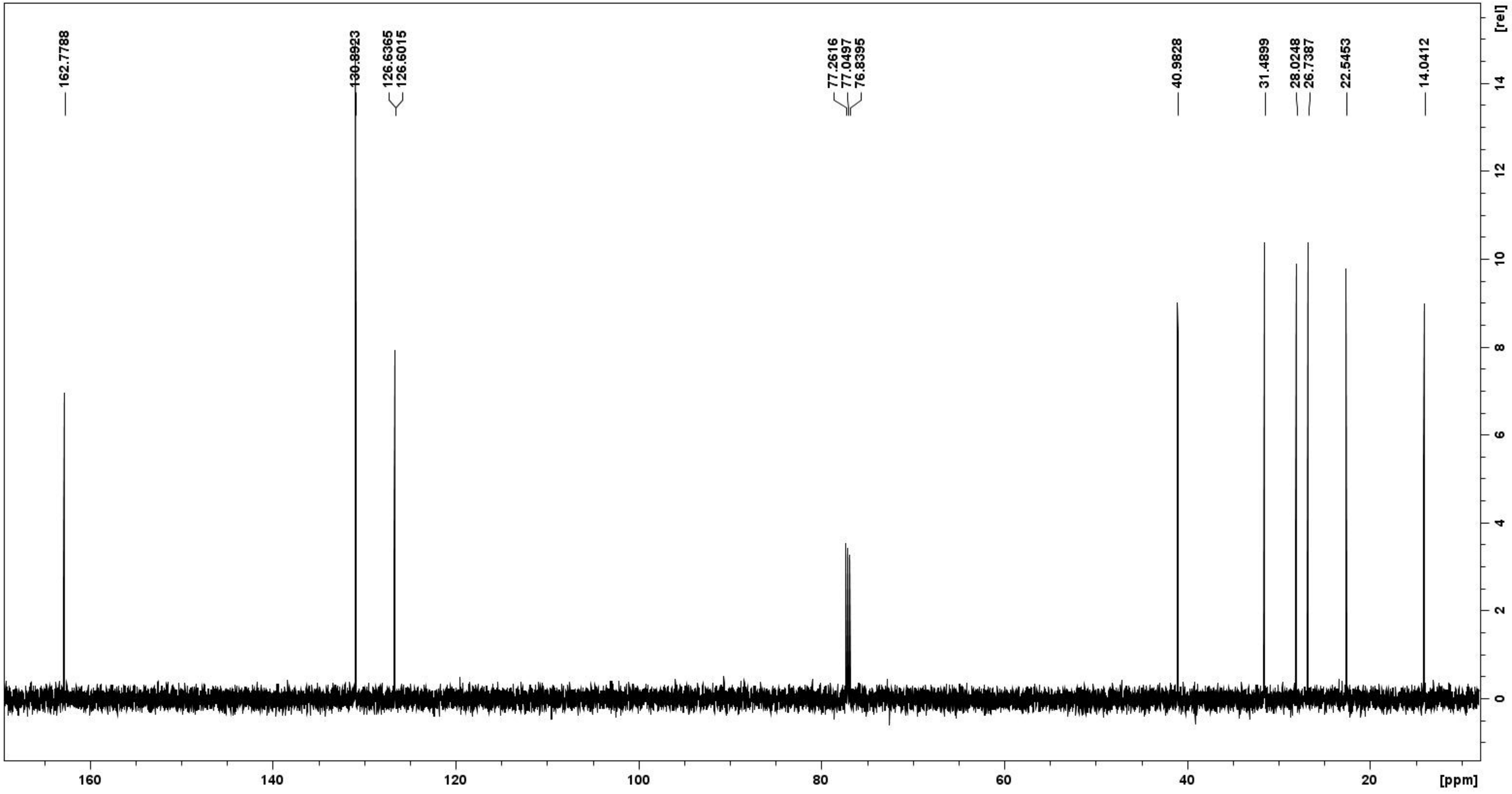

$^{13}C\{^{1}H\}$NMR spectrum of NDIC6 ($CDCl_3$, 151MHz)

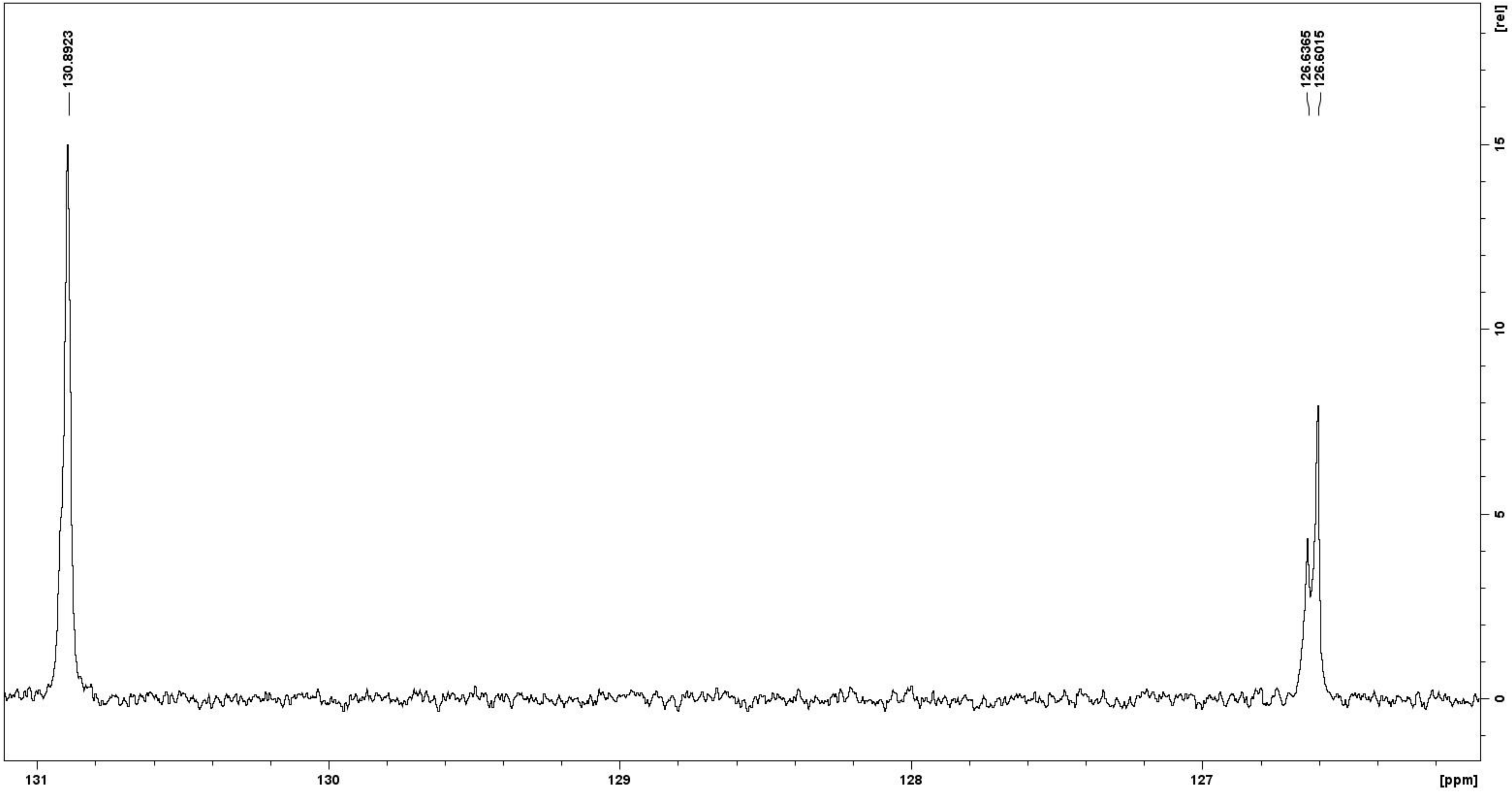

Expanded aromatic region of $^{13}C\{^1H\}$NMR spectrum of NDIC6

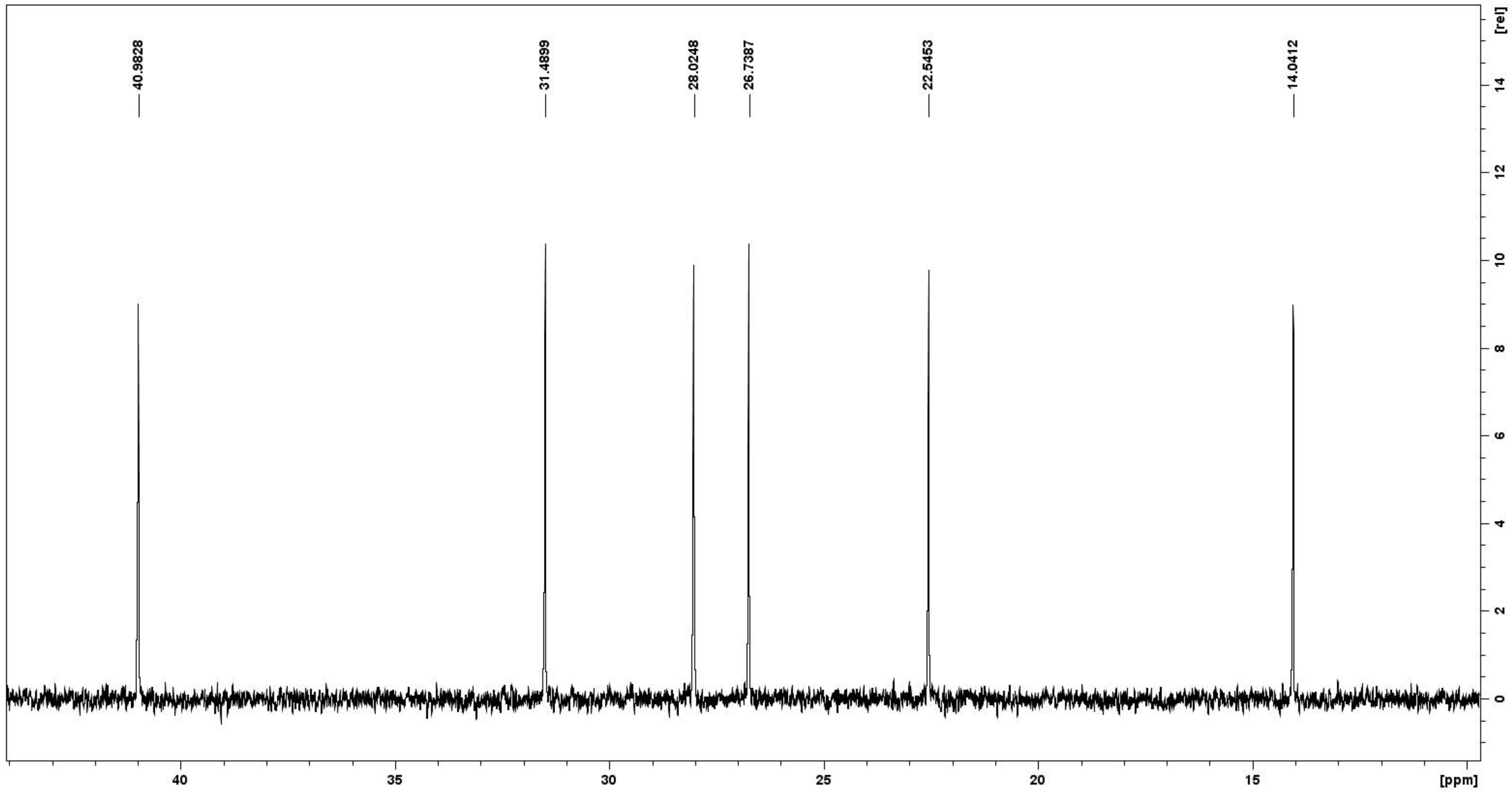

Expanded aliphatic region of $^{13}C\{^{1}H\}$NMR spectrum of NDIC6

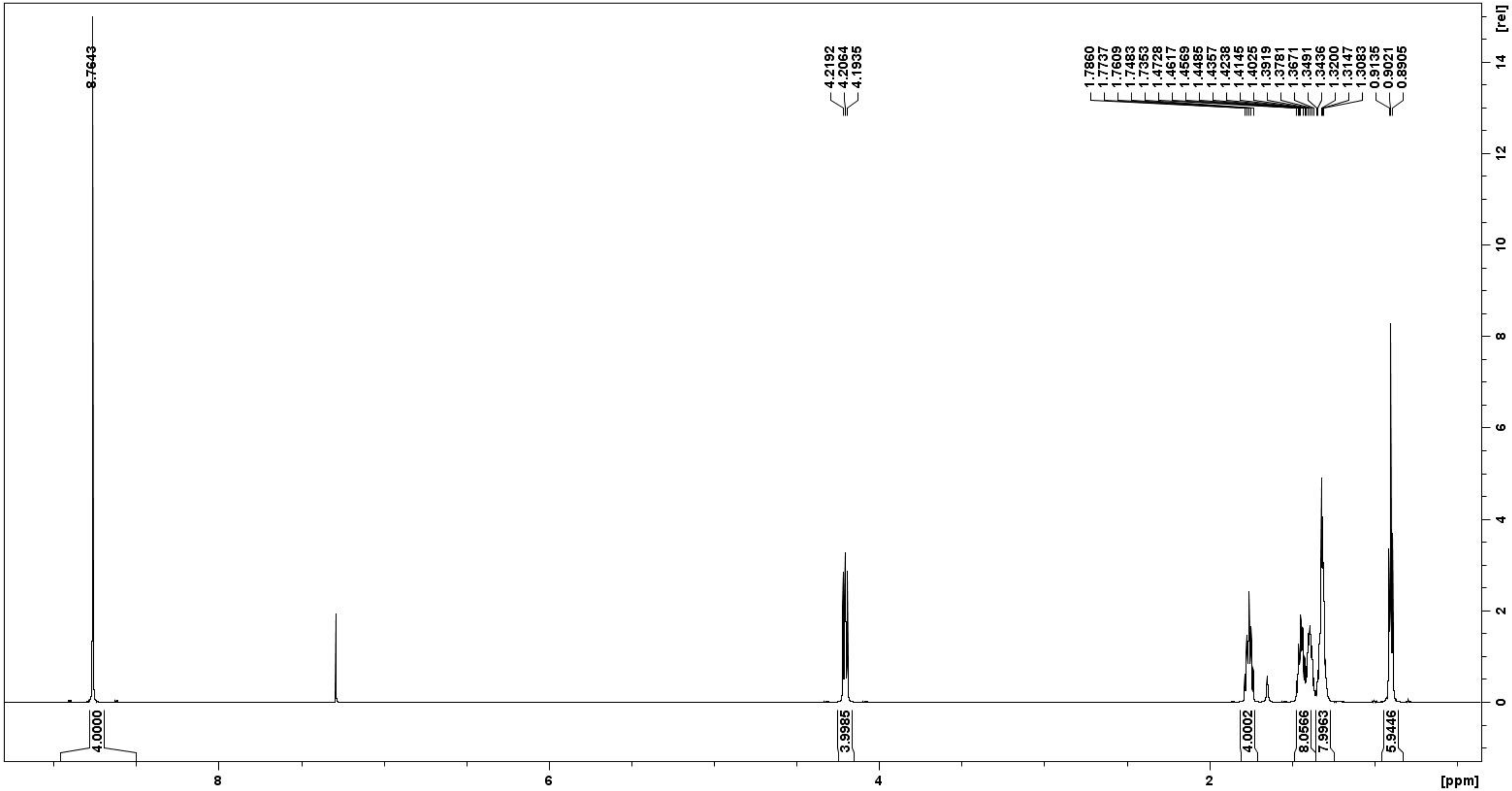

^{1}H NMR spectrum of NDIC7 ($CDCl_3$, 600MHz)

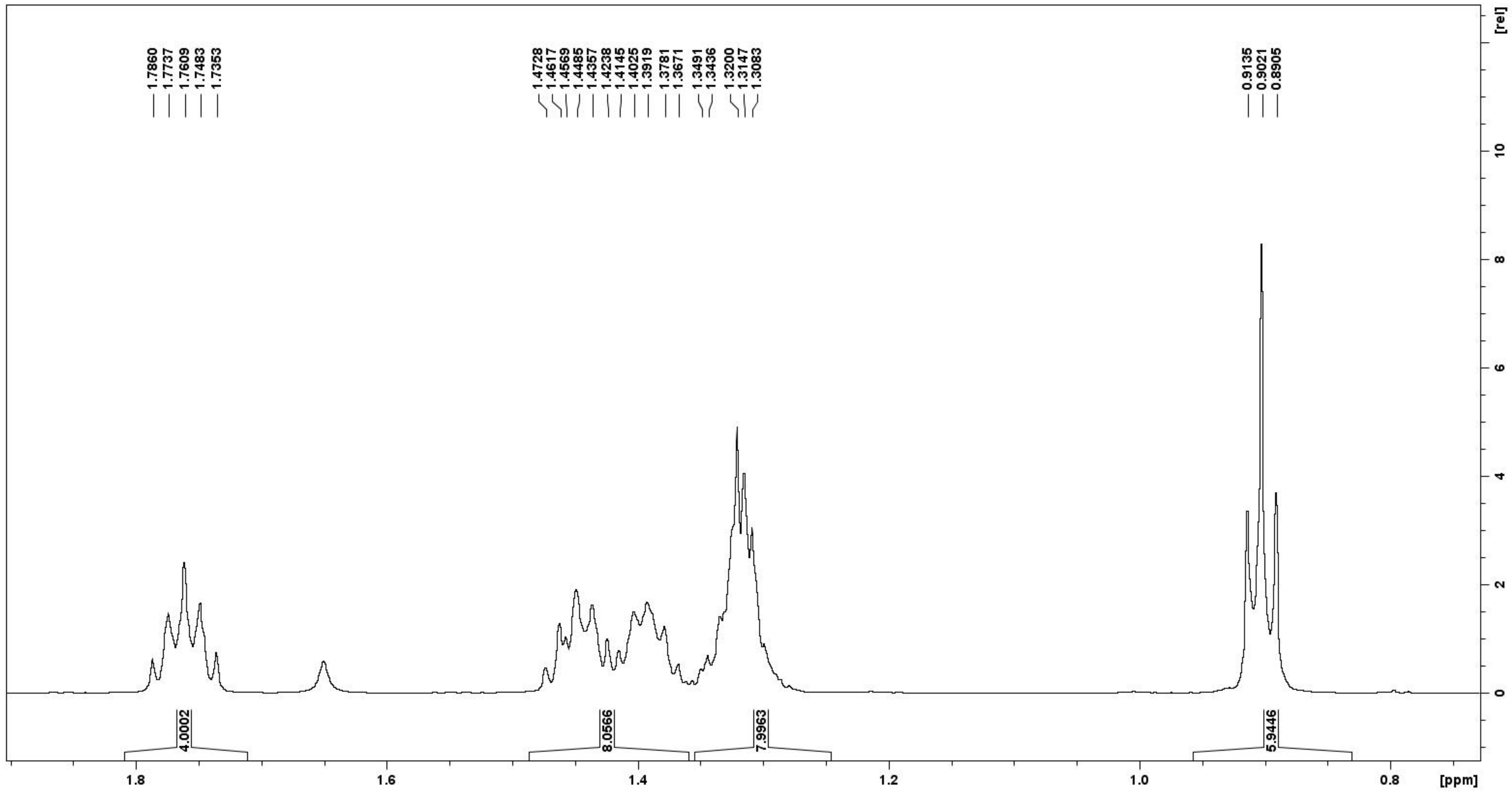

Expanded aliphatic region of ^{1}H NMR spectrum of NDIC7

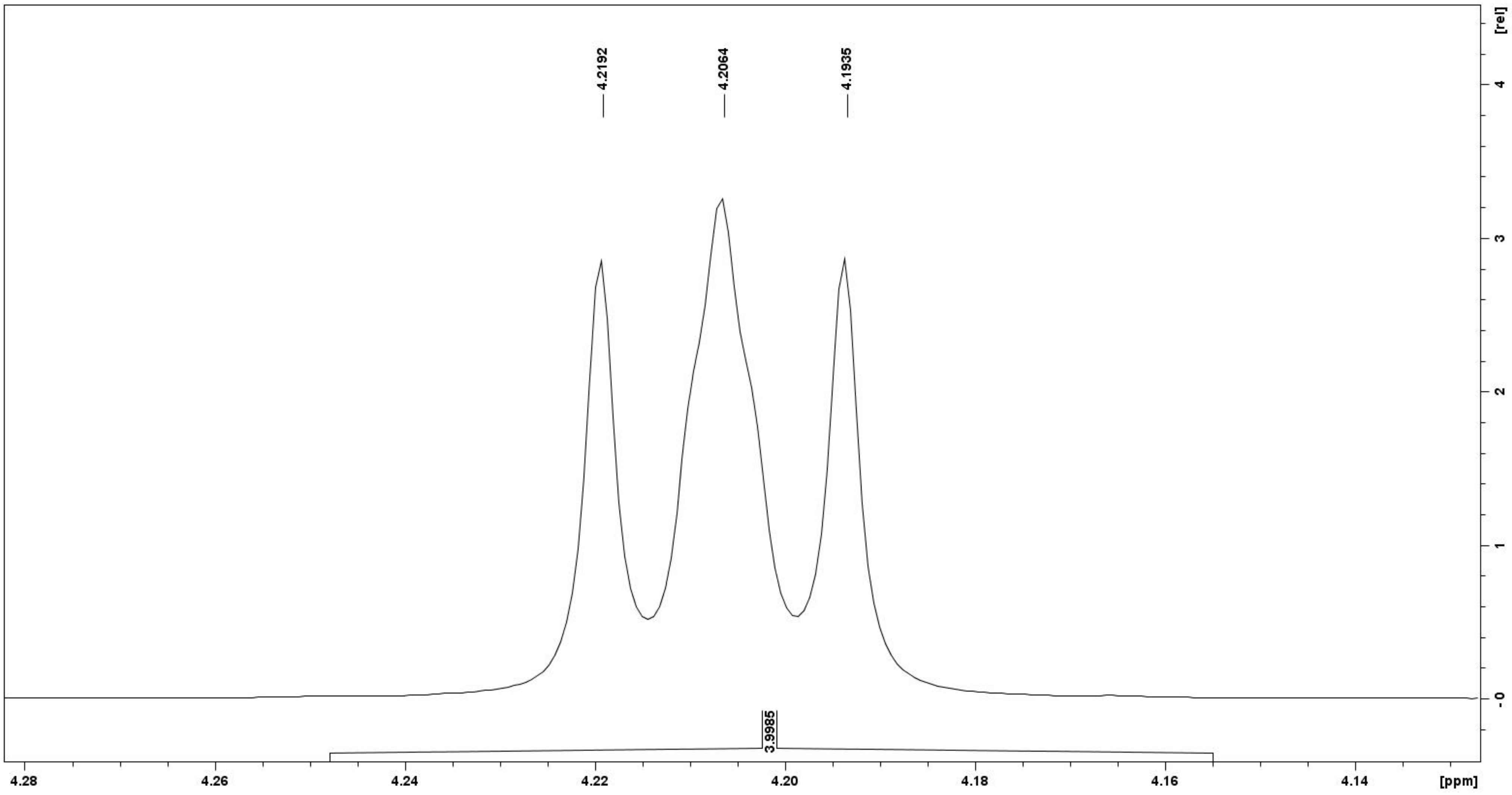

Expanded aliphatic region of ^{1}H NMR spectrum of NDIC7

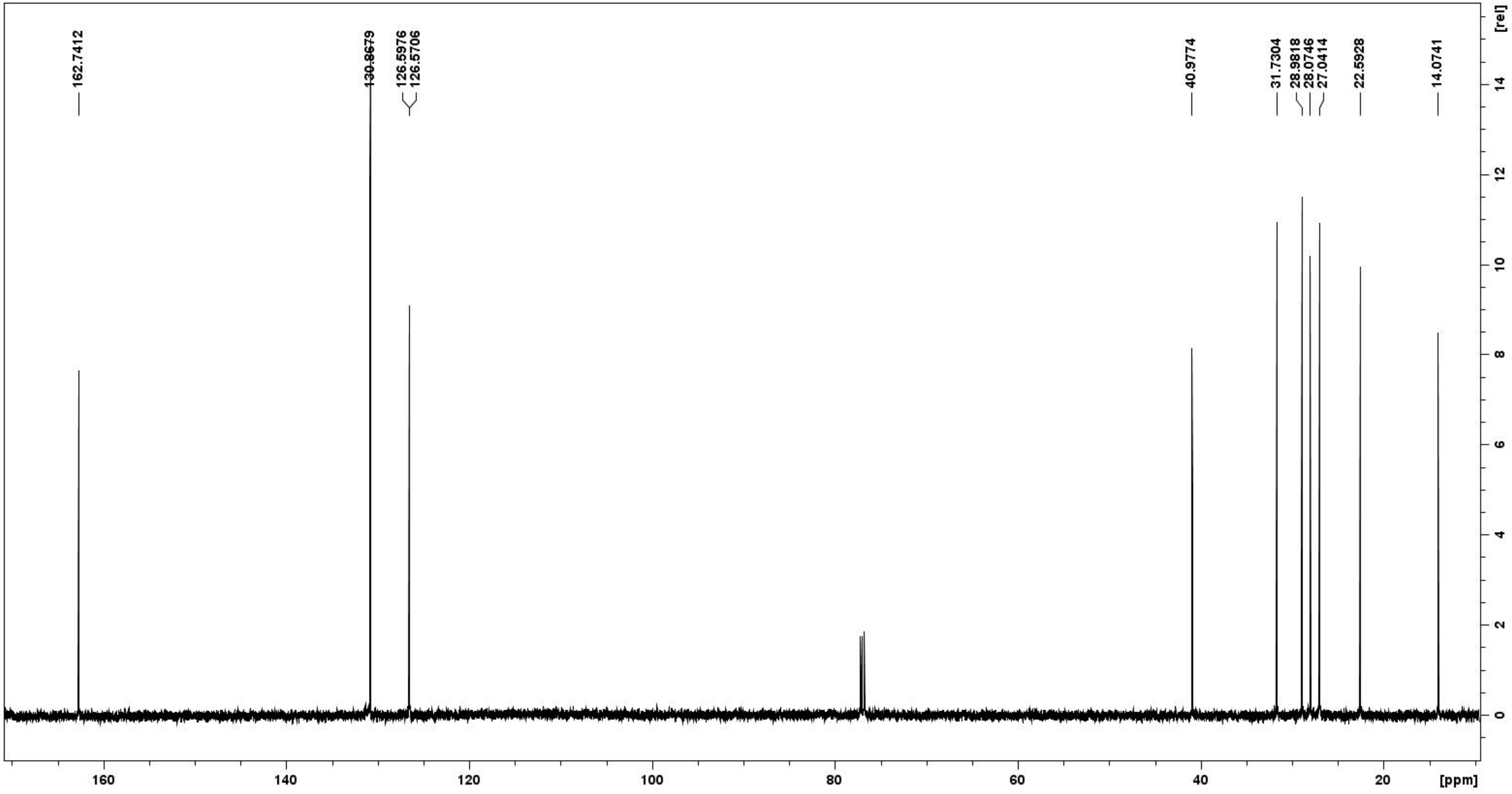

$^{13}C\{^{1}H\}$NMR spectrum of NDIC7 ($CDCl_3$, 151MHz)

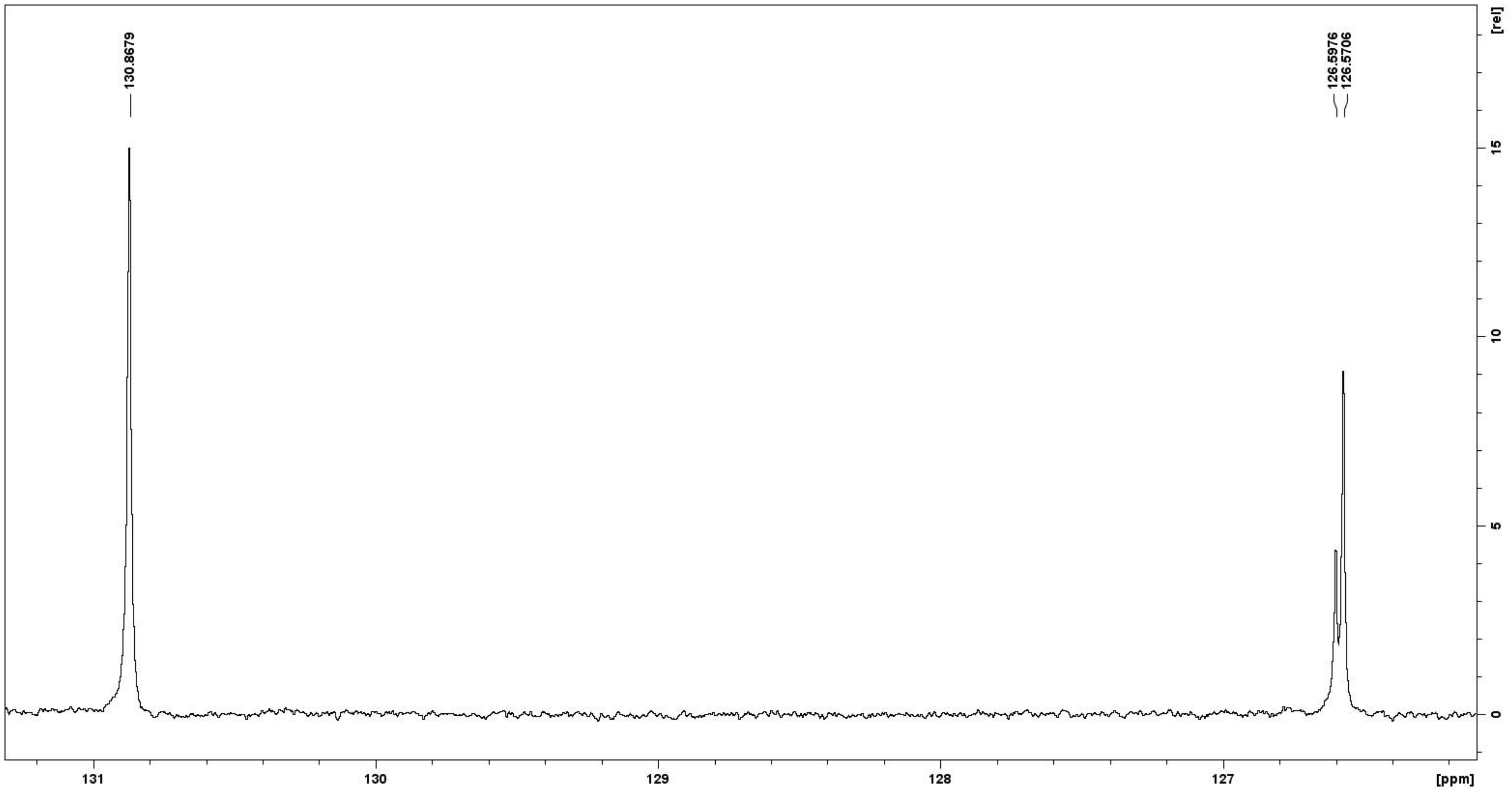

Expanded aromatic region of $^{13}C\{^{1}H\}$NMR spectrum of NDIC7

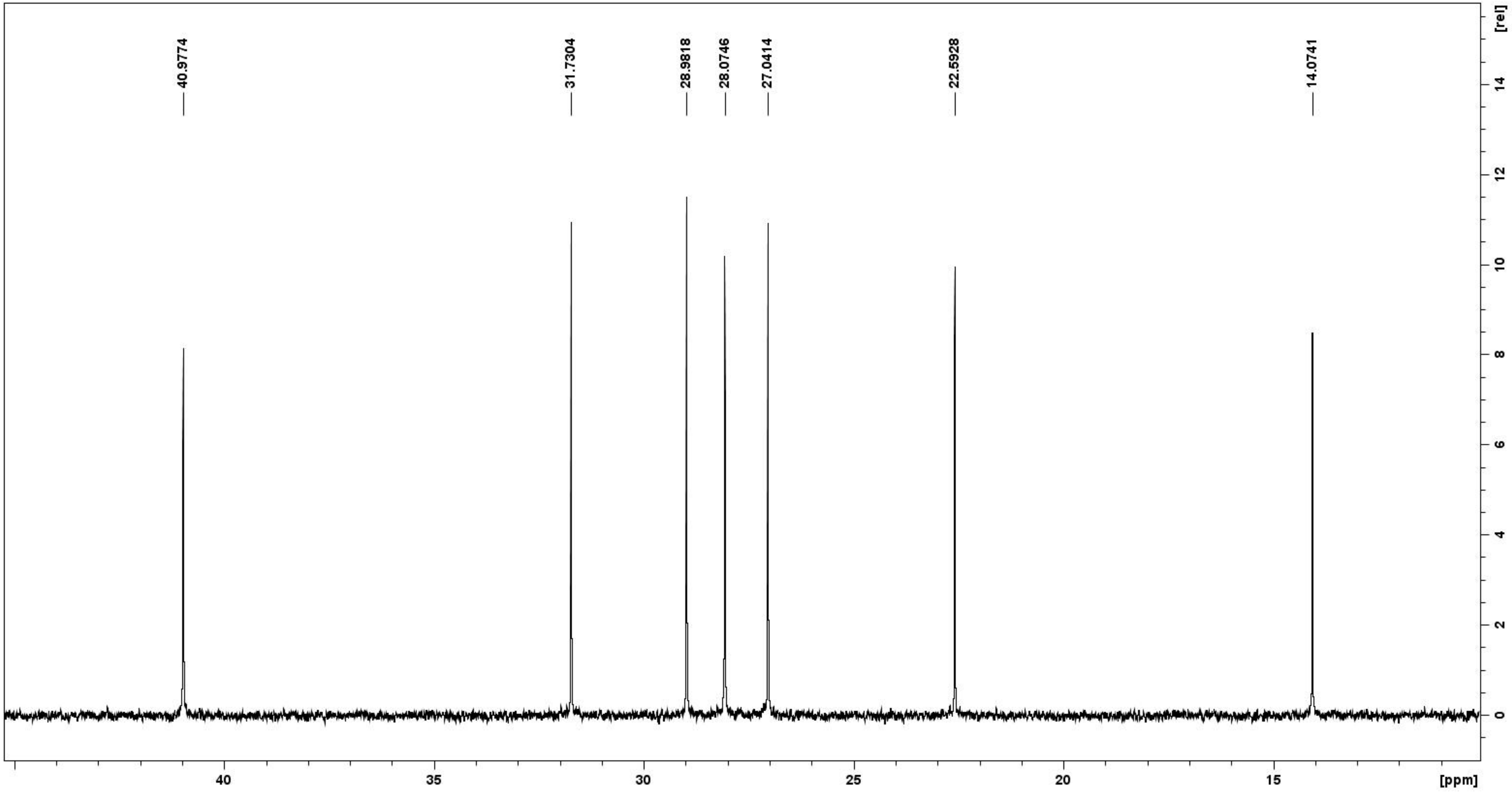

Expanded aliphatic region of $^{13}C\{^1H\}$NMR spectrum of NDIC7

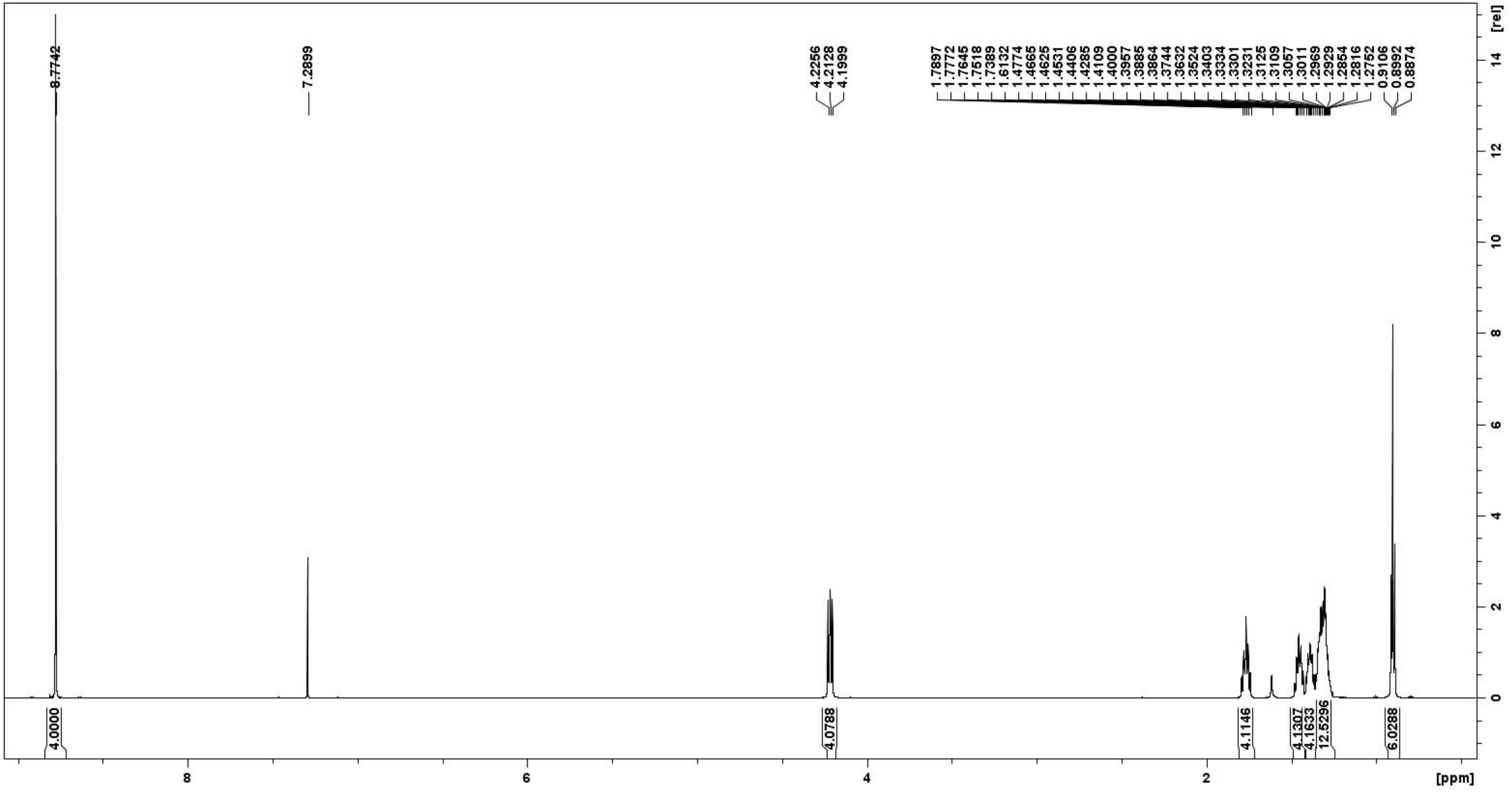

^{1}H NMR spectrum of NDIC8 ($CDCl_3$, 600MHz)

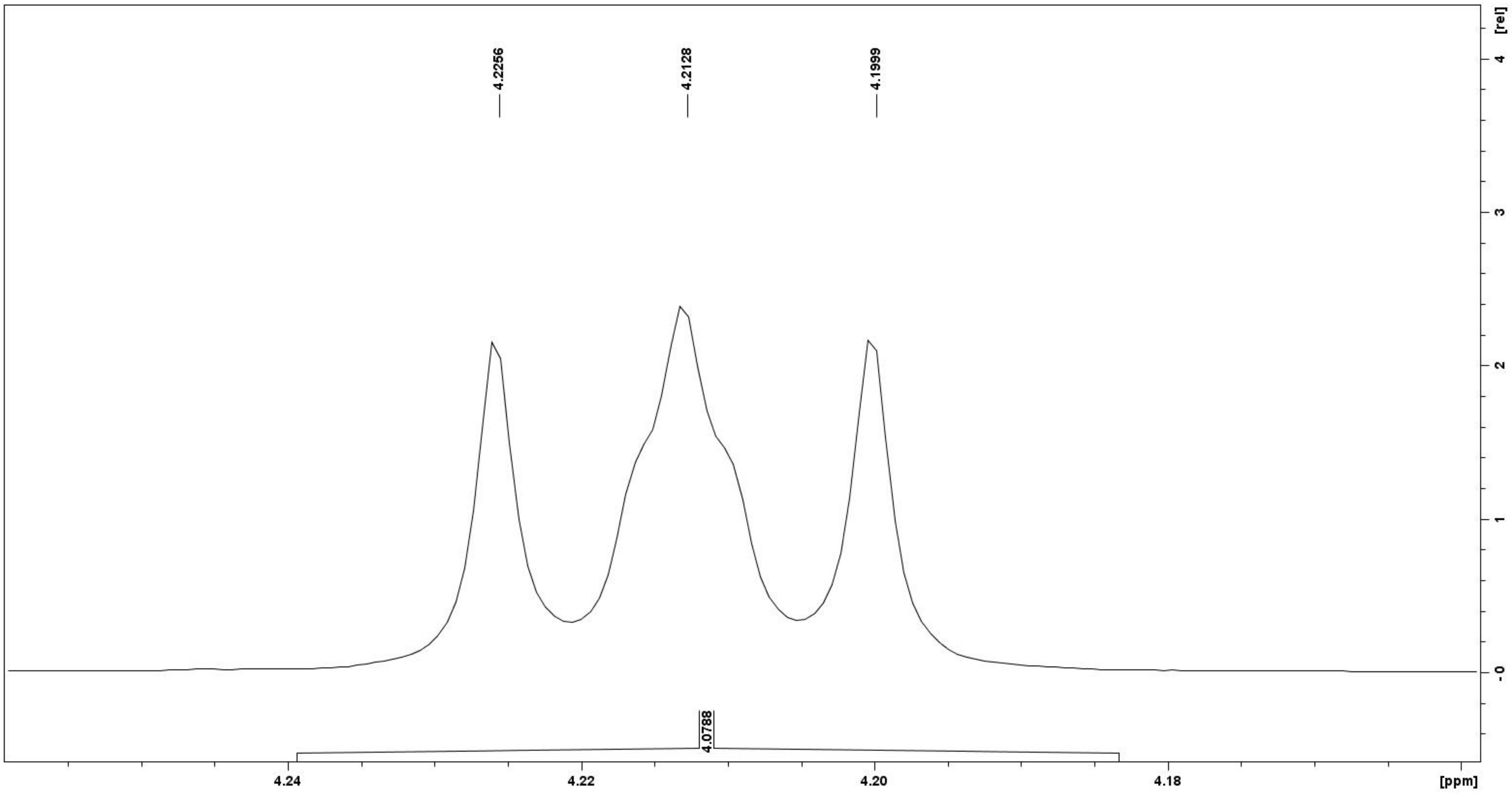

Expanded aliphatic region of ^{1}H NMR spectrum of NDIC8

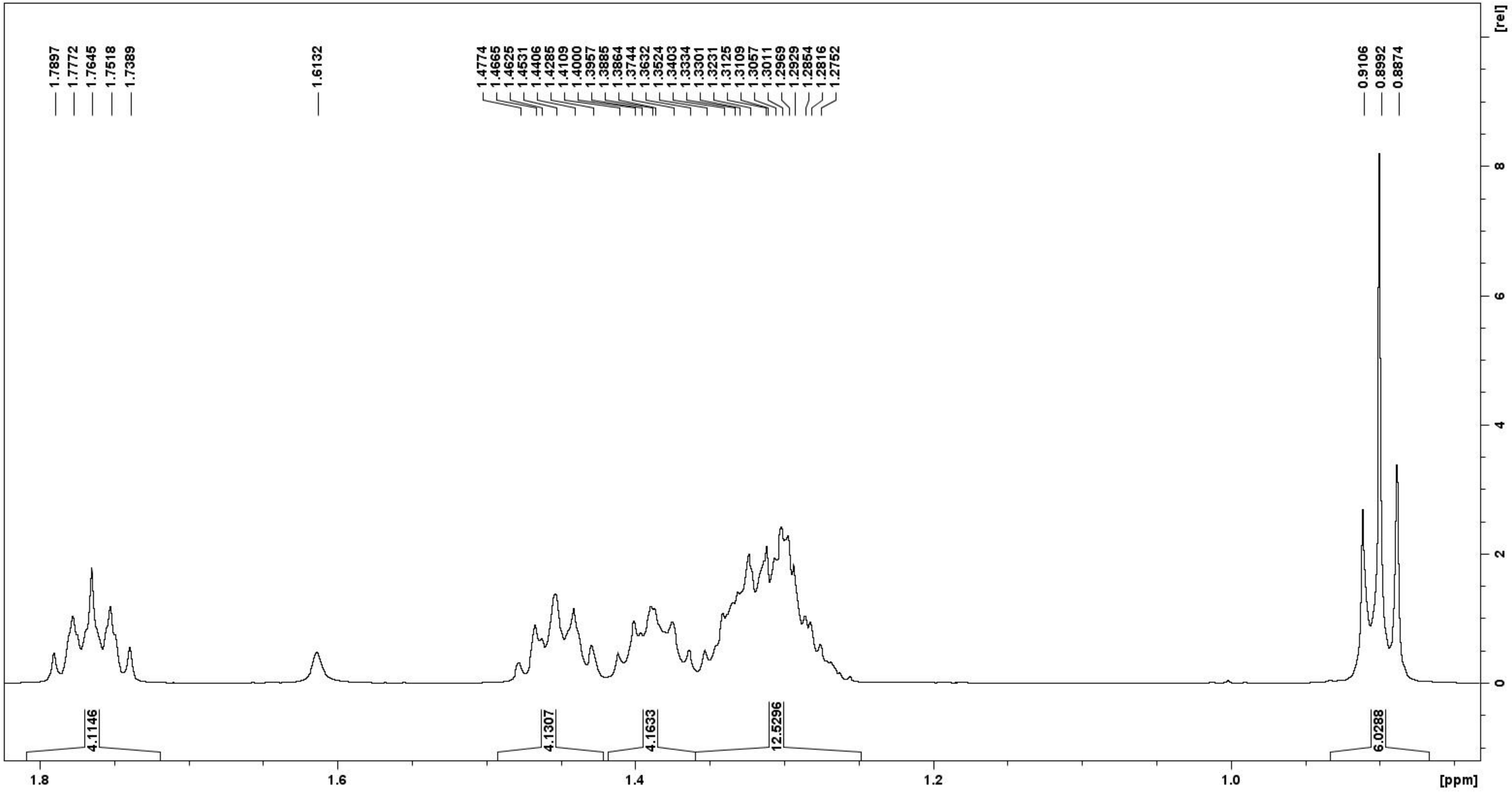

Expanded aliphatic region of ^{1}H NMR spectrum of NDIC8

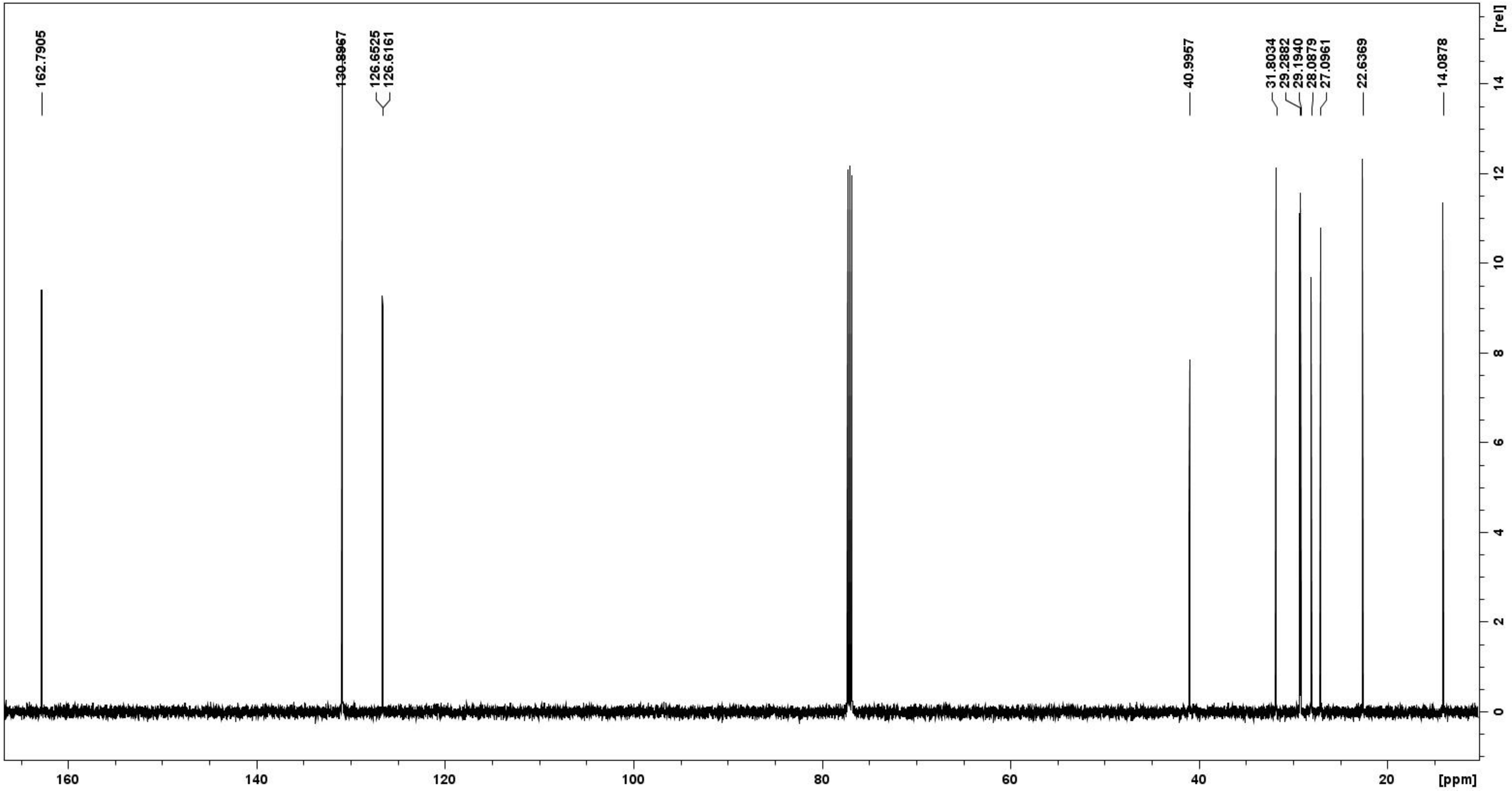

$^{13}C\{^{1}H\}$NMR spectrum of NDIC8 ($CDCl_3$, 151MHz)

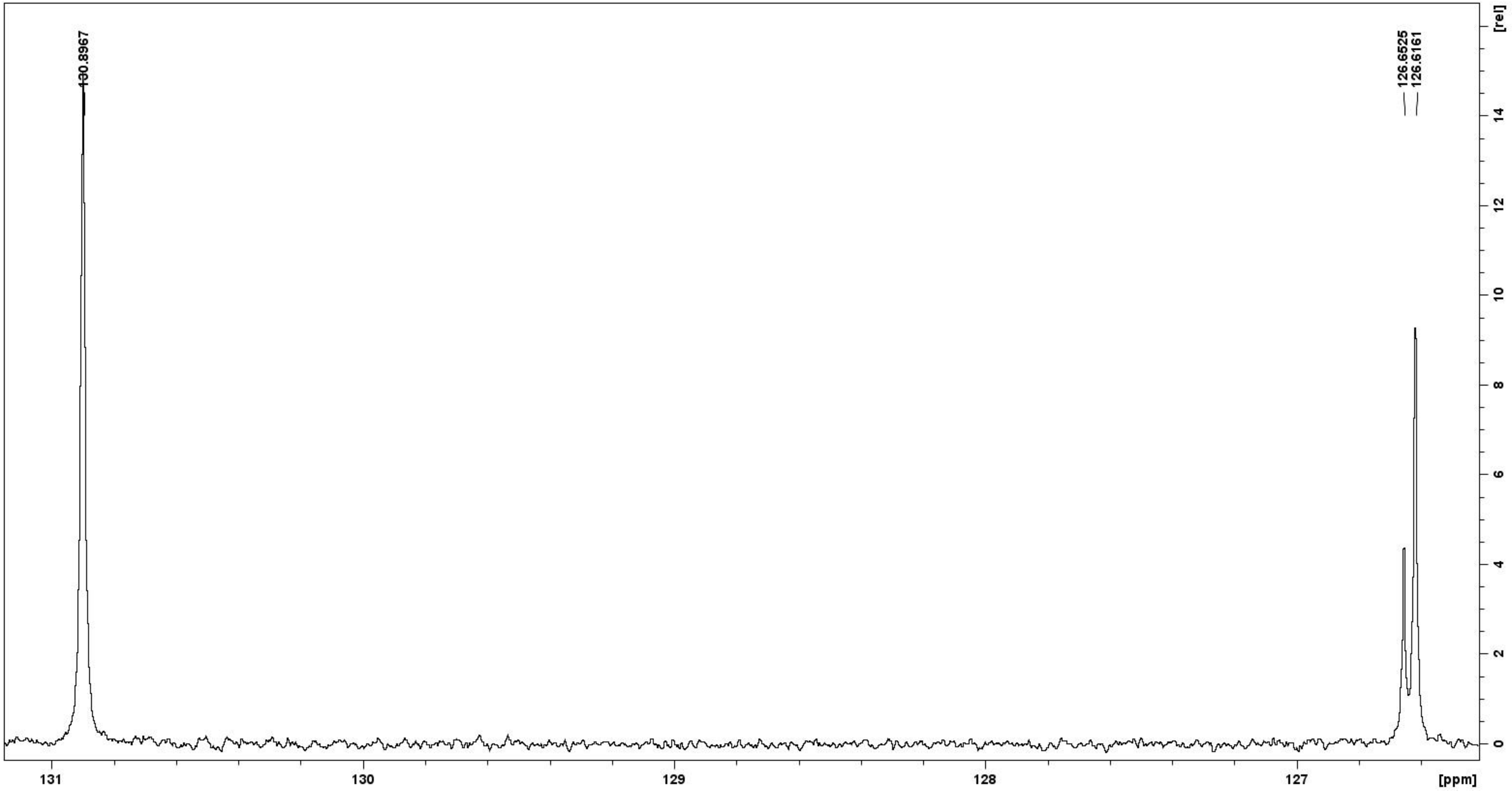

Expanded aromatic region of $^{13}C\{^{1}H\}$NMR spectrum of NDIC8

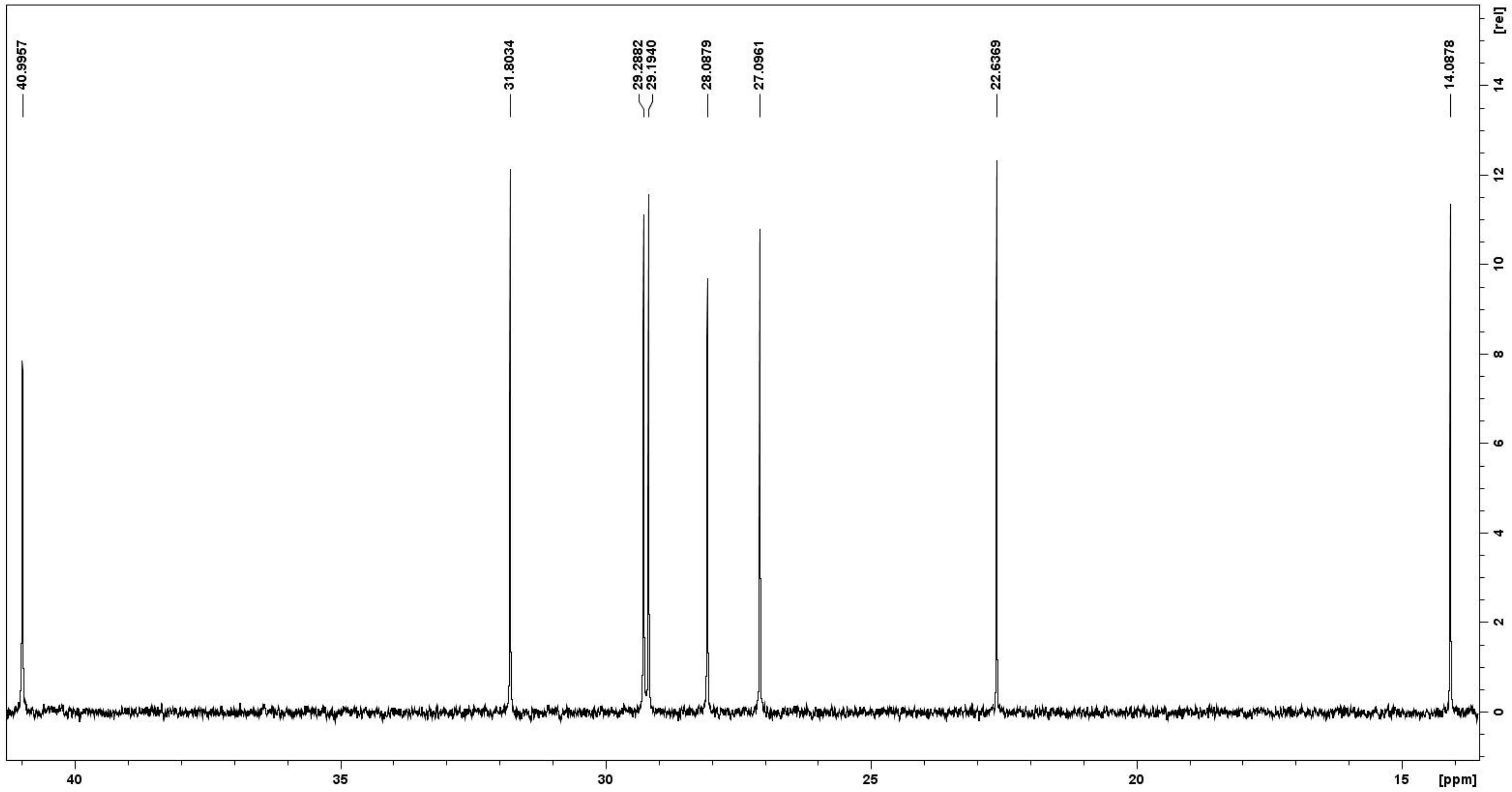

Expanded aliphatic region of $^{13}C\{^{1}H\}$NMR spectrum of NDIC8

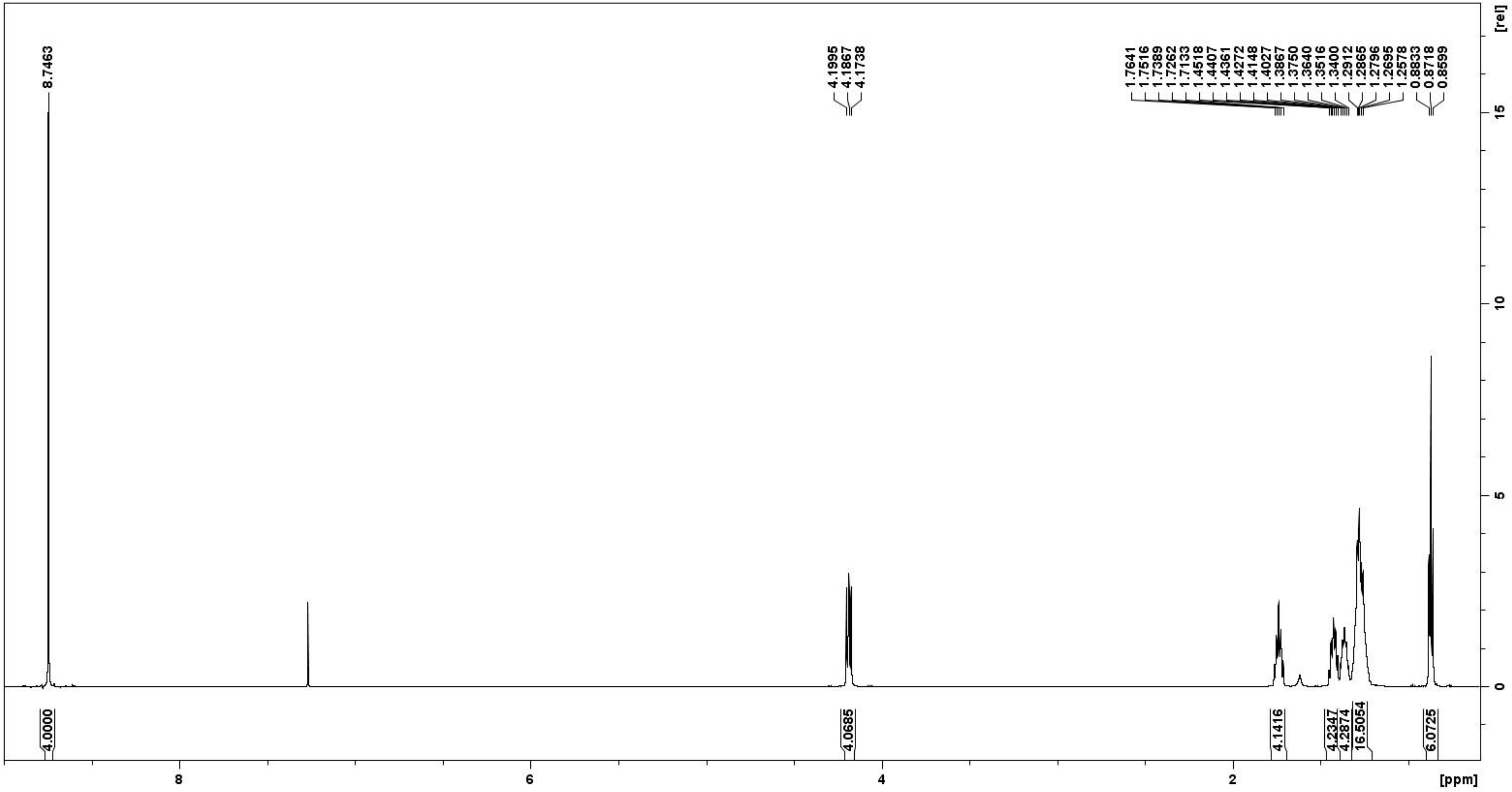

^{1}H NMR spectrum of NDIC9 ($CDCl_3$, 600MHz)

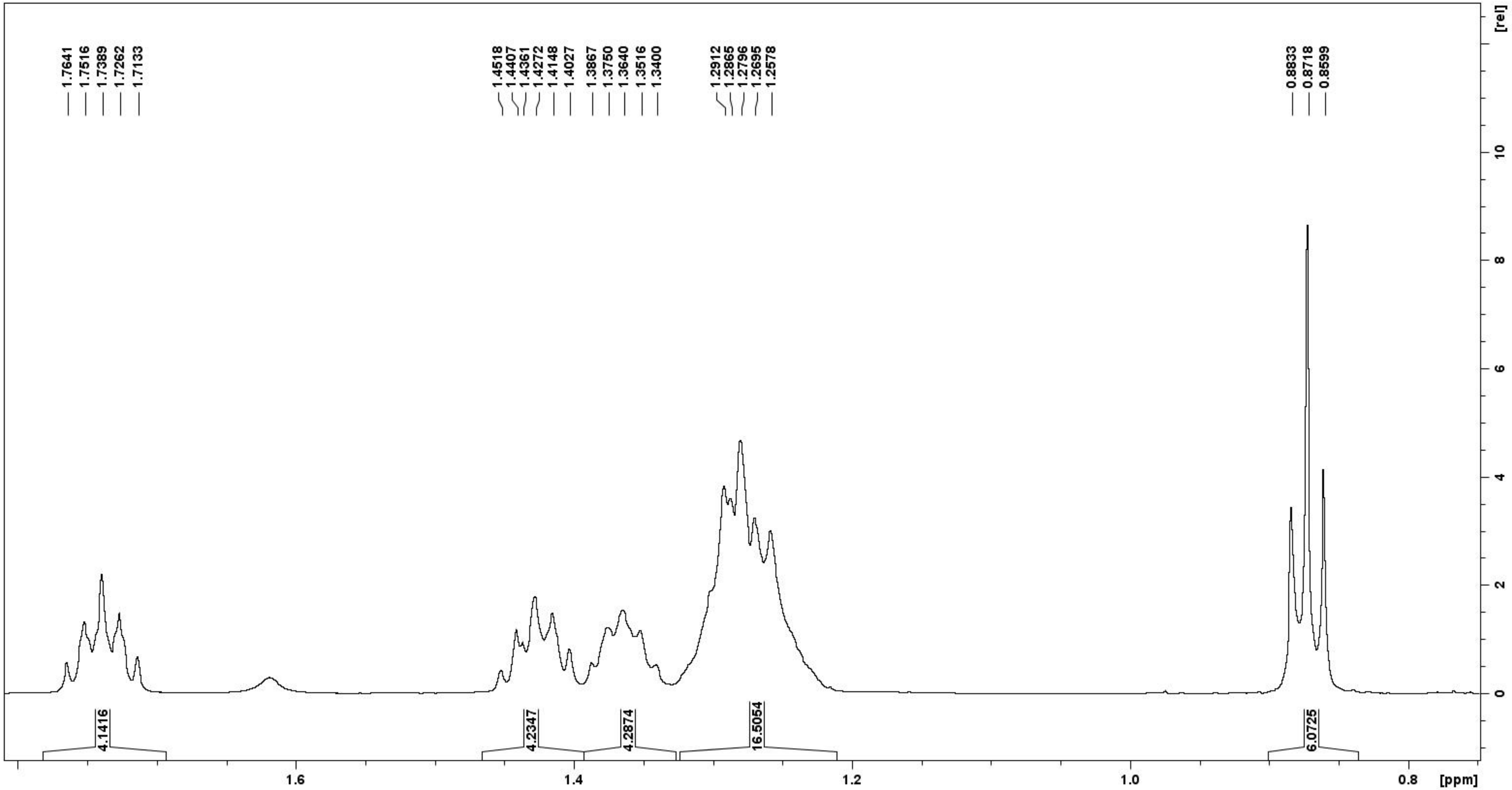

Expanded aliphatic region of ^{1}H NMR spectrum of NDIC9

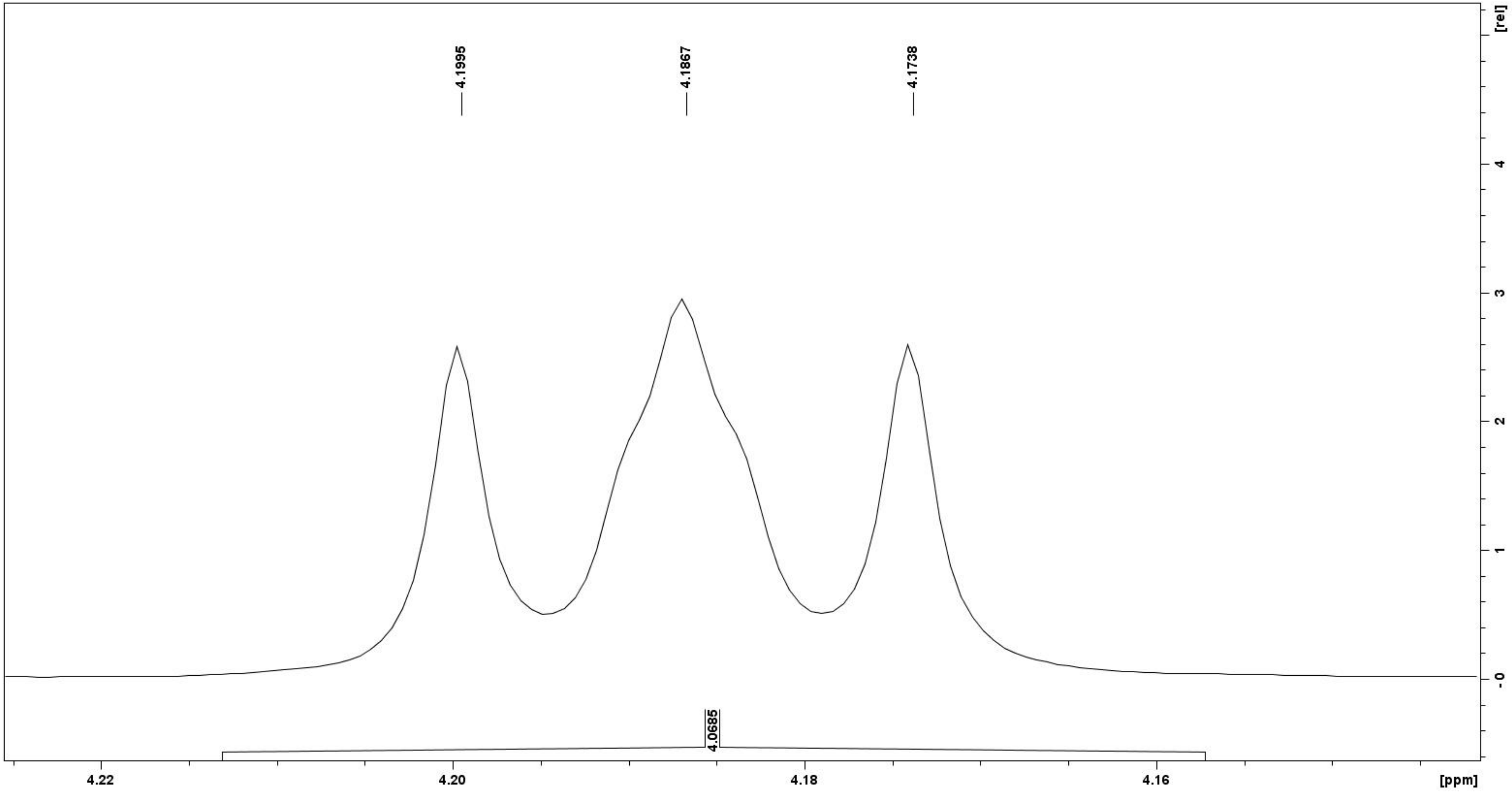

Expanded aliphatic region of ^{1}H NMR spectrum of NDIC9

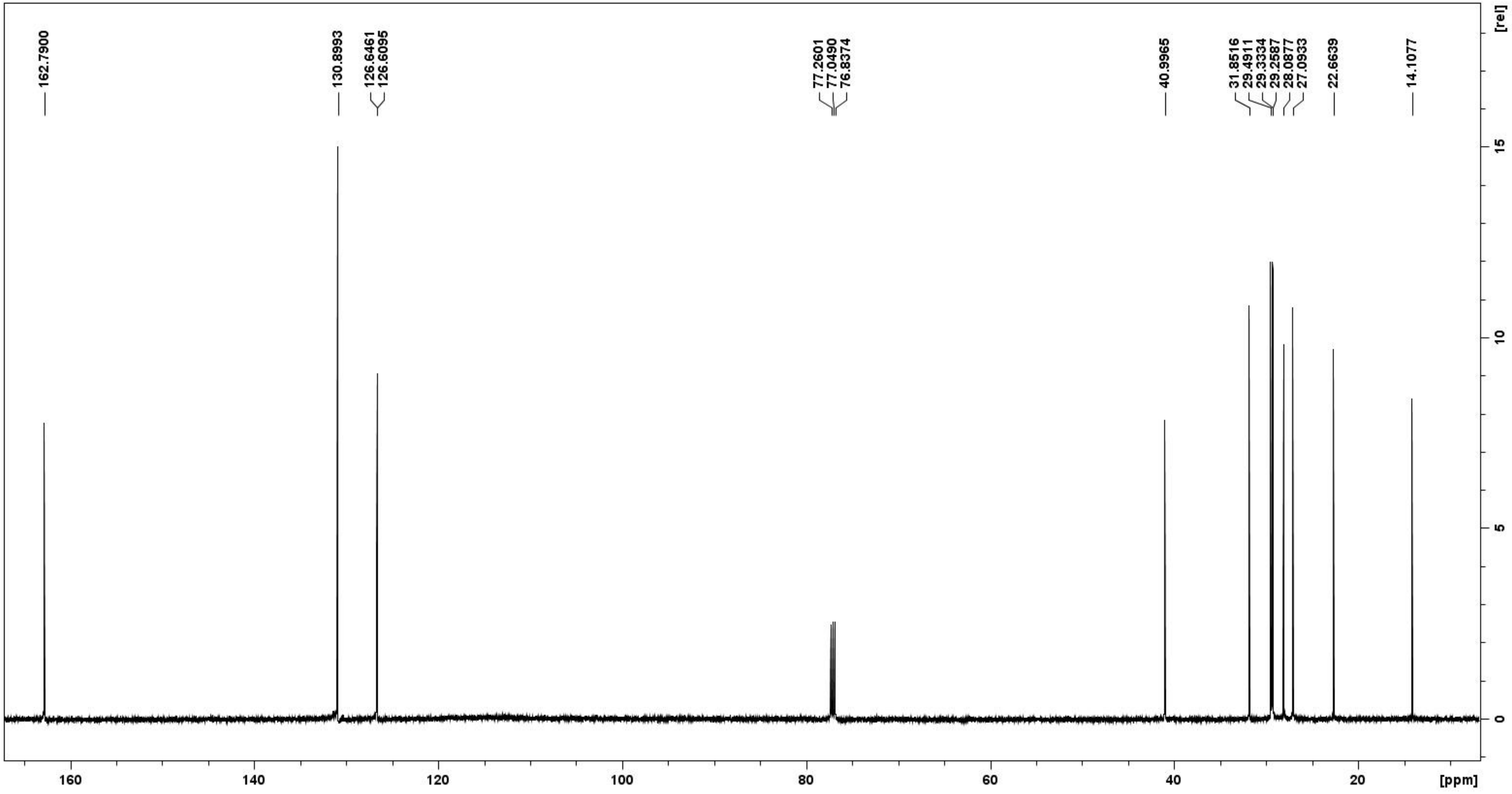

$^{13}C\{^{1}H\}$NMR spectrum of NDIC9($CDCl_3$, 151MHz)

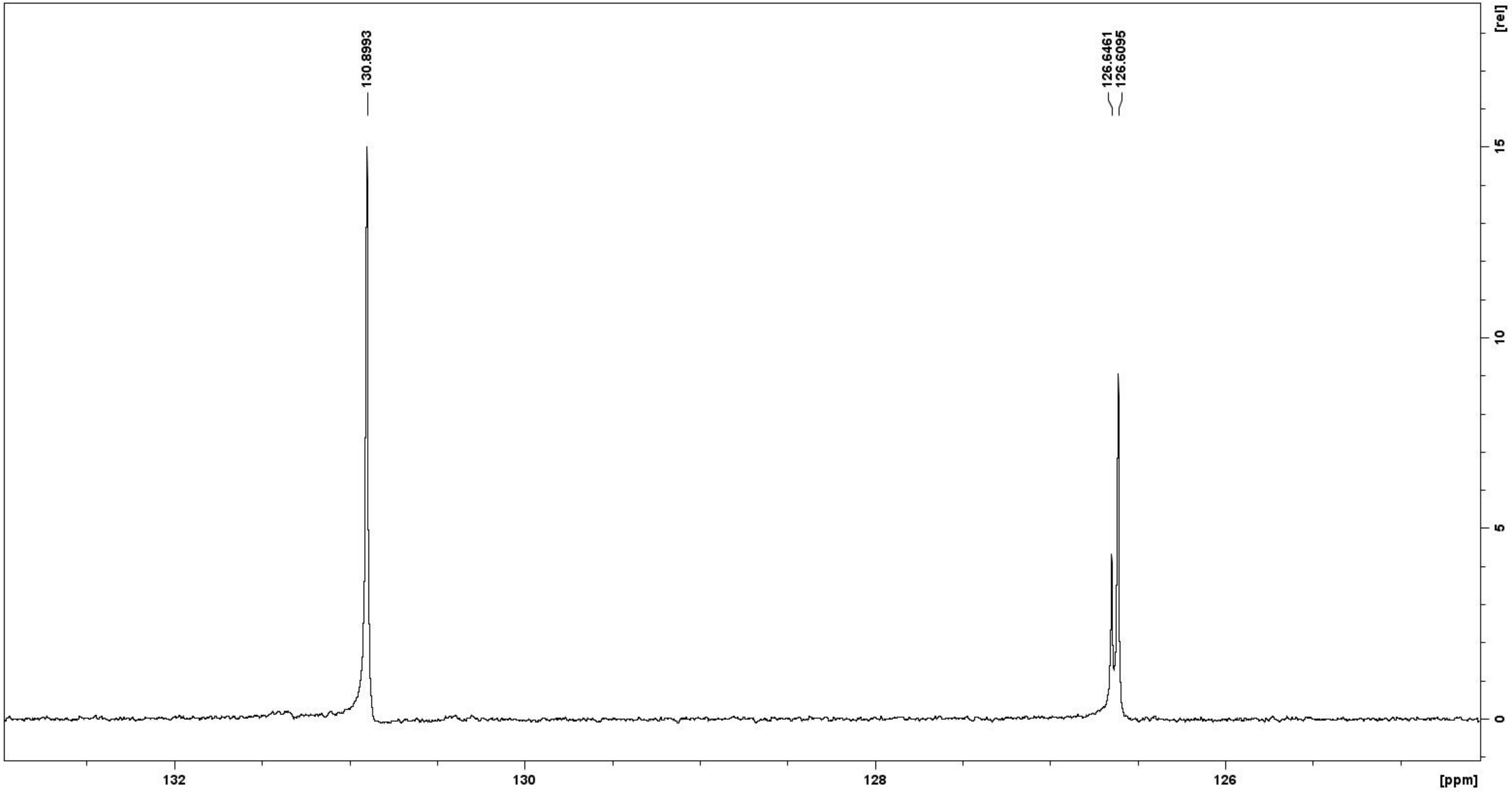

Expanded aromatic region of $^{13}C\{^{1}H\}$NMR spectrum of NDIC9

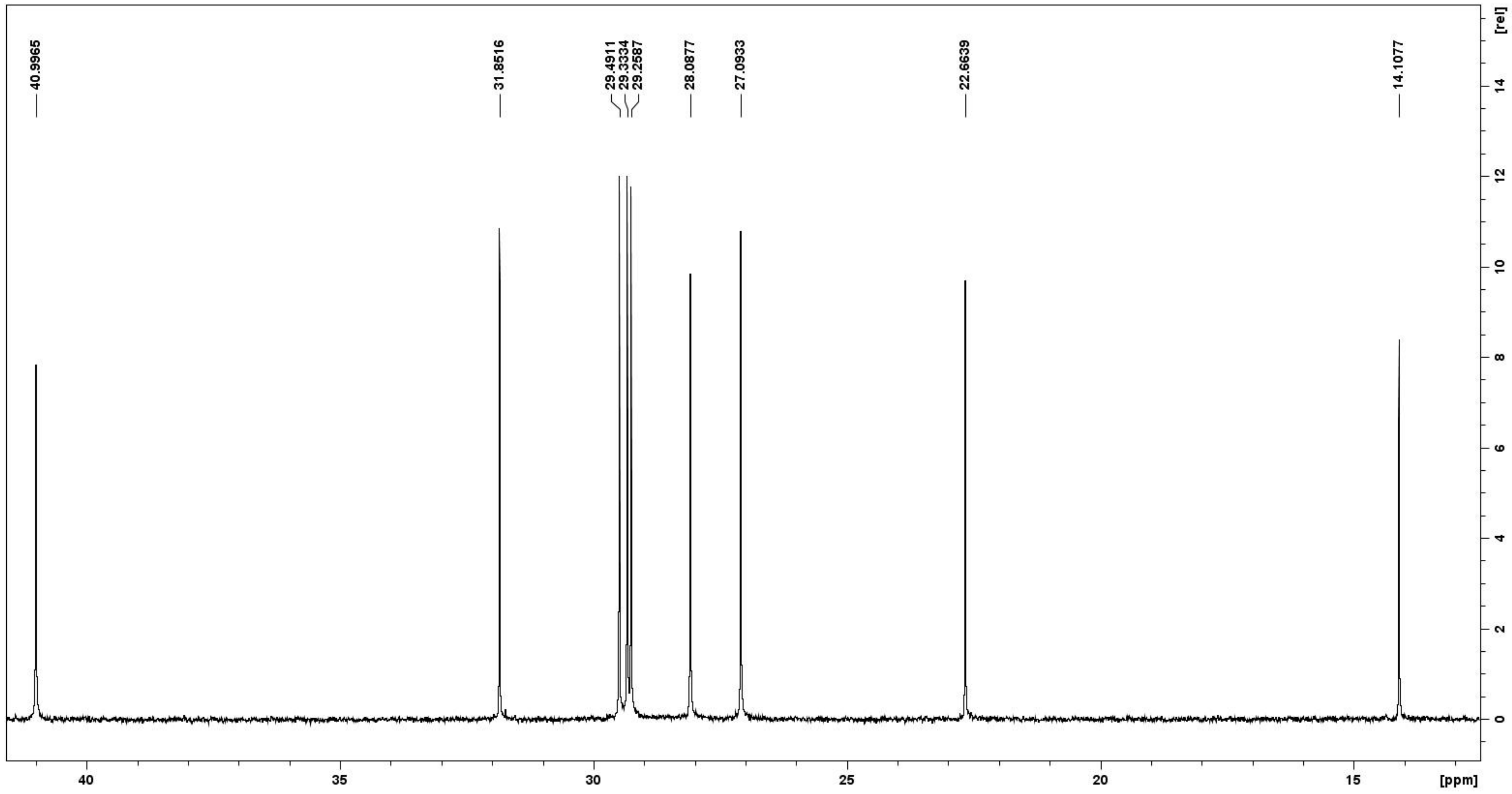

Expanded aliphatic region of $^{13}C\{^{1}H\}$NMR spectrum of NDIC9

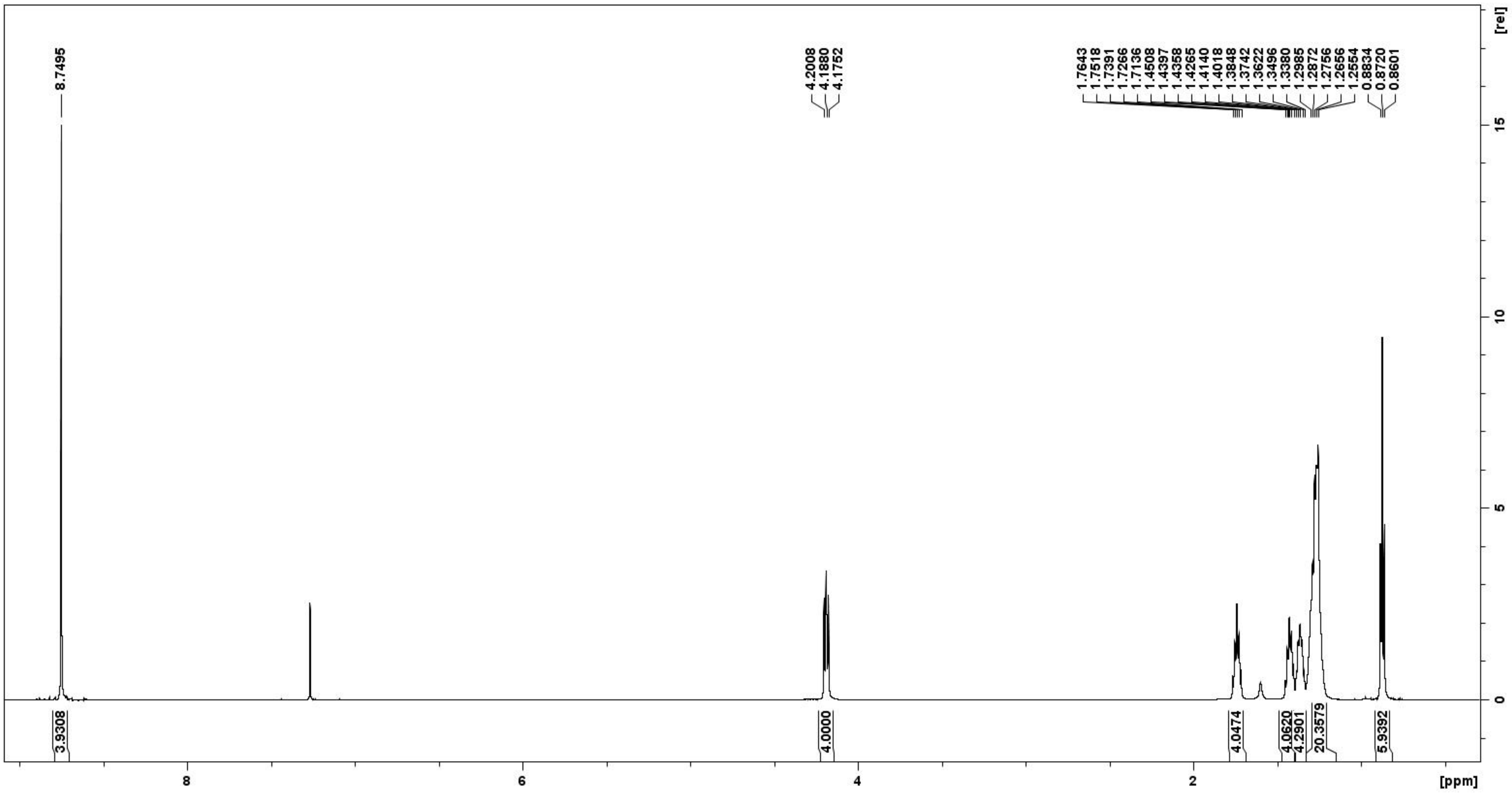

^{1}H NMR spectrum of NDIC10 ($CDCl_3$, 600MHz)

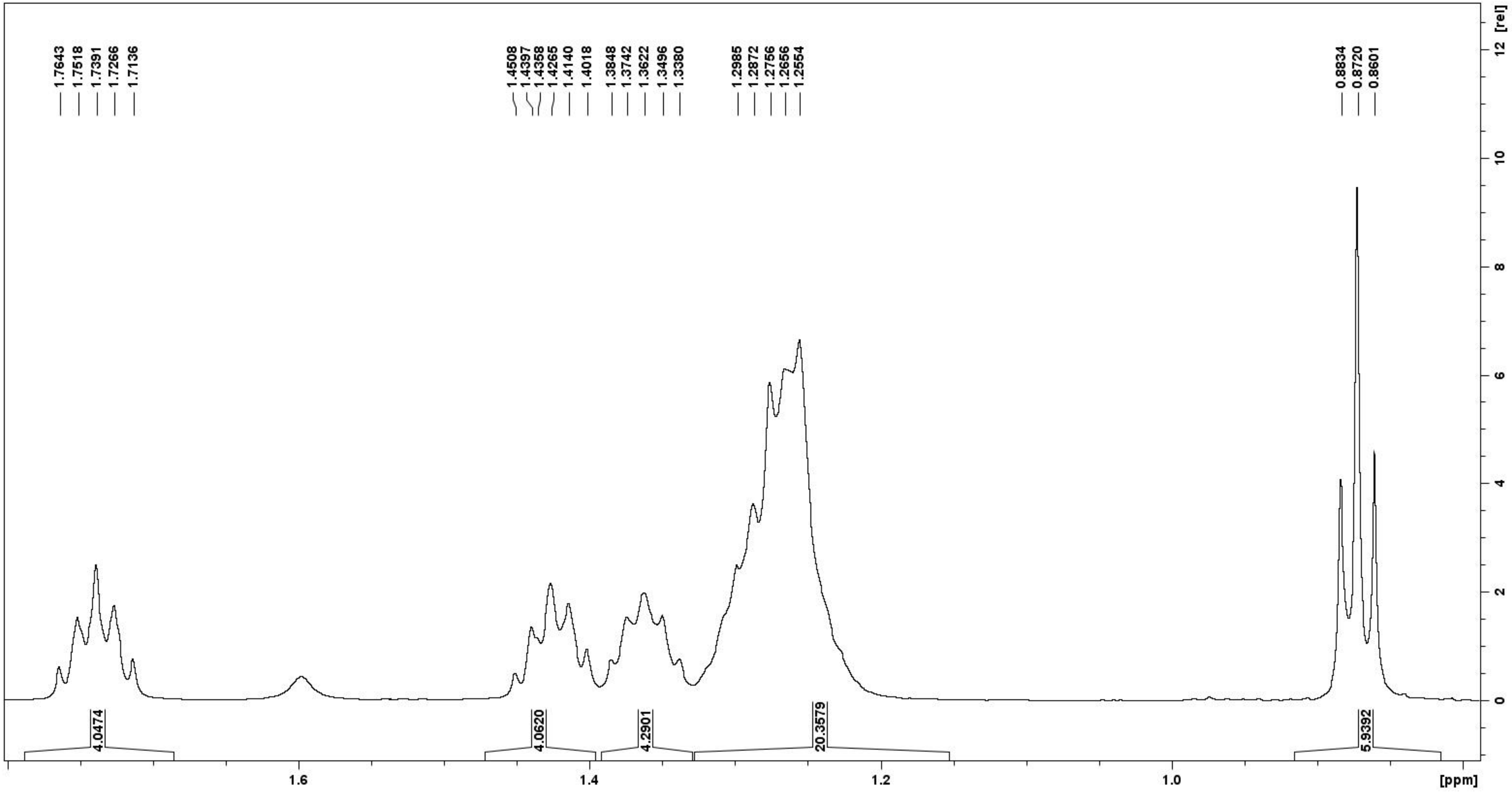

Expanded aliphatic region of ^{1}H NMR spectrum of NDIC10

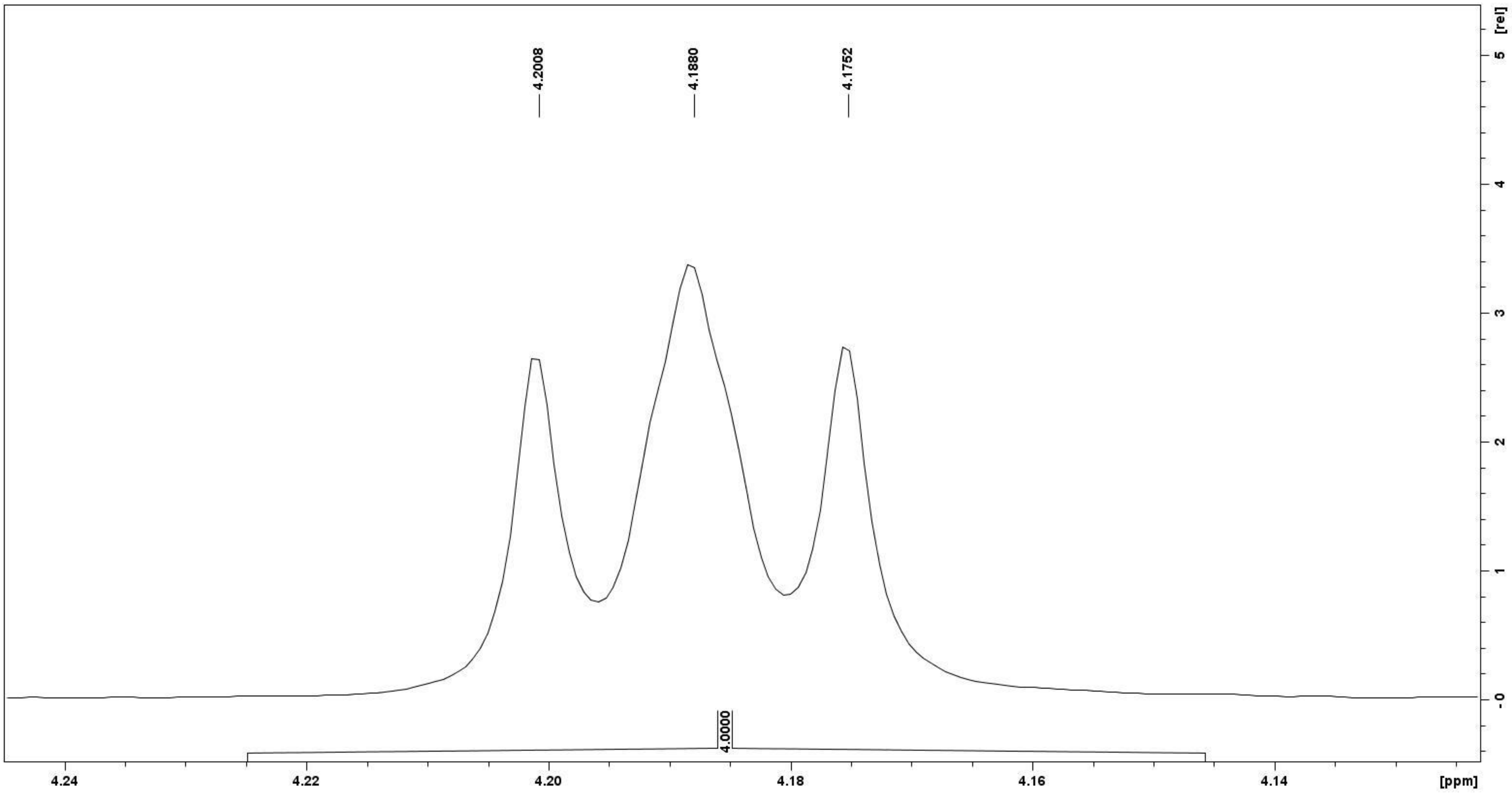

Expanded aliphatic region of ^{1}H NMR spectrum of NDIC10

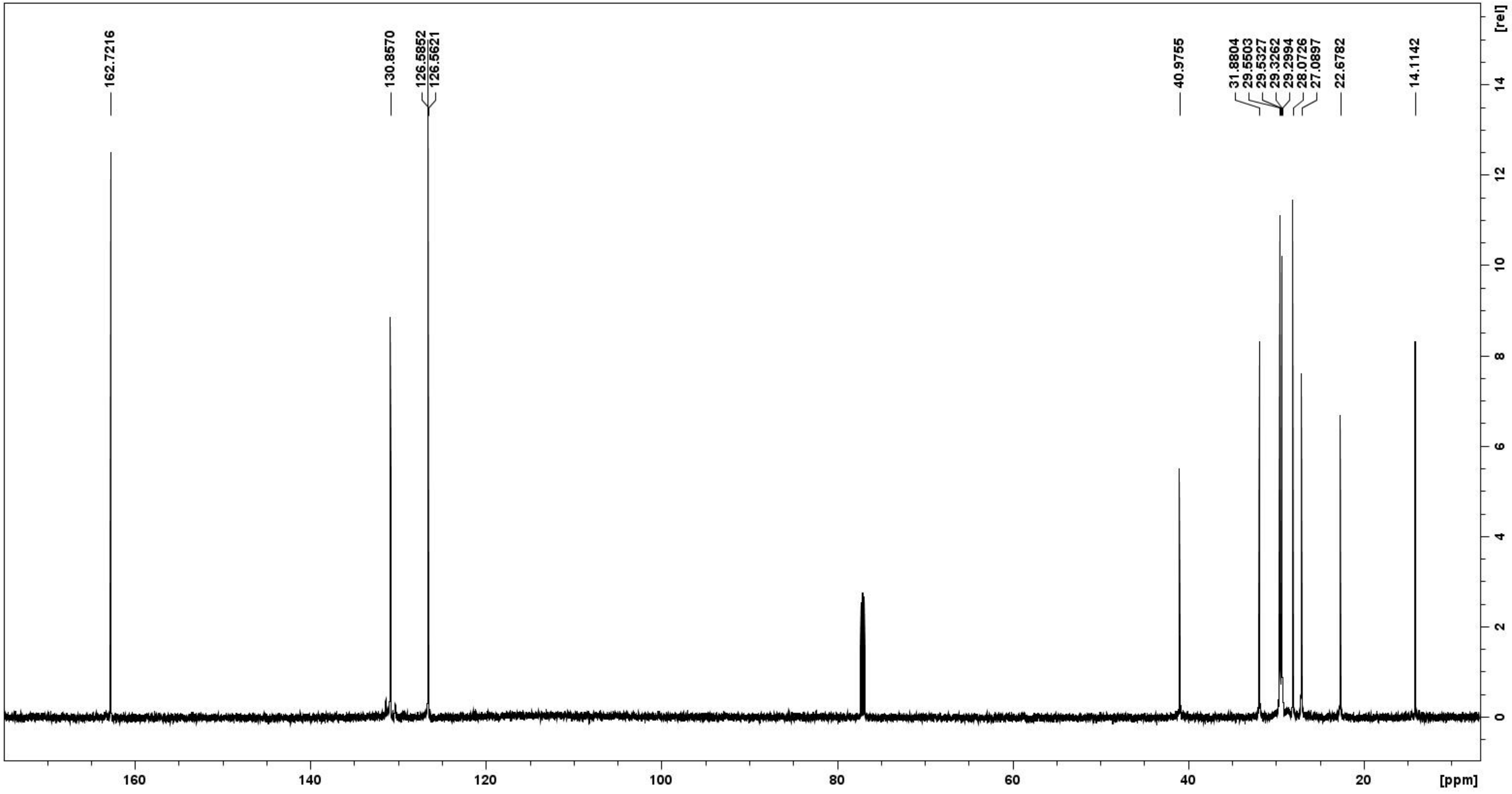

$^{13}C\{^{1}H\}$NMR spectrum of NDIC10 ($CDCl_3$, 151MHz)

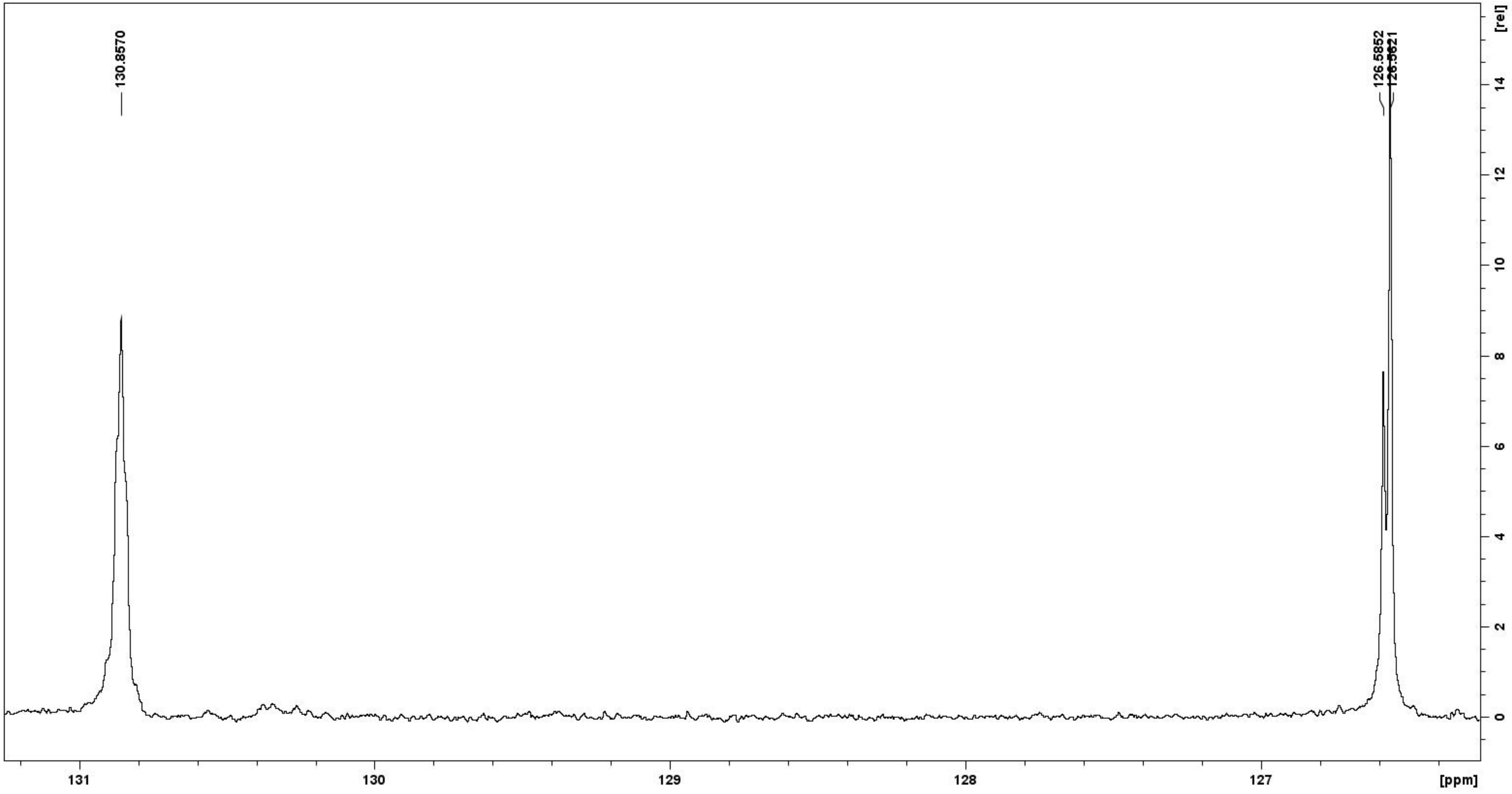

Expanded aromatic region of $^{13}C\{^{1}H\}$NMR spectrum of NDIC10

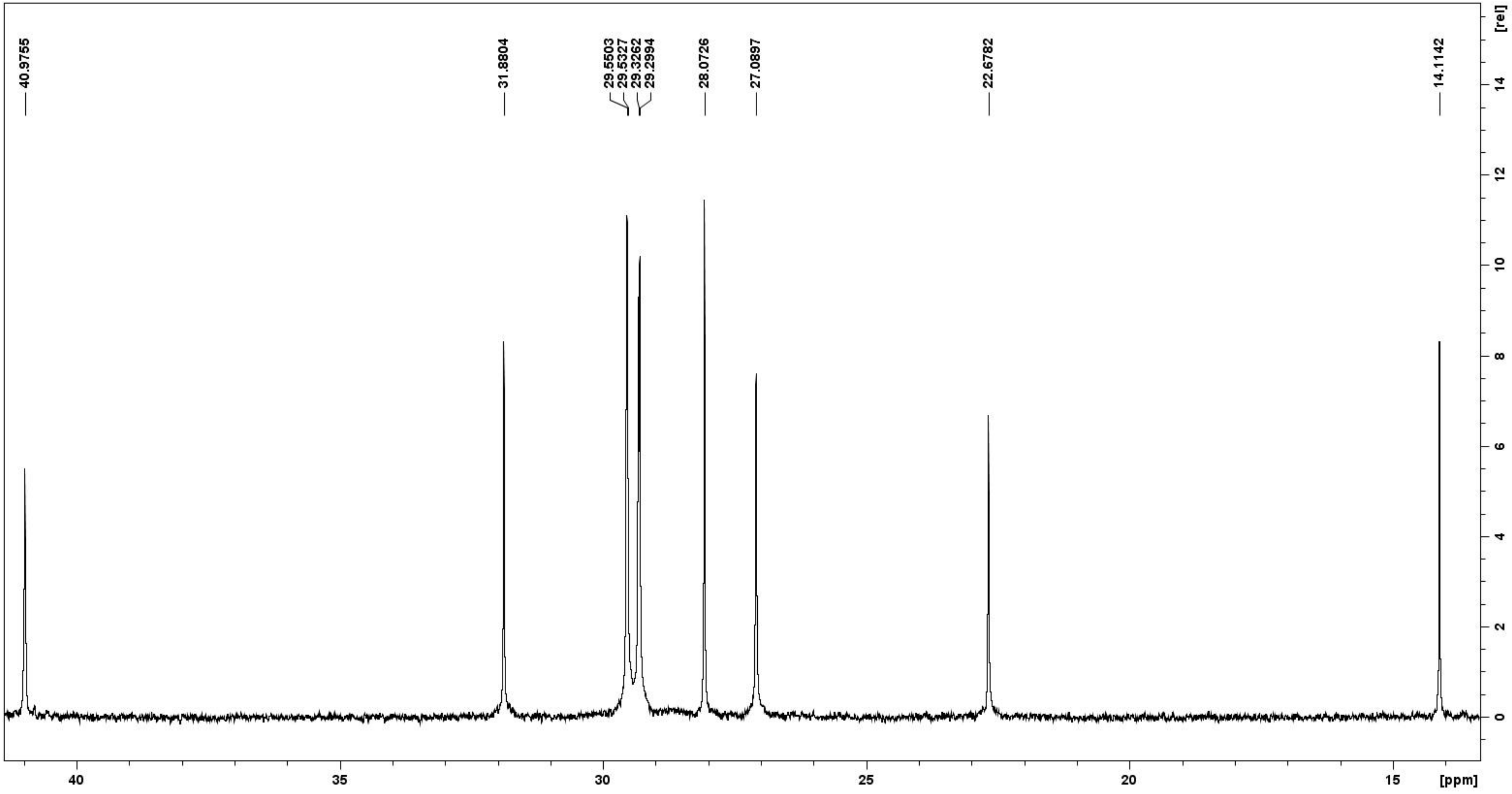

Expanded aliphatic region of $^{13}C\{^{1}H\}$NMR spectrum of NDIC10

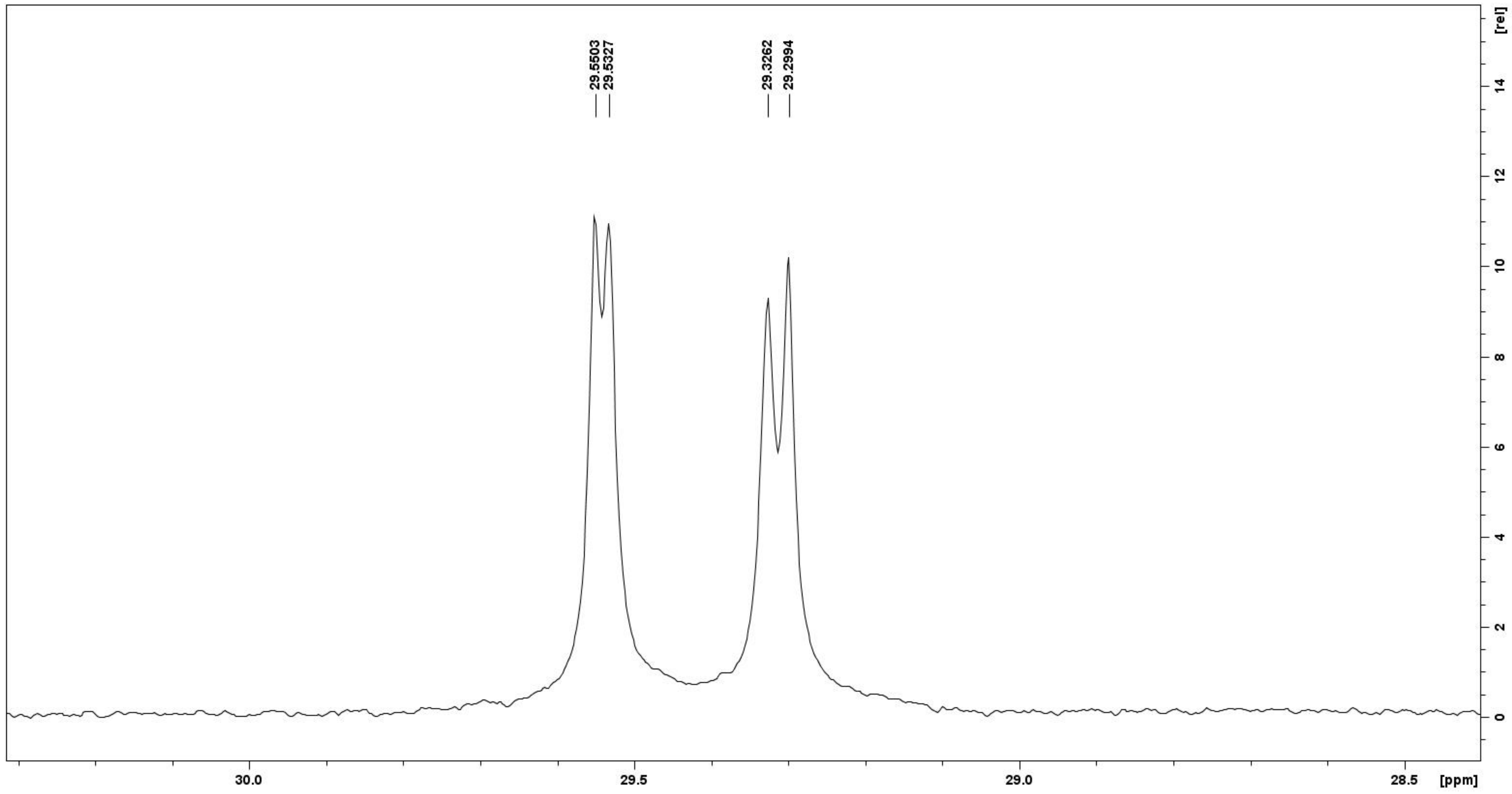

Expanded aliphatic region of $^{13}C\{^1H\}$NMR spectrum of NDIC10

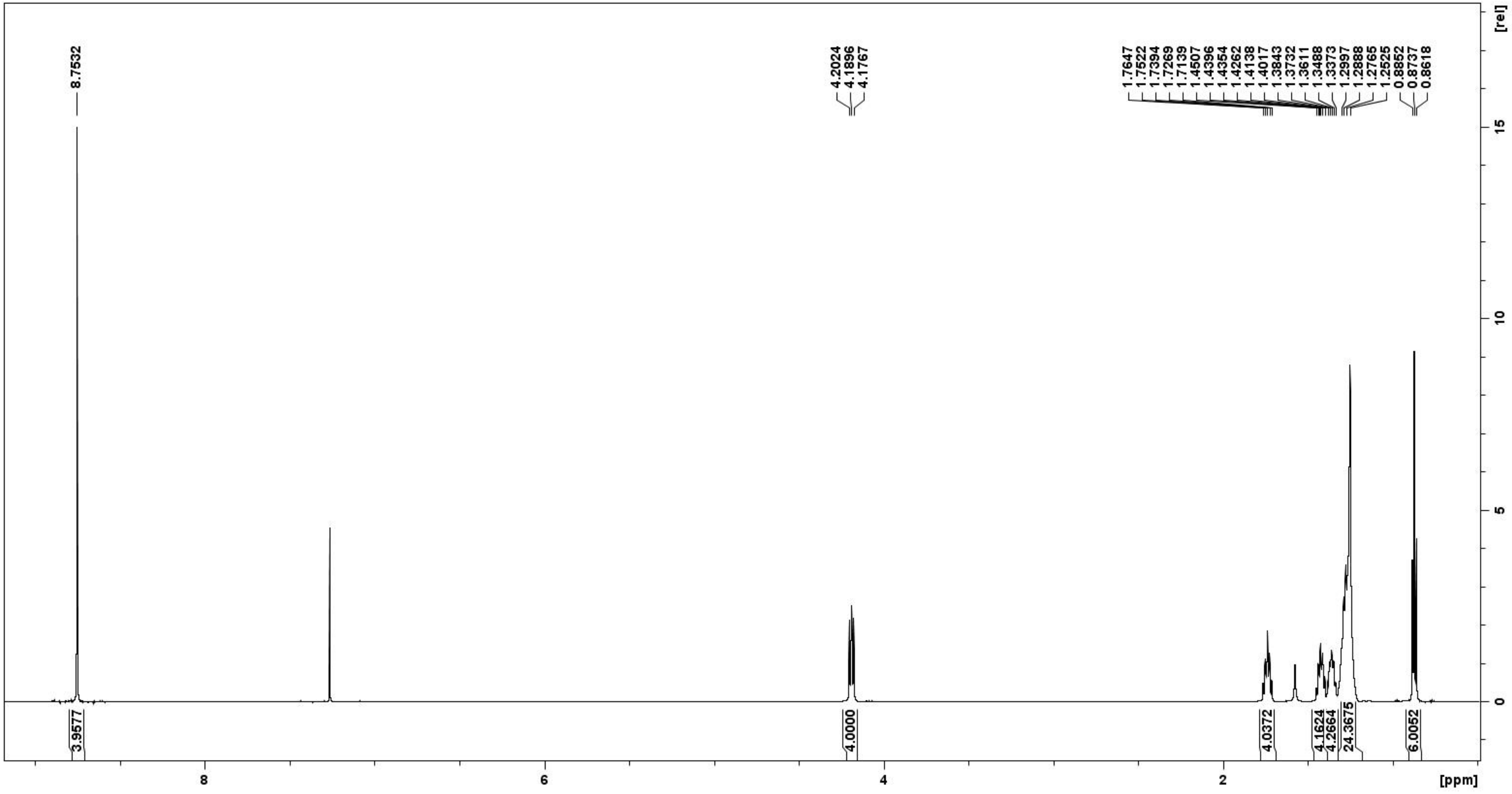

^{1}H NMR spectrum of NDIC11 ($CDCl_3$, 600MHz)

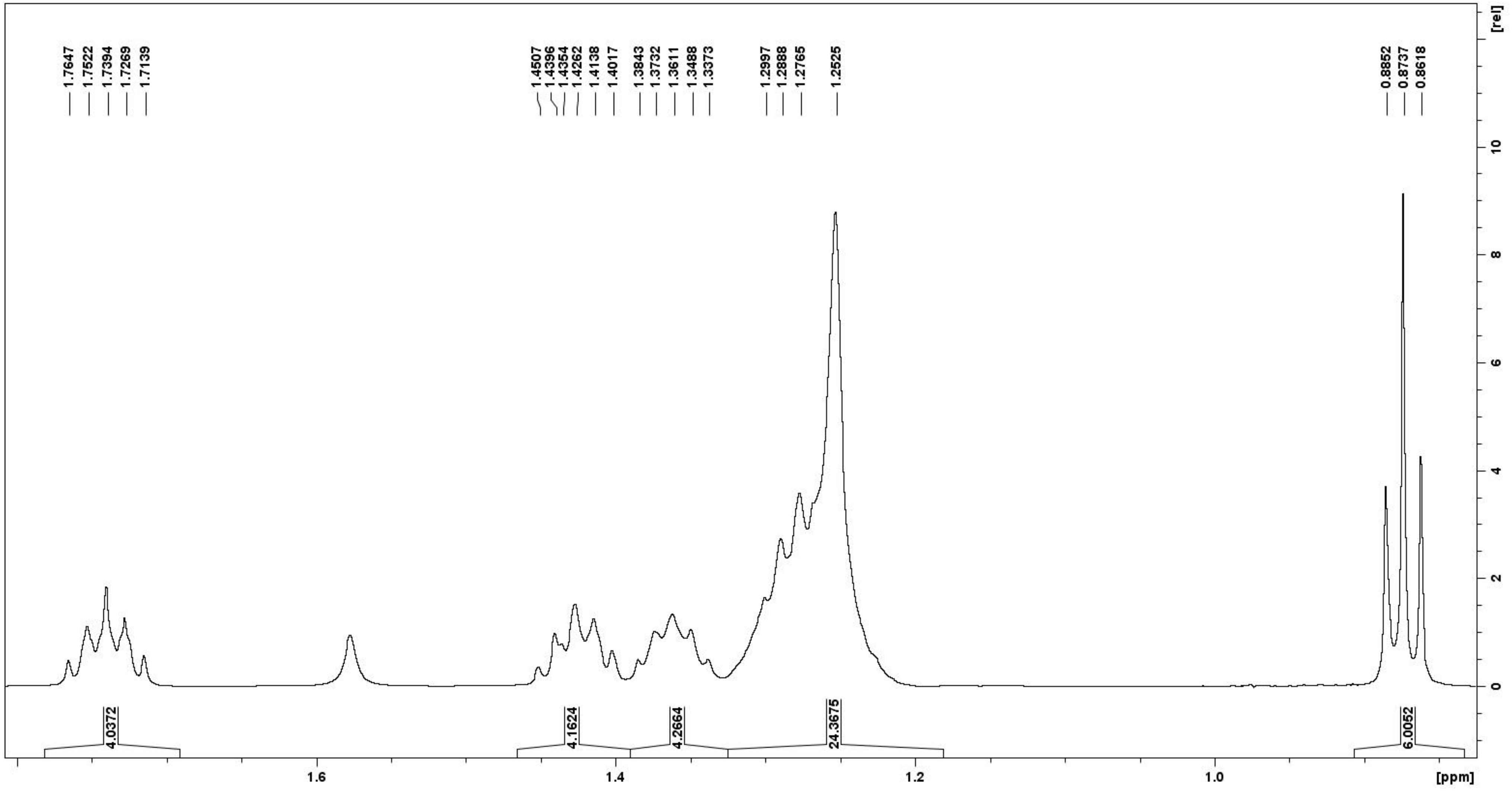

Expanded aliphatic region of ^{1}H NMR spectrum of NDIC11

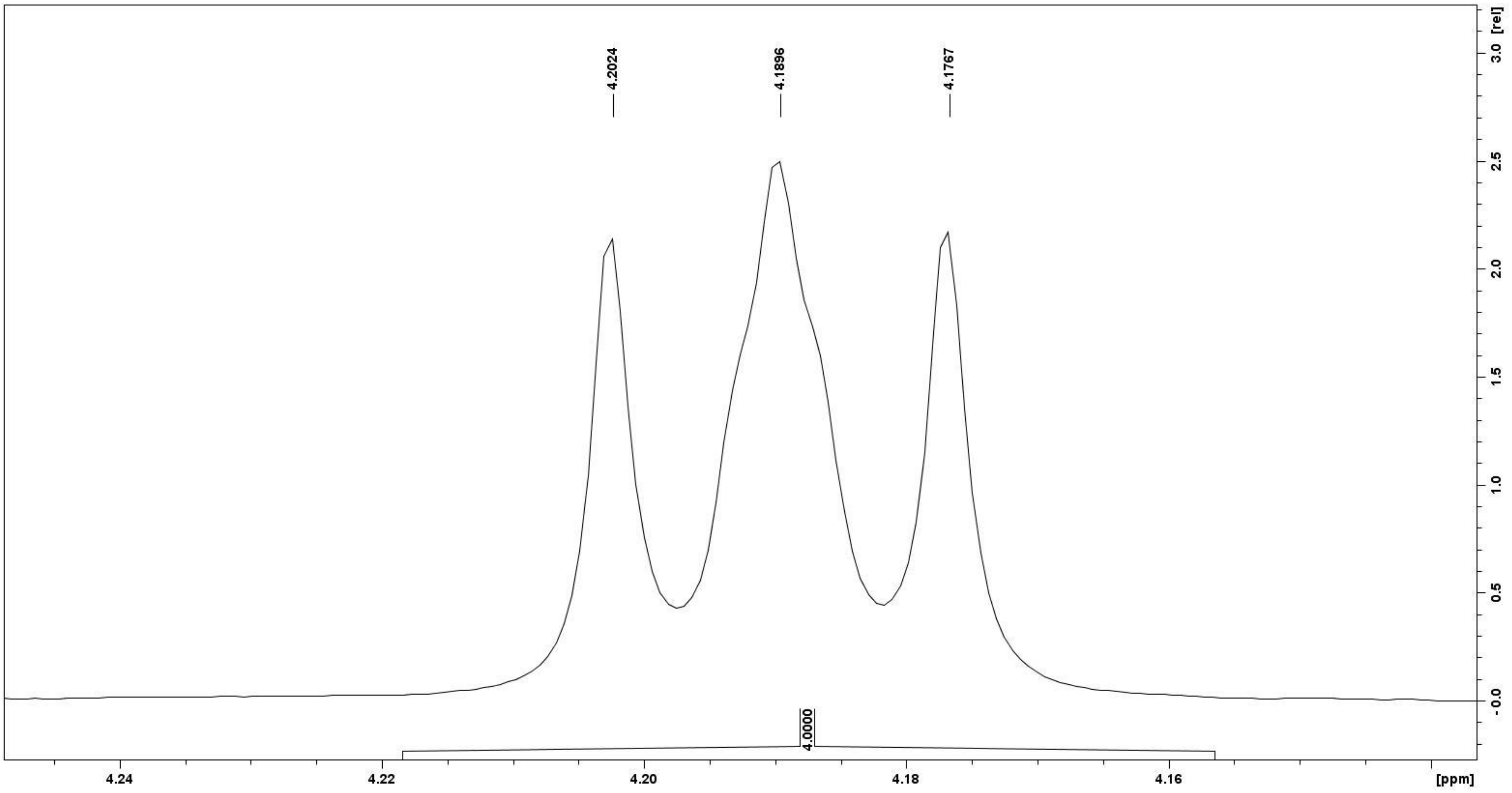

Expanded aliphatic region of ^{1}H NMR spectrum of NDIC11

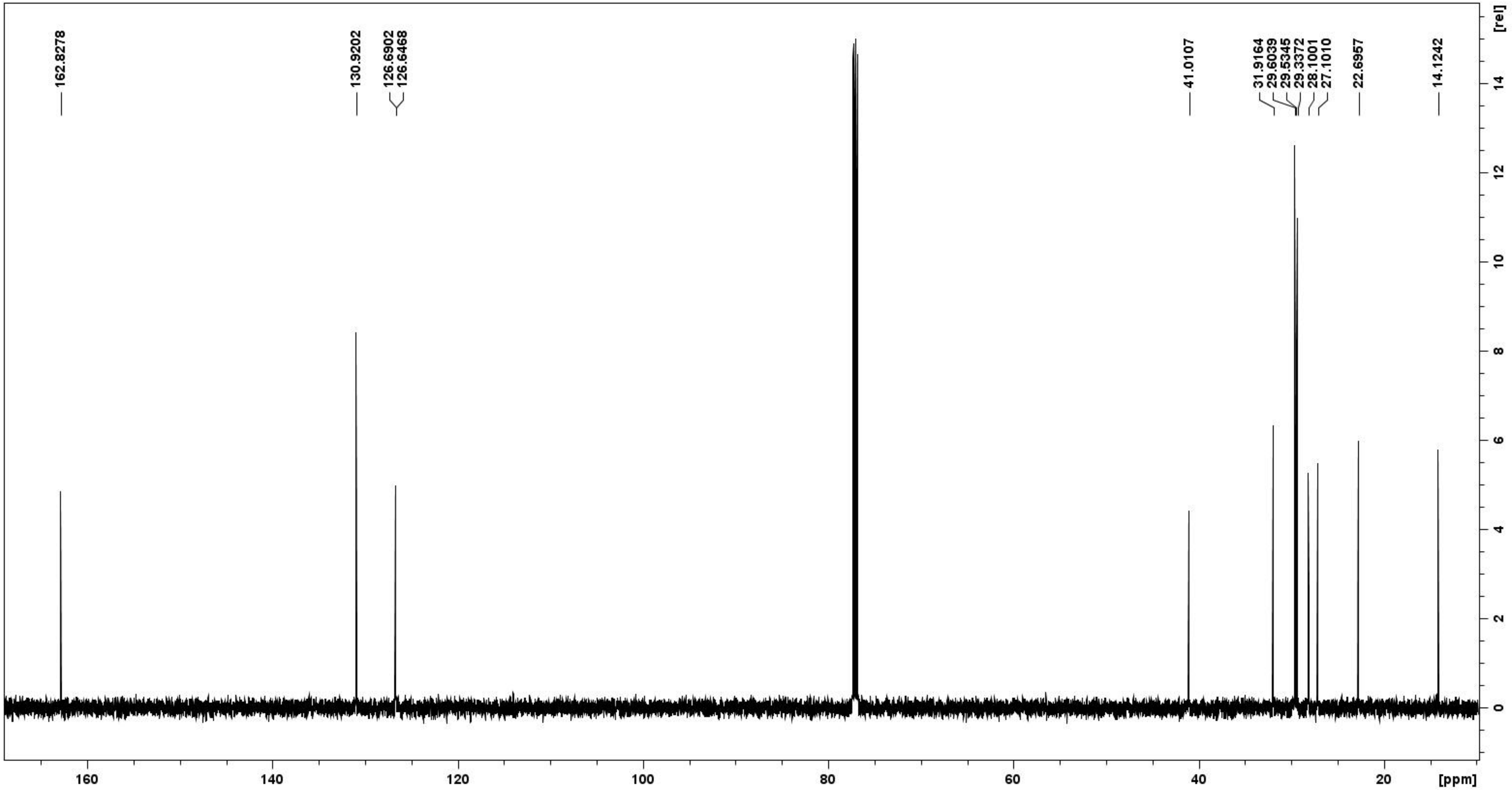

$^{13}C\{^{1}H\}$NMR spectrum of NDIC11 ($CDCl_3$, 151MHz)

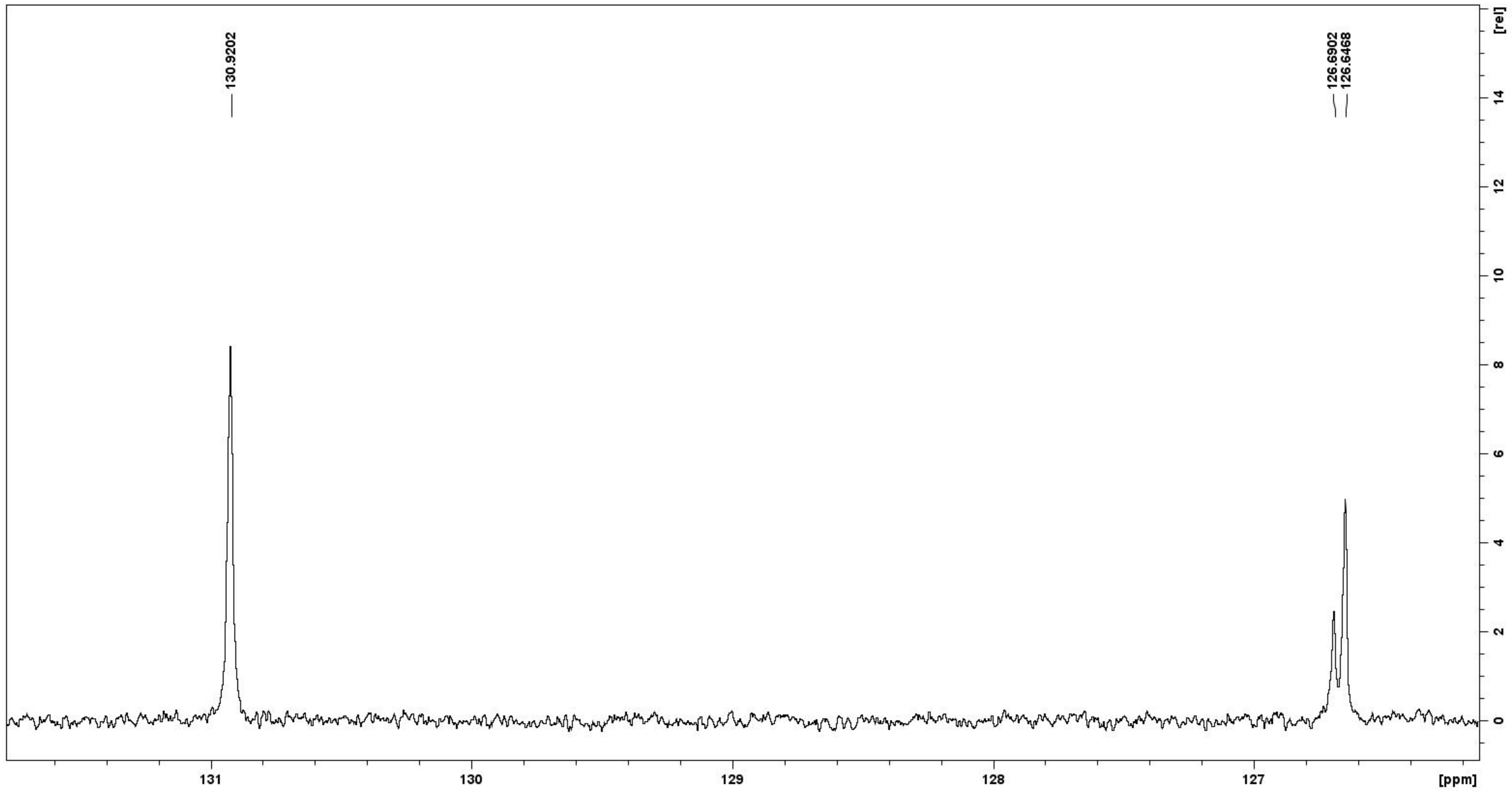

Expanded aromatic region of $^{13}C\{^{1}H\}$NMR spectrum of NDIC11

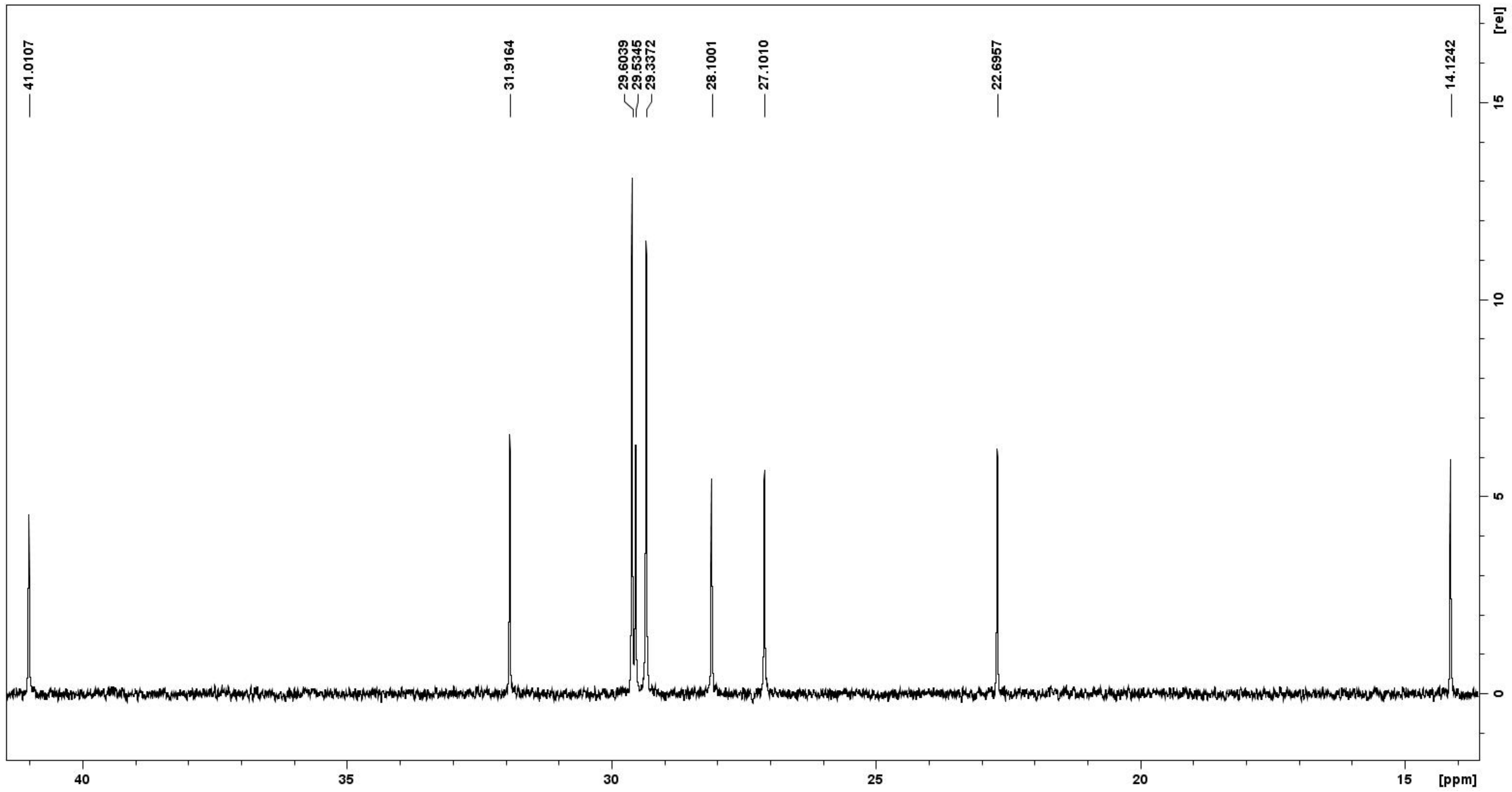

Expanded aliphatic region of $^{13}C\{^{1}H\}$NMR spectrum of NDIC11
