# Supplementary material for: Synthesis, Solution, and Solid State Properties of Homological Dialkylated Naphthalene Diimides—A Systematic Review of Molecules for Next-Generation Organic Electronics
Source: Molecules. 2023 Mar 25;28(7):2940. doi: 10.3390/molecules28072940 (PMC10096413; doi:10.3390/molecules28072940)
Supplement: Supplementary file 1 [file molecules-28-02940-s001.zip › NDI_derivatives_Chlebosz_supporting information_main_final_static_R1.pdf]

Supporting Information to the article (part 1)

Synthesis, Solution and Solid State Properties of Homological Dialkylated Naphthalene Diimides. A Systematic Review of Molecules for Next-Generation Organic Electronics

Dorota Chlebosz[1,2,], Waldemar Goldeman[3], Krzysztof Janus[1,2], Michał Szuster[1], Adam Kiersnowski[1,2,*]

[1] Department of Physical and Quantum Chemistry, Wroclaw University of Science and Technology, Wybrzeze Wyspianskiego 27, 50-370 Wroclaw, Poland

[2] The Leibniz Institute of Polymer Research, Hohe Strasse 6, D-01069 Dresden, Germany

[3] Department of Medicinal and Organic Chemistry, Wroclaw University of Science and Technology, Wybrzeze Wyspianskiego 27, 50-370 Wroclaw, Poland

*correspondence: Adam Kiersnowski, adam.kiersnowski@pwr.edu.pl

Table of contents

SI-1. Summary of NMR data for the synthesized NDIs··························· page S3

SI-2. UV-Vis study: spectra and solubility·· page S5

SI-3. FTIR spectra of NDIs·· page S7

SI-4. Crystal structure of NDIs·· page S9

SI-5. Thermal properties of NDIs: TGA and DSC study························ page S11

SI-6. Exemplary OFET characteristics of NDIC7·································· page S14

SI-7. Supporting references··· page S15

SI-1. Summary of NMR data for the synthesized NDIs

Table S1. ^{13}C NMR data of the studied NDIs. Chemical shifts of the subsequent methylene carbon atoms in alkyl chains are given in columns under corresponding groups in the header. The carbon atoms are counted from the methyl group to the α–methylene group protons ($N\underline{CH_2}CH_2$).

	Two 4° aromatic carbons[1)]	ArCH	C=O	NCH_2	CH_2	CH_2	CH_2	CH_2	CH_2	CH_2	CH_2	CH_2	CH_2	CH_3
**NDIC3**	126.61 and 126.67	130.92	162.83	-	-	-	-	-	-	-	-	42.42	21.38	11.49
**NDIC4**	126.64 and 126.69	130.92	162.84	-	-	-	-	-	-	-	40.76	30.17	20.35	13.81
**NDIC5**	126.51*	130.82	162.67	-	-	-	-	-	-	40.91	29.16	27.71	22.38	13.96
**NDIC6**	126.60 and 126.64	130.87	162.78	-	-	-	-	-	40.98	31.49	28.02	26.74	22.54	14.04
**NDIC7**	126.60 and 126.57	130.87	162.74	-	-	-	-	40.98	31.73	28.98	28.08	27.04	22.59	14.07
**NDIC8**	126.62 and 126.65	130.89	162.79	-	-	-	41.00	31.80	29.29	29.19	28.09	27.10	22.64	14.09
**NDIC9**	126.61 and 126.65	130.90	162.79	-	-	41.00	31.85	29.49	29.33	29.26	28.09	27.09	22.66	14.11
**NDIC10**	126.56 and 126.58	130.86	162.72	-	40.98	31.88	29.55	29.53	29.33	29.30	28.07	27.09	22.68	14.11
**NDIC11**	126.65 and 126.69	130.92	162.83	41.01	31.92	29.60*		29.53	29.33*		28.10	27.10	22.70	14.12

*Overlapped signals of two carbons; [1)] two signals of two groups of quaternary carbon atoms: C9 and C10, and C1, C4, C5 and C8

Table S2. ^{1}H NMR data of the studied NDIs. Chemical shifts of the subsequent methylene protons in alkyl chains are given in columns under corresponding groups in the header. The protons are counted from the methyl group to the α–methylene group protons (N<u>CH_2</u>CH_2). Multiplets in the spectra are marked as: s = singlet, d = dublet, t = triplet, q = quartet, quint = quintet, sext = sextet, m = multiplet (complex pattern), br = broad.

	Naphthyl-H	NCH_2	CH_2	CH_2	CH_2	CH_2	CH_2	CH_2	CH_2	CH_2	CH_2	CH_3
**NDIC3**	8.75 (s)	-	-	-	-	-	-	-	-	4.17 (m)*	1.78 (sext, 7.5Hz)	1.03 (t, 7.4Hz)
**NDIC4**	8.78 (s)	-	-	-	-	-	-	-	4.23 (m)*	1.76 (m)	1.48 (sext, 7.5Hz)	1.02 (t, 7.4Hz)
**NDIC5**	8.76 (s)	-	-	-	-	-	-	4.21 (m)*	1.77 (m)	1.38-1.48 (m)		0.94 (t, 7.1Hz)
**NDIC6**	8.78 (s)	-	-	-	-	-	4.21 (m)*	1.77 (m)	1.46 (m)	1.33-1.41 (m)		0.92 (t, 7.0Hz)
**NDIC7**	8.76 (s)	-	-	-		4.21 (m)*	1.76 (m)	1.45 (m)	1.39 (m)	1.27-1.36 (m)		0.90 (t, 7.0Hz)
**NDIC8**	8.77 (s)	-	-	-	4.21 (m)*	1.76 (m)	1.45 (m)	1.39 (m)	1.25-1.35 (m)			0.90 (t, 7.1Hz)
**NDIC9**	8.75 (s)	-	-	4.19 (m)*	1.74 (m)	1.43 (m)	1.36 (m)	1.22-1.35 (m)				0.87 (t, 7.1Hz)
**NDIC10**	8.75 (s)	-	4.19 (m)*	1.74 (m)	1.43 (m)	1.36 (quint, 7.2Hz)	1.20-1.33 (m)					0.87 (t, 7.2Hz)
**NDIC11**	8.75 (s)	4.19 (m)*	1.74 (m)	1.43 (m)	1.36 (quint, 7.4Hz)	1.20-1.32 (m)						0.87 (t, 7.1Hz)

*second-order AA'XX' spin system (for comments see the main text of manuscript)

SI-2. UV-Vis study: spectra and solubility

Table S3. Wavelength (λ) and molar absorptivity (ε) of the main bands of NDIs (spectra shown in Figure S1). Spectra acquired for 0.1 mmol/L solutions in chloroform.

	λ_1 [nm]	$\varepsilon_1 \cdot 10^{-3}$ [$M^{-1}cm^{-1}$]	λ_2 [nm]	$\varepsilon_2 \cdot 10^{-3}$ [$M^{-1}cm^{-1}$]	λ_3 [nm]	$\varepsilon_3 \cdot 10^{-3}$ [$M^{-1}cm^{-1}$]
**NDIC3**	342	12.6	360	20.4	380	24.8
**NDIC4**	342	11.5	360	19.2	380	24.1
**NDIC5**	343	12.7	360	21.9	380	27.7
**NDIC6**	342	12.7	360	22.0	380	27.8
**NDIC7**	342	12.9	360	22.2	380	28.1
**NDIC8**	343	13.7	360	23.8	381	30.3
**NDIC9**	342	16.0	360	21.6	381	27.3
**NDIC10**	342	13.1	360	22.7	381	28.8
**NDIC11**	343	13.0	360	22.3	380	28.3

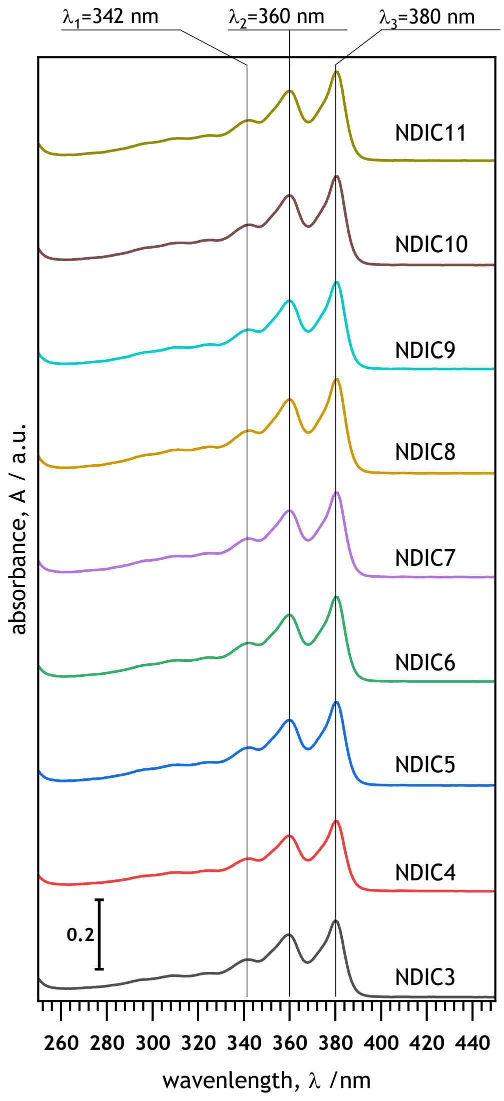

Figure S1. UV-VIS spectra of the NDIs in the study ($CHCl_3$, 0.1 mM).

Table S4. Solubility of NDIs in n-heptane

	solubility	
	mg/mL	mmol/L
**NDIC3**	0.048±0.003	0.137±0.008
**NDIC4**	0.056 ± 0.009	0.148±0.024
**NDIC5**	0.172 ± 0.005	0.423±0.012
**NDIC6**	0.321 ± 0.007	0.739±0.016
**NDIC7**	0.268 ± 0.018	0.579±0.039
**NDIC8**	0.327 ± 0.010	0.666±0.020
**NDIC9**	0.440 ± 0.053	0.848±0.102
**NDIC10**	0.472 ± 0.057	0.863±0.104
**NDIC11**	0.619 ± 0.047	1.08±0.082

Table S5. Solubility of NDIs in toluene

	solubility	
	mg/mL	mmol/L
**NDIC3**	6.3 ± 0.4	18.0±1. 1
**NDIC4**	3.9 ± 0.3	10.3±0.8
**NDIC5**	15.4 ± 0.4	37.9±1.0
**NDIC6**	24.0 ± 0.5	55.2±1.2
**NDIC7**	20.9 ± 0.8	45.2±1.2
**NDIC8**	20.5 ± 0.8	41.8±1.6
**NDIC9**	5.1 ± 0.5	9.8±1.0
**NDIC10**	10.2 ± 0.4	18.7±0.7
**NDIC11**	2.0 ± 0.4	3.5±0.7

Table S6. Solubility of NDIs in 1,2-dichlorobenzene

	solubility	
	mg/mL	mmol/L
**NDIC3**	26.8 ± 0.4	76.5±1. 1
**NDIC4**	9.8 ± 0.2	25.9±0.5
**NDIC5**	31.9 ± 0.6	78.6±1.5
**NDIC6**	42.8 ± 0.6	98.5±1.4
**NDIC7**	39.0 ± 1.2	84.3±2.6
**NDIC8**	34.9 ± 0.6	71.2±1.2
**NDIC9**	9.4 ± 0.2	18.1±0.4
**NDIC10**	18.1 ± 0.8	33.1±1.5
**NDIC11**	3.5 ± 0.2	6.1±0.4

SI-3. FTIR spectra of NDIs

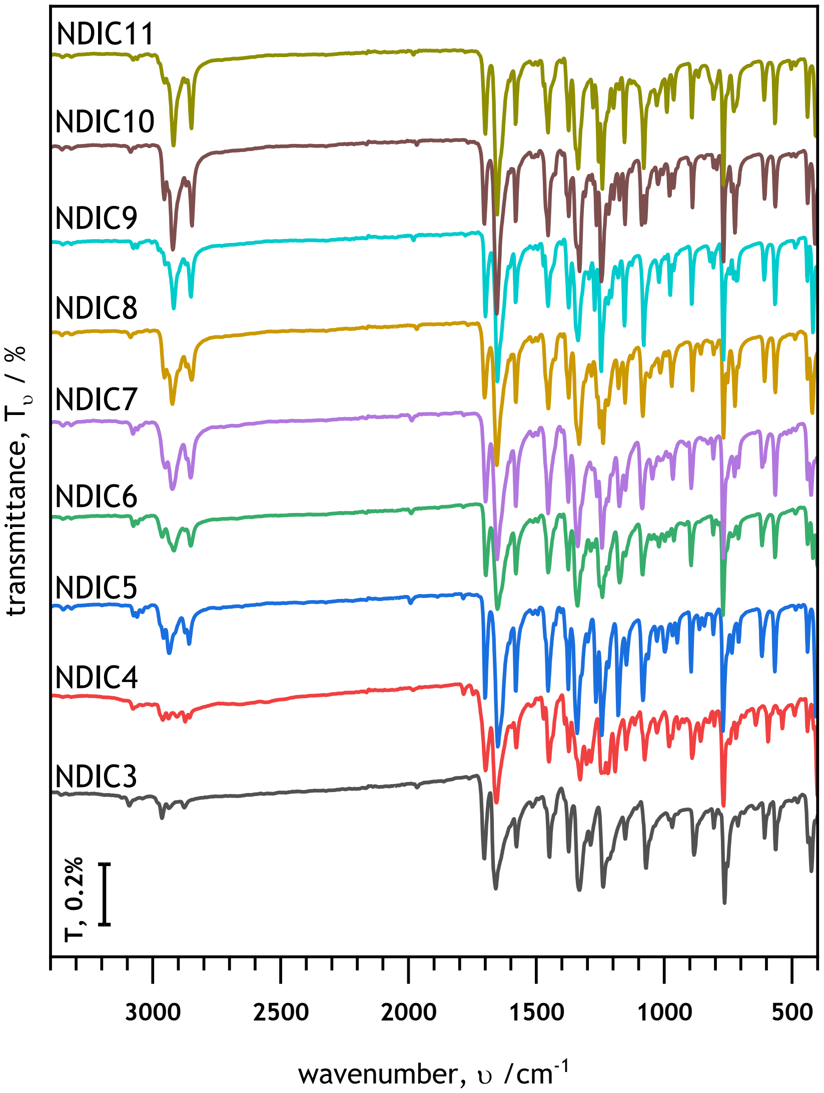

Figure S2. Series of FTIR spectra of all investigated NDIs. For the sake of clarity the spectra are shifted along the transmittance scale. The transmittance scale bar in the lower left corner. Abbreviations are explained in the main part of the paper.

Table S7. Positions and intensities of the main FTIR absorption bands of the NDIs

	aromatic				alkyl		symmetric and asymmteric imide C=O stretching	C-N stretching
	**C-H stretchig**	**C=C stretching**	**C-H in-plane bending**	**C-H out-of-plane bending**	**C-H stretching**	**C-H bending**		
**NDIC3**	3043<br>3061<br>3076(vw)	1581(m)	1080(s)<br>1242(s)	770(s)	2850<br>2920<br>2953(w)	1373(m)<br>1454(m)	1653(vs)<br>1699(s)	1337(s)
**NDIC4**	3040<br>3064<br>3076(vw)	1578(m)	1076(s)<br>1246(s)	768(s)	2858<br>2873<br>2939<br>2960(w)	1373(m)<br>1450(m)	1654(vs)<br>1699(s)	1329(s)
**NDIC5**	3041<br>3063<br>3074(vw)	1580(m)	1084(s)<br>1180(s)<br>1244(s)	770(s)	2858<br>2874<br>2935<br>2958(w)	1375(m)<br>1454(m)	1650(vs)<br>1701(s)	1340(s)
**NDIC6**	3041<br>3061<br>3076(vw)	1580(m)	1084(s)<br>1175(s)<br>1242(s)	770(s)	2852<br>2916<br>2964(w)	1375(m)<br>1454(m)	1652(vs)<br>1697(s)	1338(s)
**NDIC7**	3041<br>3061<br>3078(vw)	1580(m)	1086(s)<br>1175(s)<br>1244(s)	770(s)	2852<br>2868<br>2924<br>2951(m)	1375(m)<br>1454(m)	1653(vs)<br>1699(s)	1338(s)
**NDIC8**	3043<br>3070<br>3088(vw)	1580(m)	1084(s)<br>1154(m)<br>1238(s)	768(s)	2848<br>2868<br>2924<br>2955(m)	1374(m)<br>1452(m)	1655(vs)<br>1703(s)	1333(s)
**NDIC9**	3041<br>3061<br>3076(vw)	1581(m)	1080(s)<br>1155(m)<br>1246(s)	768(s)	2850<br>2918<br>2951(m)	1373(m)<br>1454(m)	1652(vs)<br>1699(s)	1337(s)
**NDIC10**	3045<br>3068<br>3087(vw)	1581(m)	1074(s)<br>1088(s)<br>1153(m)<br>1246(s)	768(s)	2847(m)<br>2922(s)<br>2955(m)	1373(m)<br>1454(m)	1655(vs)<br>1703(s)	1331(s)
**NDIC11**	3043<br>3061<br>3076(vw)	1581(m)	1080(s)<br>1155(m)<br>1242(s)	768(s)	2848(m)<br>2918(s)<br>2953(w)	1375(m)<br>1454(m)	1653(vs)<br>1699(s)	1335(s)

Peaks intensity abbreviation: vs, very strong; s, strong; m, medium; w, weak; vw, very weak

SI-4. Crystal structure of NDIs

SI-4.1 Selected crystal data and structure refinement for NDIC7, NDIC9 and NDIC11:

Crystal Data for **NDIC7:** *CCDC number* ***2242898*** $C_{28}H_{34}N_2O_4$ (M =462.57 g/mol): monoclinic, space group P2$_1$/n (no. 14), a = 7.8730(2) Å, b = 4.84356(13) Å, c = 33.0246(9) Å, β = 95.025(3)°, V = 1254.50(6) Å^3, Z = 2, T = 293(2) K, μ(MoKα) = 0.082 mm^{-1}, $Dcalc$ = 1.225 g/cm^3, 12647 reflections measured (5.234° ≤ 2Θ ≤ 55.124°), 2655 unique (R_{int} = 0.0226, R_{sigma} = 0.0179) which were used in all calculations. The final R_1 was 0.0463 (I > 2σ(I)) and wR_2 was 0.1182 (all data).

Crystal Data for **NDIC9:** *CCDC number* ***2242899*** $C_{32}H_{42}N_2O_4$ (M =518.67 g/mol): monoclinic, space group P2$_1$/c (no. 14), a = 7.8486(5) Å, b = 4.8383(5) Å, c = 37.741(2) Å, β = 95.026(6)°, V = 1427.66(19) Å^3, Z = 2, T = 293(2) K, μ(AgKα) = 0.050 mm^{-1}, $Dcalc$ = 1.207 g/cm^3, 4992 reflections measured (6.7° ≤ 2Θ ≤ 44.368°), 3040 unique (R_{int} = 0.0148, R_{sigma} = 0.0275) which were used in all calculations. The final R_1 was 0.0439 (I > 2σ(I)) and wR_2 was 0.1189 (all data).

Crystal Data for **NDIC11:** *CCDC number* ***2242897*** $C_{36}H_{50}N_2O_4$ (M =574.78 g/mol): monoclinic, space group P2$_1$/c (no. 14), a = 7.8478(6) Å, b = 4.8691(3) Å, c = 42.509(3) Å, β = 92.142(6)°, V = 1623.19(19) Å^3, Z = 2, T = 290(2) K, μ(MoKα) = 0.076 mm^{-1}, $Dcalc$ = 1.176 g/cm^3, 5554 reflections measured (3.836° ≤ 2Θ ≤ 55.312°), 3274 unique (R_{int} = 0.0179, R_{sigma} = 0.0358) which were used in all calculations. The final R_1 was 0.0492 (I > 2σ(I)) and wR_2 was 0.1192 (all data).

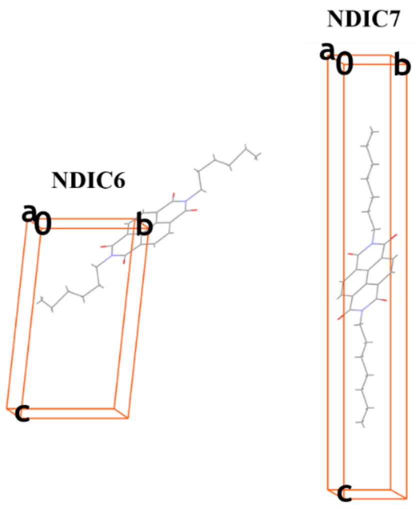

Figure S3. Unit cells of NDIC6 and NDIC7.

SI-4.2. Exemplary calculation of the interplanar spacings (d_{hkl}):

The interplanar spacings (d_{hkl}) were calculated from the Miller indices (hkl) and the values of the reticular parameters (unit cell axes). Equations to calculate the interplanar spacings (d_{hkl}) in a monoclinic (Equation ES1) and triclinic (Equation ES2) crystal system are shown below. The Miller indices corresponding to crystallographic planes parallel to the NDI cores are given in Table S4. Distance between these planes are considered π-π stacking distance (see main article).

Monoclinic: $$\frac{1}{d_{hkl}^2} = \frac{1}{sin^2\beta}\left(\frac{h^2}{a^2} + \frac{k^2 sin^2\beta}{b^2} + \frac{l^2}{c^2} - \frac{2hlcos\beta}{ac}\right)$$ **ES1**

Triclinic: $$\frac{1}{d_{hkl}^2} = \frac{1}{V^2}(S_{11}h^2 + S_{22}k^2 + S_{33}l^2 + 2S_{12}hk + 2S_{23}kl + 2S_{13}hl)$$ **ES2**

where:

$V -$ *volume of unit cell*

$S_{11} = b^2c^2sin^2\alpha$

$S_{22} = a^2c^2sin^2\beta$

$S_{33} = a^2b^2sin^2\gamma$

$S_{12} = abc^2(cos\alpha cos\beta - cos\gamma)$

$S_{23} = a^2bc(cos\beta cos\gamma - cos\alpha)$

$S_{13} = ab^2c(cos\gamma cos\alpha - cos\beta)$

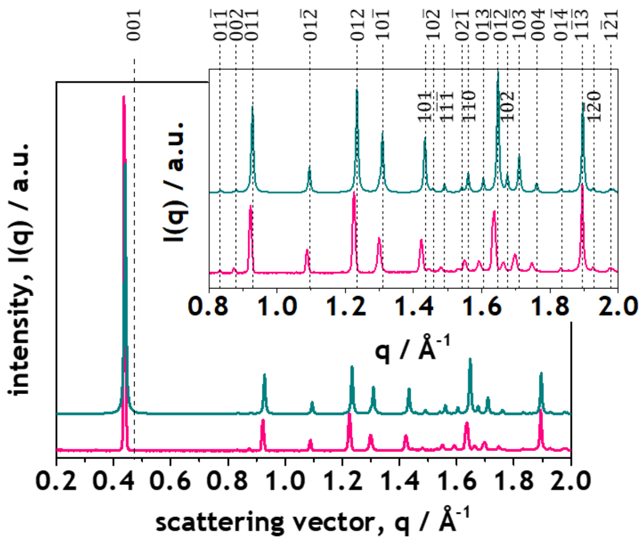

Figure S4. Exemplary one-dimensional I(q) profiles of NDIC6: simulated in Mercury software (blue curve) and experimental (pink curve). Vertical dash lines mark positions of NDIC6 peaks; the numbers denote Miller indices of the peaks.

Table S8. π-π stacking planes

	π-π **stacking plane**
**NDIC3**	-
**NDIC4**	$(1\bar{1}\bar{2})$
**NDIC5**	$(11\bar{5})$
**NDIC6**	$(1\bar{1}\bar{3})$
**NDIC7**	(116)
**NDIC8**	$(1\bar{1}0)$
**NDIC9**	$(11\bar{8})$
**NDIC10**	(110)
**NDIC11**	$(11\bar{9})$

SI-5. Thermal properties of NDIs: TGA and DSC study

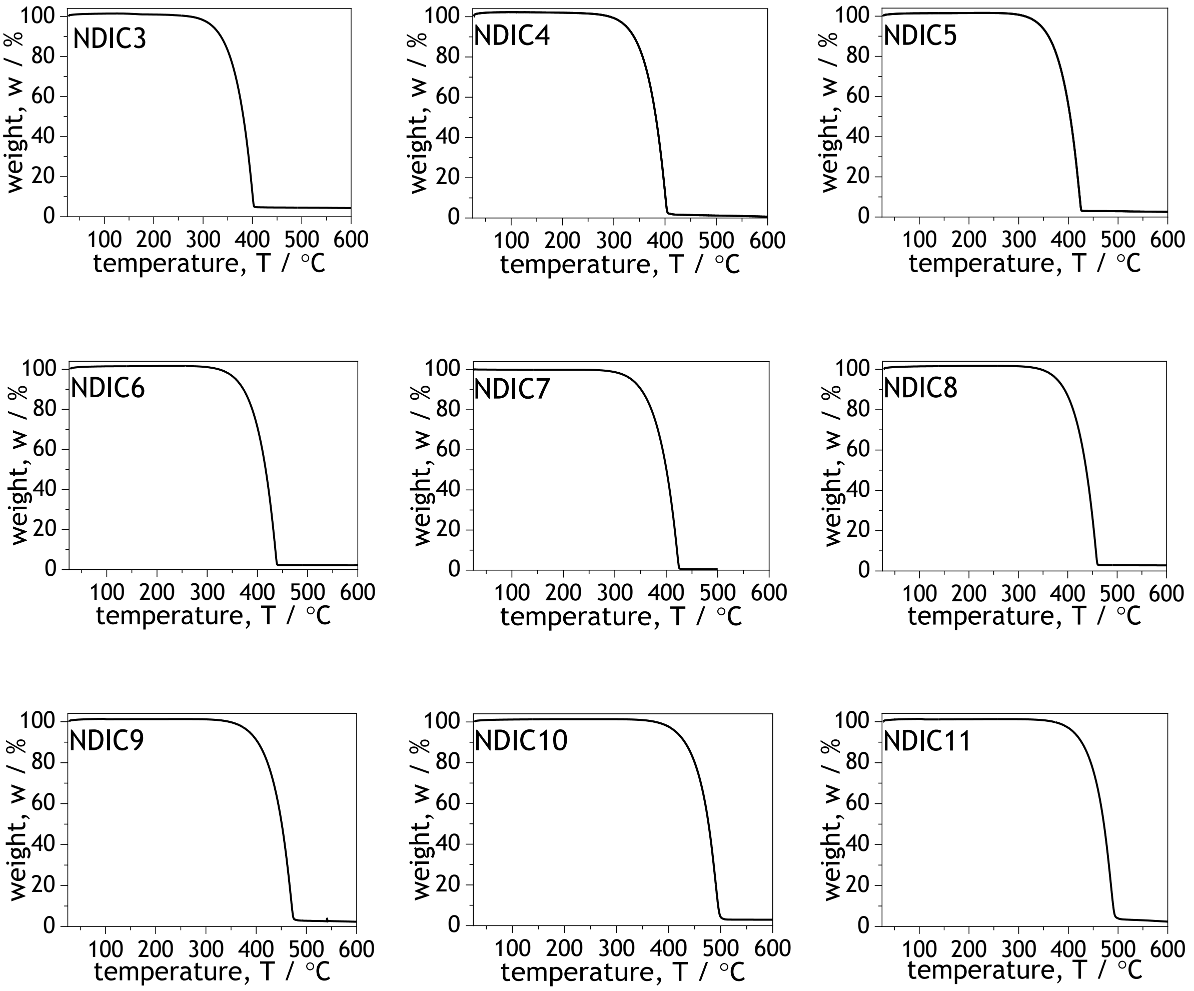

Figure S5. TGA curves of NDIs.

Table S9. Thermal decomposition temperatures of NDIs: temperatures of 3, 5 and 10 % weight loss

	weight loss		
	**3 %**	**5 %**	**10 %**
**NDIC3**	310	320	336
**NDIC4**	316	325	339
**NDIC5**	338	346	360
**NDIC6**	349	358	373
**NDIC7**	320	333	351
**NDIC8**	370	379	393
**NDIC9**	376	387	404
**NDIC10**	406	416	432
**NDIC11**	401	411	427

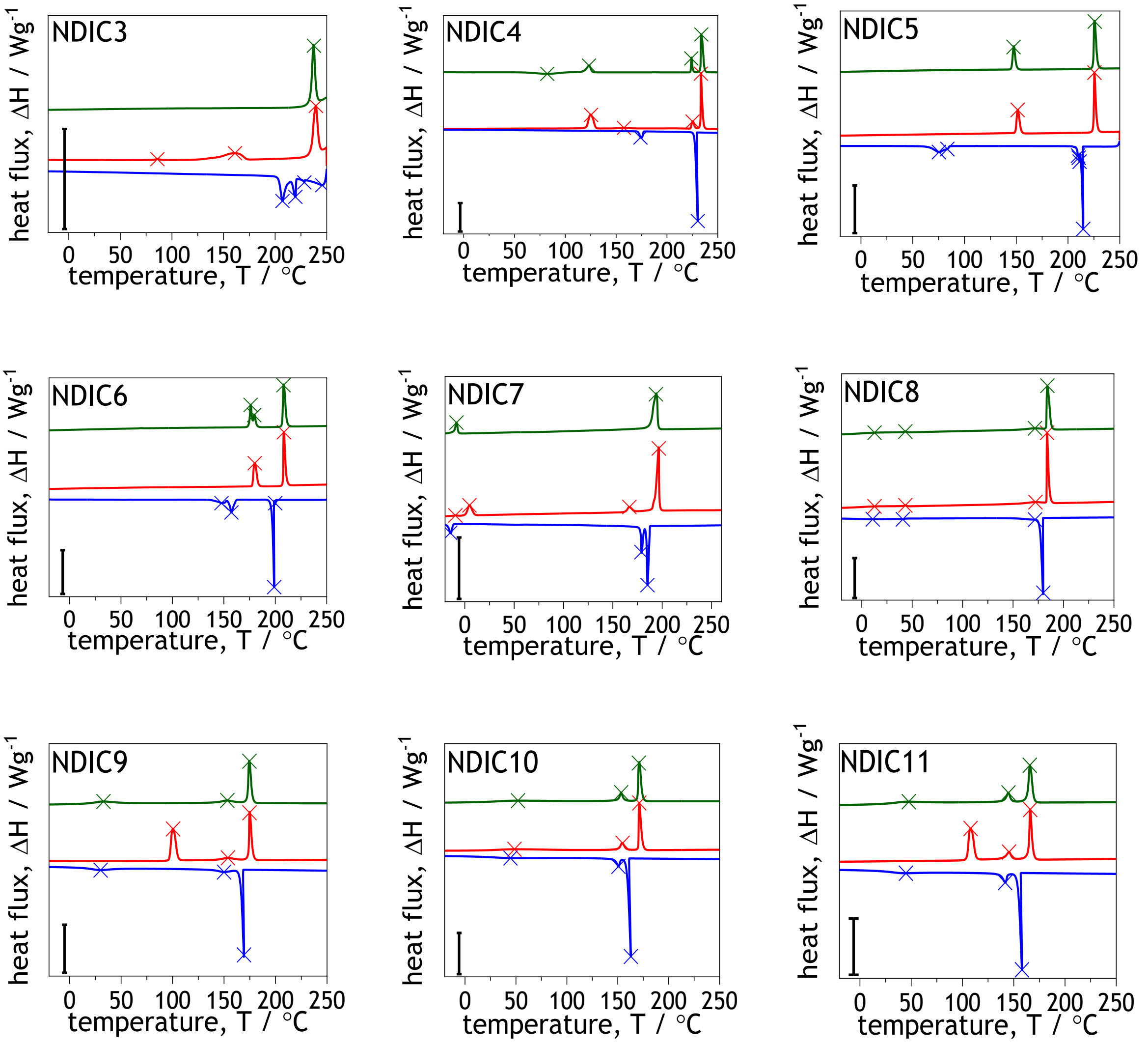

Figure S6. DSC plots for the 1st heating (red line) and 1st cooling (blue line) cycles, and 2nd heating (green line) cycle of NDIs. The heat flux scale bar in the lower left corner corresponds to 3.0 W/g. For the sake of clarity the curve of the 2nd heating cycle is vertically shifted.

Table S10. DSC data for the first heating and cooling cycles for the studied NDIs, together with literature data

	**1-st heating cycle**				**1-st cooling cycle**			
	**T (°C); ΔH (J/g)**	**T (°C); ΔH (J/g)**	**T (°C); ΔH (J/g)**	**T (°C); ΔH (J/g)**	**T (°C); ΔH (J/g)**	**T (°C); ΔH (J/g)**	**T (°C); ΔH (J/g)**	**T (°C); ΔH (J/g)**
**NDIC3**		85.9; 0.7	160.7; 27.9	239.3; 42.6	207.1; -19.5	219.6; -8.2	228.3; -0.7	245.3; -4.2
**NDIC4**	125.0; 50.0	157.1; 3.6	225.7; 13.0	233.5; 61.9			175.0; -14.2	230.3; -63.6
Lit. data:								
[SI1]	131.8; 18.2		227.2; 3.8	234.1; 21.7			169.7; -5.2	227.5; -22.2
[SI2]							160; -3	226; -23
**NDIC5**			151.5; 30.4	225.5; 60.0		83.1 and 75.6; -28.5 (bimodal)	211.2, 210.3 and 209.6; -4.4 (trimodal)	214.7; -33.6
**NDIC6**		180.2; 35.3		208.2; 50.5	148.6; -8.0	157.3;-18.8	198.9; -46.1	200.3; -1.1
Lit. data:								
[SI1]		178.5; 16.5		207.9; 22.2	49.1; -5.1	148.1; -13.0		200; -21.1
[SI3]		187; 29.5		208; 41.2				
[SI4]						150; -61.9		204; -92.2
**NDIC7**	-9.3; 0.6	4.7; 16.6	166.6; 7.4	196.5; 59.1	-14.3; -10.9		179.1; -15.0	185.1; -34.6
**NDIC8**	12.7; 5.3	43.2; 0.8	172.2; 4.8	183.7; 61.2	10.9; -5.2	40.6; -1.0	171.4*	179.8; -77.3*
Lit. data:								
[SI1]				190.0; 31.0				185.9; -30.7
[SI2]								181; -32
[SI5]								180; -40.3
[SI6]							171.1; -	185.1; -
**NDIC9**		100.3; 64.0	153.6; 8.6	174.9; 54.4		30.1;-20.6	150.0; -7.0	169.3; -54.8
**NDIC10**	48.7; 21.6	154.3; 16.8		171.0; 57.9	53.4; -20.4	150.8; -11.4		162.4; -57.8
Lit. data:								
[SI1]		163.1; 7.3		180.8; 34.6		160.3; -7.2		178.2; -30.2
**NDIC11**		107.9; 58.0	145.4; 12.9	166.1; 53.7	44.8; -20.5		141.7; -13.9	158.0; -55.1
Lit. data:								
[SI4]							144; -19.1	162; -72.7
[SI6]						104.5; -	148.2; -	169.8; -

*Due to overlapping of the peaks at 171.4 and 179.8 °C ΔH=-77.3 J/g was measured for both peaks together

Table S11. DSC data for the second heating cycle for the studied NDIs , together with literature data

	2-nd heating cycle			
	**T (°C); ΔH (J/g)**	**T (°C); ΔH (J/g)**	**T (°C); ΔH (J/g)**	**T (°C); ΔH (J/g)**
**NDIC3**				237.4; 39.8
**NDIC4**	82.0; -23.8	123.2; 23.2	224.1; 13.5	233.8; 63.6
Lit. data:				
[1]	123.0; 17.6	162.8; 1.1	225.2; 3.9	233.2; 18.8
**NDIC5**			147.7; 27.0	225.6; 52.1
**NDIC6**		176.2; 13.2	179.6; 5.6	208.3; 51.0
Lit. data:				
[1]	68.3; 4.5	173.5; 2.3	180.2; 7.1	208.0; 22.6
**NDIC7**	-8.3; 11.8			194.0; 56.4
**NDIC8**	12.6; 6.2	43.2; 0.8	171.7; 4.1	184.0; 61.0
Lit. data:				
[1]				192.7;30.5
**NDIC9**	32.9; 21.7		152.9; 9.1	174.4; 53.2
**NDIC10**	52.1; 23.5		153.2; 16.6	171.0; 56.2
Lit. data:				
[1]		163.2; 8.3	178; -	182.8; 31.3
[7]			153; 5.6	171; 25.6
**NDIC11**	47.3; 22.1		144.7; 15.2	165.6; 52.5
Lit. data:				
[7]			146; 7.5	166; 26.4

SI-6. Exemplary OFET characteristics of NDIs

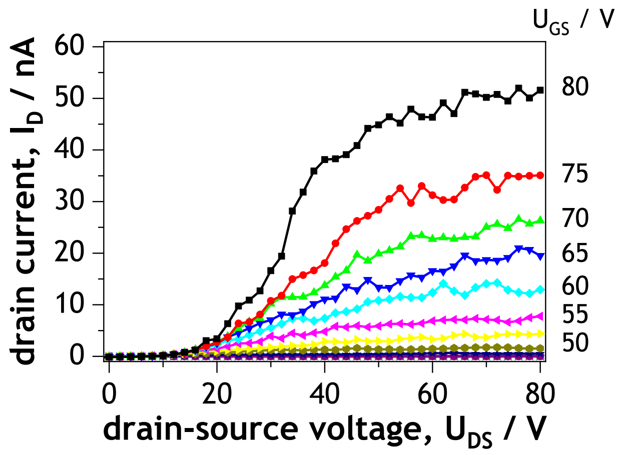

Figure S7. Exemplary output current-voltage characteristics for NDC7.

SI-7. Supporting references

SI1. Milita, S.; Liscio, F.; Cowen, L.; Cavallini, M.; Drain, B. A.; Degousée, T.; Luong, S.; Fenwick, O.; Guagliardi, A.; Schroeder, B. C.; Masciocchi, N., Polymorphism in N,N′-dialkyl-naphthalene diimides. *Journal of Materials Chemistry C* **2020,** 8, (9), 3097-3112.

SI2. Alvey, P. M.; Reczek, J. J.; Lynch, V.; Iverson, B. L., A Systematic Study of Thermochromic Aromatic Donor−Acceptor Materials. *The Journal of Organic Chemistry* **2010,** 75, (22), 7682-7690.

SI3. Ofir, Y.; Zelichenok, A.; Yitzchaik, S., 1,4;5,8-naphthalene-tetracarboxylic diimide derivatives as model compounds for molecular layer epitaxy. *Journal of Materials Chemistry* **2006,** 16, (22), 2142-2149.

SI4. Reczek, J. J.; Villazor, K. R.; Lynch, V.; Swager, T. M.; Iverson, B. L., Tunable Columnar Mesophases Utilizing C2 Symmetric Aromatic Donor−Acceptor Complexes. *Journal of the American Chemical Society* **2006,** 128, (24), 7995-8002.

SI5. Leight, K. R.; Esarey, B. E.; Murray, A. E.; Reczek, J. J., Predictable Tuning of Absorption Properties in Modular Aromatic Donor–Acceptor Liquid Crystals. *Chemistry of Materials* **2012,** 24, (17), 3318-3328.

SI6. Ichikawa, M.; Yokota, Y.; Jeon, H. G.; Banoukepa, G. D.; Hirata, N.; Oguma, N., Comparative study of soluble naphthalene diimide derivatives bearing long alkyl chains as n-type organic thin-film transistor materials. *Organic Electronics* **2013,** 14, (2), 516-522.

SI7. Jung, B. J.; Lee, K.; Sun, J.; Andreou, A. G.; Katz, H. E., Air-Operable, High-Mobility Organic Transistors with Semifluorinated Side Chains and Unsubstituted Naphthalenetetracarboxylic Diimide Cores: High Mobility and Environmental and Bias Stress Stability from the Perfluorooctylpropyl Side Chain. *Advanced Functional Materials* **2010,** 20, (17), 2930-2944.
